# Synthesis and Pharmacophore Modelling of 2,6,9-Trisubstituted Purine Derivatives and Their Potential Role as Apoptosis-Inducing Agents in Cancer Cell Lines

**DOI:** 10.3390/molecules20046808

**Published:** 2015-04-15

**Authors:** Jeannette Calderón-Arancibia, Christian Espinosa-Bustos, Álvaro Cañete-Molina, Ricardo A. Tapia, Mario Faúndez, Maria Jose Torres, Adam Aguirre, Margot Paulino, Cristian O. Salas

**Affiliations:** 1 Departamento de Química Orgánica, Facultad de Química, Pontificia Universidad Católica de Chile, 702843 Santiago de Chile, Chile; E-Mails: jeannettecalderon.a@gmail.com (J.C.-A.); ccespino@uc.cl (C.E.-B.); acanetem@uc.cl (A.C.-M.); rtapia@uc.cl (R.A.T.); 2 Departamento de Farmacia, Facultad de Química, Pontificia Universidad Católica de Chile, 702843 Santiago de Chile, Chile; E-Mails: mfaundeza@uc.cl (M.F.); mjtorres@uc.cl (M.J.T.); aaguirred@uc.cl (A.A.); 3 Centro de Bioinformática Estructural-DETEMA, Facultad de Química, Universidad de la República, C.C. 1157 Montevideo, Uruguay; E-Mail: margot.paulino@gmail.com

**Keywords:** antitumor, purine derivatives, apoptosis, pharmacophoric model

## Abstract

A series of 2,6,9-trisubstituted purine derivatives have been synthesized and investigated for their potential role as antitumor agents. Twelve compounds were obtained by a three step synthetic procedure using microwave irradiation in a pivotal step. All compounds were evaluated *in vitro* to determine their potential effect on cell toxicity by the MTT method and flow cytometry analysis on four cancer cells lines and Vero cells. Three out of twelve compounds were found to be promising agents compared to a known and effective anticancer drug, etoposide, in three out of four cancer cell lines assayed with considerable selectivity. Preliminary flow cytometry data suggests that compounds mentioned above induce apoptosis on these cells. The main structural requirements for their activity for each cancer cell line were characterized with a preliminary pharmacophore model, which identified aromatic centers, hydrogen acceptor/donor center and a hydrophobic area. These features were consistent with the cytotoxic activity of the assayed compounds.

## 1. Introduction

The purine framework is present in biological molecules that display key roles in signal pathways in all living organisms. Therefore, this heterocyclic structure represents an example of denominated “privilege scaffold”, a term that has been used to refer to the notion of multiple molecules of the same scaffold having bioactivity [1]. The purine scaffold is one the most abundant *N*-based heterocycle in nature and has been involved in a vast array of metabolic and other cellular processes [2] and the screening of purine libraries against a wide variety of biological targets has contributed to opening new applications as therapeutics agents [3]. Therefore, the purine scaffold is an interesting structural fragment that has been incorporated into new drugs. Some examples of major purine-based drugs that are currently being used are: anticancer agents (6-mercaptopurine, thioguanine) [4,5], antiviral agents to overcome infections such as herpes or AIDS (acyclovir, ganciclovir, carbovir, abavavir, ddI, *etc.*) [6,7,8,9] and agents to avoid organ rejection (azathioprine) [10].

**Figure 1:**
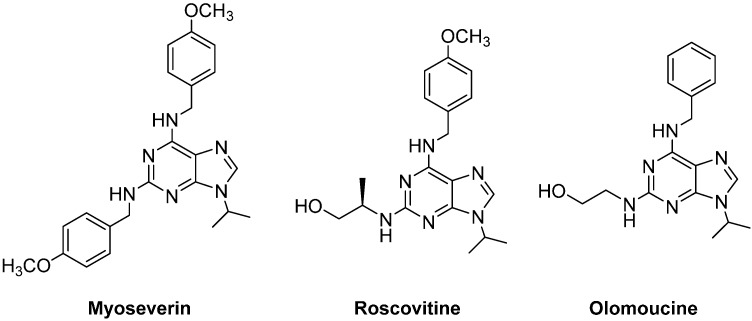
Chemical structures of 2,6,9-trisubstituted purines with biological properties.

In recent years, several di- and tri-substituted purine derivatives have been synthesized and tested on cancer cells. Schultz *et al.*, synthetized libraries of purine-based compounds that could modulate the activity of cyclin dependent kinases (CDKs). Given the essential role of CDKs in regulating the cell cycle, these compounds were very novel and promising as anticancer agents [11]. On the other hand, the same group has identified a 2,6,9-trisubstituted purine denominated myoseverin (Figure 1), a microtubule assembly inhibitor with moderate cytostatic activity, which may induce apoptosis [12]. Some pyrazolo[1,5-*a*]-1,3,5-triazine myoseverin derivatives have been synthesized and evaluated as antiproliferative agents [13]. Furthermore, other purine’s 2,6,9-substitution pattern has been designed and screened as CDK inhibitors, such as roscovitine (Figure 1) and related compounds [14,15,16]. Specifically, the pure *R*-enantiomer of roscovitine is under study as an oncology drug candidate in patients diagnosed with non-small cell lung cancer or other malignancies [17,18]. Finally, olomoucine (Figure 1), is another example of 2,6,9-trisubstituted purines, with antiproliferative effects on cancer cell lines [19,20].

Therefore, the search for new compounds with selective antitumor activity as an important driving force for the development and implementation of novel anticancer therapies, where the drug should exert a cytotoxic effect on malignant cells with a minimal effect on normal cells [21,22]. According to all the previous findings in the literature, a purine heterocycle scaffold is an interesting starting point to the identification of new antitumor compounds.

Because there is little data analyzing the influence of lipophilic properties of purine derivatives and their cytotoxic activity on normal and cancer cells, or their ability to induce apoptosis as part of a mechanism of action for the most active compounds; we decided to develop a new series of analogues to myoseverin and analyze the influence of the *N*-alkyl substitutions on the purine core. The hydrophobic moiety involved different chain lengths and special distribution, to correlate these molecular patterns with a potential antitumor activity. Compounds were tested on a panel of five cell lines, including Vero cells and four human-derived tumor cell lines, namely H1975 (lung), HL-60 (leukemia), HCT116 (colorectal) and HeLa. In addition, our study includes the elucidation of pharmacophore query for active molecules from a total of 12 compounds that led us to identify the principal structural features that might be responsible for their cytotoxicity.

## 2. Results and Discussion

### 2.1. Synthesis

**Scheme 1:**
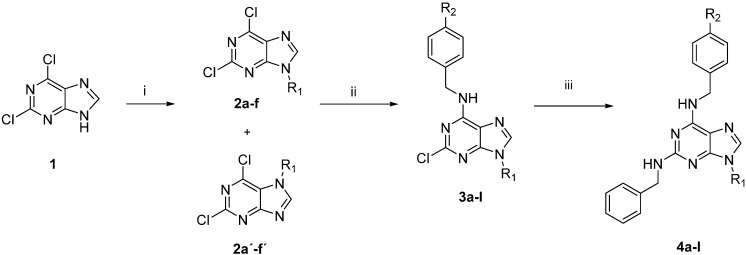
Synthesis of target compounds **4a**–**l**.

**Figure 2:**
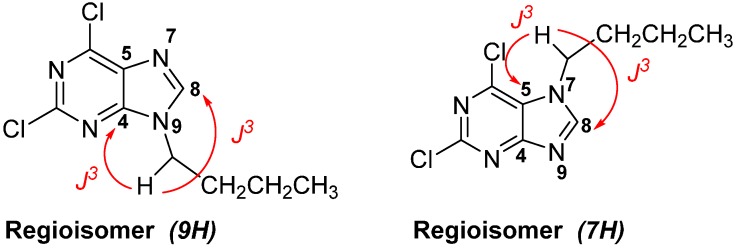
Structures of compounds **2b** and **2b'** and key HMBC correlations.

The preparation of the 2,6,9-trisubstituted purines listed in Table 1 is described in Scheme 1. This three step-sequence using 2,6-dichloropurine (**1**) as substrate is shown in Scheme 1 [23,24,25] and is a useful and direct approach for the synthesis of required compounds **4a**–**l** in the study. The first step was the alkylation of **1** with several alkyl halides under basic conditions, in DMF at room temperature to give a mixture of *N^9^*- and *N^7^*-alkylated purines regioisomers **2a**–**f**: **2a'**–**f'** in a proportion 4:1 in most cases. The unequivocal structure of each regioisomer was established by HMBC analysis (For selected compounds see supporting information) [26]. For example, for compound **2b**, the proton signal at 4.18 ppm (H-8) has correlations with the carbon signals at 145.88 ppm (C-5) and 153.16 ppm (C-3a), meanwhile the compound **2b'**,the proton signal at 4.42 ppm (H-8) has correlations with carbon signals at 121.59 ppm (C-6a) and 150.40 ppm (C-5), confirming the formation of both regioisomers (Figure 2).

The regiospecific S_N_Ar on C-6, was efficiently achieved using benzylamine or *p*-methoxybenzylamine as nucleophile, using 1-butanol as solvent and in presence of DIPEA by reflux by 12 h (yields 73%–99%) [27]. Finally, for the last S_N_Ar on C-2, the most unreactive site, we chose to use microwave irradiations (MW); since the displacement of this site needs harsh conditions (135 °C, 24 to 40 h) [16,28,29]. Therefore, the intermediates **3a**–**l** were substituted by benzylamine in 1-butanol and DIPEA as a base, under MW for 1 h, afforded 2,6,9-trisubstituted purines **4a**–**l** with medium to high yields (43%–95%). The structures of the new synthesized compounds were established on the basis of their spectral properties (IR, MS, ^1^H-NMR and ^13^C-NMR, see Supplementary Material).

**Table 1:** *In vitro* cytotoxicity of compounds **4a**–**4l** on cancer cell lines and Vero cells.

Entry	R_1_	R_2_	IC_50_ (µM) ^a^				
			H1975	HL-60	HCT116	HeLa	Vero
**4a**	*i*-Propyl	H	14.0 ± 0.26	26.0 ± 0.13	13.0 ± 0.12	32.0 ± 0.13	20.0 ± 0.11
**4b**	Butyl	H	28.0 ± 0.08	>50.0	7.2 ± 0.16	18.0 ± 0.14	5.6 ± 0.15
**4c**	*i*-Butyl	H	36.0 ± 0.27	>50.0	>50.0	>50.0	22.0 ± 0.23
**4d**	Pentyl	H	12.0 ± 0.19	>50.0	11.0 ± 0.11	10.1 ± 0.14	2.5 ± 0.20
**4e**	*i*-Pentyl	H	8.6 ± 0.18	>50.0	5.1 ± 0.14	2.7 ± 0.13	2.5 ± 0.14
**4f**	Hexyl	H	9.9 ± 0.18	>50.0	3.0 ± 0.12	5.3 ± 0.13	26.0 ± 0.12
**4g**	*i*-Propyl	OCH_3_	>50.0	22.0 ± 0.13	7.1 ± 0.11	10.7 ± 0.18	15.0 ± 0.23
**4h**	Butyl	OCH_3_	11.0 ± 0.25	>50.0	7.0 ± 0.16	6.3 ± 0.17	17.0 ± 0.20
**4i**	*i*-Butyl	OCH_3_	3.4 ± 0.28	>50.0	1.6 ± 0.12	4.9 ± 0.16	1.9 ± 0.24
**4j**	Pentyl	OCH_3_	2.3 ± 0.13	>50.0	8.0 ± 0.21	9.5 ± 0.14	26.0 ± 0.18
**4k**	*i*-Pentyl	OCH_3_	5.6 ± 0.23	>50.0	11.0 ± 0.26	5.4 ± 0.32	34.0 ± 0.13
**4l**	Hexyl	OCH_3_	>50.0	>50.0	6.8 ± 0.14	9.0 ± 0.13	15.0 ± 0.06
**Etoposide**	-	-	8.0 ± 0.33	6.2 ± 0.18	2.8 ± 0.24	8.2 ± 0.33	>25.0

^a^ IC_50_ values were determined in triplicate in the range of 0.05 to 50 µM.

*Reagents and conditions*: (i) alkyl halide, K_2_CO_3_, DMF, 6 h, rt, 51%–90% for **2a**–**f** and 15%–24% for **2a'**–**f'**; (ii) benzylamine or 4-methoxybenzylamine, DIPEA, 1-BuOH, 12 h, 110 °C, 73%–99%; (iii) benzylamine, DIPEA, 1-BuOH, 1 h, 150 °C, MW, 43%–95%. For compounds **4a**–**l**, the substituents R_1_ and R_2_ are indicated in Table 1.

### 2.2. Cytotoxic Studies

The potential *in vitro* antitumor activity of compounds **4a**–**l** was initially tested for their cytotoxic effects on H1975, HL-60, HCT116 and HeLa cancer cell lines and Vero cells. A conventional colorimetric assay was set up to estimate the IC_50_ values, which represent the concentration of a drug that is required for 50% inhibition *in vitro* after 72 h of continuous exposure to compounds. Four serial dilutions (from 12.5 to 100 µM) for each sample were evaluated in triplicate and etoposide was used as a positive control and reference drug.

Table 1 shows the IC_50_ values for cytotoxicity of compounds **4a**–**l** on Vero cells and cancer cell lines. In general, 2,6,9-trisubstituted purines activity was quite heterogeneous: For example, HL-60 cells seemed to be more resistant (all compounds tested with IC_50_ > 20 µM), while H1975, HCT116, Hela and Vero cells exhibited variable sensitivity. However, a preliminary analysis about the cytotoxicity indicates that: (i) Compound **4c** had no significant cytotoxic effects on four cancer cells (IC_50_ > 36 µM). (ii) Compounds **4b**, **4d**, **4e** and **4i** had no selectivity, affecting more Vero cells than cancer cells, although **4e** was the most potent compound against Hela cells (IC_50_ = 2.7 µM). (iii) Compounds **4a**, **4g**, **4h** and **4l** showed little activity, compared to etoposide, on the most cancer cells (except **4h** in Hela cells with IC_50_ = 6.3 µM). (iv) Compounds **4f**, **4j** and **4k**, were the most active exhibiting single-digit micromolar IC_50_ values and with the highest Selectivity Index (SI) values, against three cancer lines, as shown in the Table 2. It is important to consider that a potential antitumor drug must show low toxicity in mammalian host cells, and because of that, those more selective compounds are very promising for the development of new antitumor agents. These results are in agreement with the National Cancer Institute (NCI) protocols, where compounds exhibiting IC_50_ values <10 μM or 15 μM are considered active [30]. To establish a structure–relationship with these compounds, there are some preliminary conclusions that can be derived from these results.

A quick look at the IC_50_ values for each cancer cell line suggests a positive correlation with the lipophilicity of these compounds, which are in agreement with the aim to evaluate the effect on the cytotoxicity by the alkyl moiety or the methoxy group in the phenyl ring, on the purine scaffold. The lipophilicity of compounds **4a**–**l** could be estimated through the logarithm form (log*P*) the octanol-water partition coefficient (Table 2). However, no correlation was observed between compound cytotoxicity and their log*P* values in every cancer cell line. Although of no significance is the trend is that the compounds with low lipophilicity (log*P* > 5.3) exhibit the best antitumor activity.

The lack of correlation between lipophilicity and antitumor activity indicates that this parameter is not decisive in the cytotoxicity, and though is related with the membrane permeability, it does not always shows a quantitative correlation with this activity [26]. In fact, many other factors need to be considered in the relationship of structural pattern and cytotoxicity activity. Therefore, it is necessary to search for other tools to understand the antitumor activity *in silico* and to explore the structural requirements determining the observed biological properties [31,32].

**Table 2:** Selectivity Index and log*P* of compounds **4a**–**4l**.

Compound	Selectivity Index (SI) ^a^			Log *P* ^b^
	(Vero/H1975)	(Vero/HCT116)	(Vero/HeLa)	
**4a**	1.43	1.54	0.63	4.35
**4b**	0.20	0.78	0.31	4.94
**4c**	0.61	0.44	0.44	4.86
**4d**	0.21	0.23	0.25	5.38
**4e**	0.29	0.49	0.93	5.30
**4f**	2.63	8.67	4.91	5.83
**4g**	0.30	2.11	1.40	4.31
**4h**	1.55	2.43	2.70	4.90
**4i**	0.56	1.19	0.39	4.81
**4j**	11.30	3.25	2.74	5.34
**4k**	6.07	3.09	6.30	5.26
**4l**	0.30	2.21	1.67	5.78
Etoposide	3.10	4.03	3.05	-

^a^ Selectivity Index: expressed as the ratio IC_50_ (Vero)/IC_50_ (cancer cell line); ^b^ log*P* values calculated using MOE program.

### 2.3. Pharmacophore Elucidation

**Figure 3:**
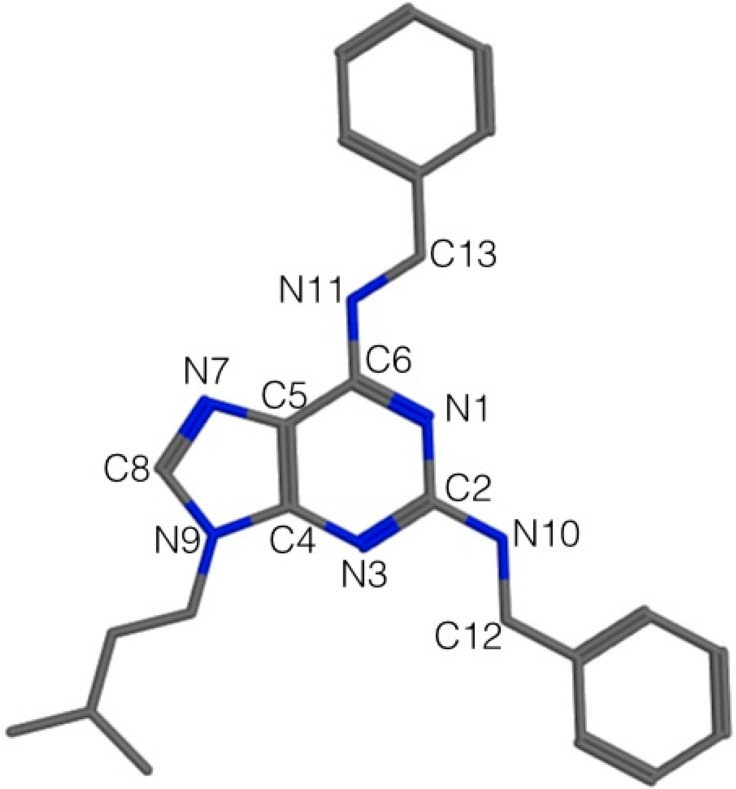
2D representation of **4e** and numbering used to identify the pharmacophoric features.

In order to generate a pharmacophore model (hypothesis) related with cytotoxicity (IC_50_) of compounds **4a**–**l** on H1975, HCT116 and Hela cells, the compound with the highest IC_50_ in every cancer cell line was chosen as a structural template. These compounds were **4e**, **4j** and **4i** for HeLa, H1975 and HCT116, respectively. The tridimensional structure of the selected molecule was created and then the pharmacophore hypothesis was generated using the polar-charged-hydrophobic (PHC) scheme of MOE program. Figure 3 shows the numbering used to identify pharmacophoric features in each model.

Each initial hypothesis was evaluated by scoring both active and inactive compounds. The inactive compounds were not involved in model generation, they only were used to discard hypothesis that did not distinguish between active or inactive compounds. This fact is especially useful when active compounds share similar structural pattern. For HeLa and H1975 cells, all molecules with IC_50_ lower than 17.9 µM were accepted as actives and all those with IC_50_ lower than 12.9 µM were accepted as actives for HTC116. Several structural particularities were identified in each model: *aromatic features* (Aro, centered in the purine and phenyl rings), *hydrophobic features* (Hyd, due that alkyl moiety bonded to N9 and the benzyl radicals), *acceptor features* (Acc/Acc2, generated by the presence of N1, N3 and N7 nitrogen atoms), and *donor features* (Don/Don2) centered on the N10 and N11 nitrogen atoms.

**Figure 4:**
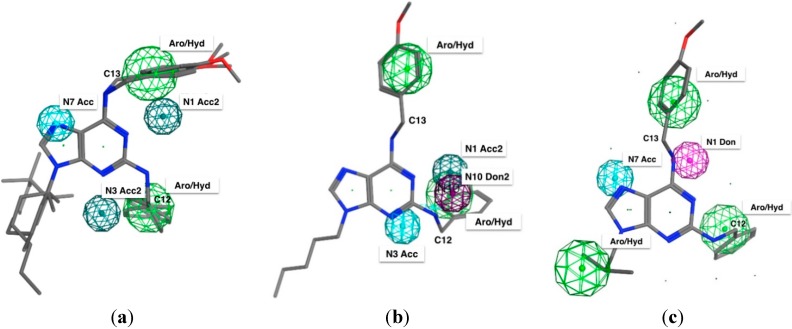
Elucidated pharmacophoric model for 2,6,9-trisubstituted purines against each cancer cell lines: (**a**) Hela; (**b**) H1975 and (**c**) HTC116.

The results for the complete elucidation strategy for each cancer cell line are summarized in Table 3 and Figure 4, where the essential features of each compound used in this study is seen. The most active compounds are also identified in every pharmacophoric model. For example: (i) *For cytotoxic compounds on Hela cells*, all features manually detected, five resulted associated with the most active compounds (**4d** and **4f**–**l**, Figure 4a), which is interpreted as a 91% of recovering and could be accepted by the pharmacophore as valid, selective and with the higher probability to recognize active and non-active molecules. (ii) *For cytotoxic compounds on H1975 cells,* five features resulted associated to the activity of these compounds and with the elucidated pharmacophore query, eight active compounds (**4a**, **4d**–**f**, **4h**–**k**) were recognized (Figure 4b), a similar behavior than HeLa cells. (iii) *For cytotoxic compounds on HTC116 cells*, five features resulted associated to the most active compounds, three common features with HeLa but, two new features were detected: an H-bond donor characteristic centered in the N11 nitrogen atom, and a hydrophobic region centered in the hydrocarbon moiety adjacent to the N9 nitrogen atom (Figure 4c). With this set of pharmacophoric features, a search was done within the purine database and the ten most active from the eleven molecules were selected. In this case, the **4a** analogue was rejected. This result proved the discriminatory capacity of the elucidated pharmacophore. However, these three models are preliminary, because a larger number of compounds will allow improved pharmacophore models.

**Table 3:** Pharmacophoric and structure features of the training set for each purine derivatives on cancer cell lines.

Structure Feature	Pharmacophoric Features		
	Hela	H1975	HTC116
**Purine N1**	* Acc	* Acc	-
**Purine N3**	* Acc	Acc	-
**Purine N7**	Acc	-	Acc
**Alkyl moiety on N9**	-	-	Aro/Hyd
**N10**	-	* Don	-
**N11**	-	-	Don
**Benzyl moiety on C2**	Aro/Hyd	Aro/Hyd	Aro/Hyd
**Benzyl moiety on C2**	Aro/Hyd	Aro/Hyd	Aro/Hyd

* These are the named “projected features” and correspond to regions in which the lone pairs could be located, making possible the hydrogen bonding.

### 2.4. Apoptosis Assay

**Figure 5:**
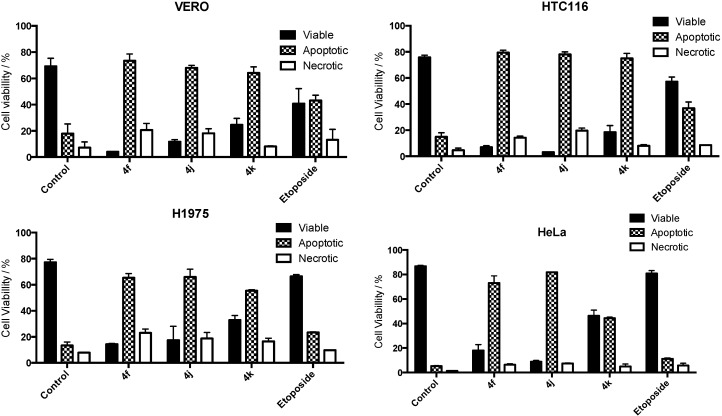
Viability assays of compounds **4f**, **4j** and **4k** on cancer cell lines.

Finally, to study the mechanism involved on the antitumor activity of the most active compounds (**4f**, **4j** and **4k**), the effects on the apoptosis were evaluated by flow cytometry (FACS) by staining with propidium iodide (PI). To evaluate sensitivity to **4f**, **4j** and **4k**, Vero, HTC116, H1975 and Hela were treated with concentrations of 50 µM for 16 h. Fifty µM etoposide was used as positive control. All cells were completely sensitive to all compounds at the concentration used and the results are shown in Figure 5. As it can be seen in Figure 5, **4f**, **4j** and **4k** induced apoptosis as a prevalent type of cell death, in high percentages (>70%), in every cancer cell line. Moreover, **4f**, **4j**, and **4k** induced an apoptotic cell response greater than etoposide.

## 3. Experimental Section

### 3.1. General Information

2,6-Dichloropurine, benzyl amines and the alkyl halides, were purchased from Sigma-Aldrich (St. Louis, MO, USA). Cell culture medium, fetal bovine serum and penicillin-streptomycin were purchased from Biological Industries (Kibbutz Beit Haemek, Israel). Etoposide, 3-(4,5-dimethylthiazol-2-yl)-2,5-diphenyl tetrazolium bromide (MTT) propidium iodide and all other chemicals were obtained from Sigma-Aldrich. Microwave-assisted reactions were carried out in a Microwave Synthesis Reactor Monowave 300 (Anton Paar GmbH, Graz, Austria), in 10 mL sealed vials. Melting points were determined on a Thermogeräte Kofler apparatus (Reichert, Werke A.G., Wien, Austria) and were uncorrected. Infrared spectra were recorded on a Vector 22 spectrophotometer (Bruker Optik GmbH, Bremen, Germany) using KBr discs. Nuclear magnetic resonance spectra were recorded on a Bruker AM-400 apparatus using CDCl_3_ solutions containing TMS as internal standard. HPLC-MS experiments were performed on an Exactive Plus Orbitrap MS (Bremen, Germany). Mass spectra were obtained on an HP 5988A spectrometer (Hewlett-Packard, Palo Alto, CA, USA). Thin layer chromatography (TLC) was performed using Merck GF-254 type 60 silica gel. Column chromatography was carried out using Merck silica gel 60 (70–230 mesh).

### 3.2. General Synthetic Procedure to Obtain N-Alkyl Purines ***2a***–***f*** and ***2a'***–***f'***

A mixture of 2,6-dichloropurine **1** (1.0 mmol), the respective alkyl halide (1.5 mmol), and potassium carbonate (3.0 mmol) in DMF (5 mL) was stirred for 6 h, then the mixture was filtered and evaporated under vacuum. The products were separated by flash chromatography on silica gel eluting with EtOAc/CH_2_Cl_2_ (2:3).

*2,6-Dichloro-9-isopropyl-9H-purine* (**2a**): White solid, yield (67%), mp 100–102 °C. ^1^H-NMR (CDCl_3_) δ 8.17 (s, 1H), 4.97–4.86 (m, 1H), 1.65 (d, *J* = 6.8 Hz, 6H). ^13^C-NMR (CDCl_3_) δ 152.73, 151.66, 146.93, 143.58, 131.05, 48.36, 22.51 (2C). IR (KBr, cm^−1^): 2985, 1588, 1555, 1358, 1216, 872. HRMS for (C_8_H_8_Cl_2_N_4_ [M+H]^+^). Calcd: 231.0204. Found: 231.0195.

*2,6-Dichloro-7-isopropyl-9H-purine* (**2a'**): White solid, yield (20%), mp 106–108 °C. ^1^H-NMR (CDCl_3_) δ 8.17 (s, 1H), 4.97–4.86 (m, 1H), 1.65 (d, *J* = 6.8 Hz, 6H). ^13^C-NMR (CDCl_3_) δ 152.73, 151.66, 146.93, 143.58, 131.05, 48.36, 22.51 (2C). IR (KBr, cm^−1^): 2990, 1584, 1557, 1364. HRMS for (C_8_H_8_Cl_2_N_4_ [M+H]^+^). Calcd: 231.0204. Found: 231.0180.

*9-Butyl-2,6-dichloro-9H-purine* (**2b**): White solid, yield (52%), mp 61–63 °C. ^1^H-NMR (CDCl_3_) δ 8.10 (s, 1H), 4.27 (t, *J* = 7.3 Hz, 2H), 2.00–1.80 (m, 2H), 1.51–1.27 (m, 2H), 0.98 (t, *J* = 7.4 Hz, 3H). ^13^C-NMR (CDCl_3_) δ 153.20, 152.89, 151.71, 145.74, 130.74, 44.42, 31.73, 19.81, 13.42. IR (KBr, cm^−1^): 2954, 1596, 1556, 1347, 1228, 878. HRMS for (C_9_H_10_Cl_2_N_4_ [M+H]^+^). Calcd: 245.0361. Found: 245.0352.

*7-Butyl-2,6-dichloro-7H-purine* (**2b'**) White solid, yield (18%), mp 78–79 °C. ^1^H-NMR (CDCl_3_) δ 8.20 (s, 1H), 4.40 (t, *J* = 7.3 Hz, 2H), 1.85–1.80 (m, 2H), 1.35–1.27 (m, 2H), 0.89 (t, *J* = 7.1 Hz, 3H). ^13^C-NMR (CDCl_3_) δ 163.57, 151.49, 145.95, 130.69, 121.57, 47.45, 33.48, 19.57, 13.44. IR (KBr, cm^−1^): 2952, 1601, 1550, 1229. HRMS for (C_9_H_10_Cl_2_N_4_ [M+H]^+^). Calcd: 245.0361. Found: 245.0335.

*2,6-Dichloro-9-isobutyl-9H-purine* (**2c**): White solid, yield (61%), mp 40–43 °C. ^1^H-NMR (CDCl_3_) δ 8.08 (s, 1H), 4.08 (d, *J* = 7.4 Hz, 2H), 2.34–2.24 (m, 1H), 0.98 (d, *J* = 6.7 Hz, 6H). ^13^C-NMR (CDCl_3_) δ 153.39, 152.95, 151.75, 146.10, 130.65, 51.78, 28.98, 19.84 (2C). IR (KBr, cm^−1^): 2954, 1594, 1558, 1352, 1232, 879. HRMS for (C_9_H_10_Cl_2_N_4_ [M+H]^+^). Calcd: 245.0361. Found: 245.0351.

*2,6-Dichloro-7-isobutyl-7H-purine* (**2c'**): White solid, yield (22%), mp 94–96 °C. ^1^H-NMR (CDCl_3_) δ 8.19 (s, 1H), 4.20 (d, *J* = 7.4 Hz, 2H), 2.19–2.08 (m, 1H), 0.91 (d, *J* = 6.7 Hz, 6H). ^13^C-NMR (CDCl_3_) δ 163.59, 152.89, 150.81, 143.73, 121.68, 54.69, 30.50, 19.55. IR (KBr, cm^−1^): 2955, 1596, 1558, 1349, 1231. HRMS for (C_9_H_10_Cl_2_N_4_ [M+H]^+^). Calcd: 245.0361. Found: 245.0336.

*2,6-Dichloro-9-pentyl-9H-purine* (**2d**): White solid, yield (51%), mp 37–41 °C. ^1^H-NMR (CDCl_3_) δ 8.10 (s, 1H), 4.26 (t, *J* = 7.3 Hz, 2H), 1.99–1.83 (m, 2H), 1.44–1.20 (m, 4H), 0.91 (t, *J* = 7.1 Hz, 3H). ^13^C-NMR (CDCl_3_) δ 153.17, 152.77, 151.58, 145.83, 130.70, 44.66, 29.41, 28.60, 22.01, 13.77. IR (KBr, cm^−1^): 2958, 1596, 1554, 1351, 1235, 876. HRMS for (C_10_H_12_Cl_2_N_4_ [M+H]^+^). Calcd: 259.0517. Found: 259.0506.

*2,6-Dichloro-7-pentyl-9H-purine* (**2d'**): Light yellow oil (24%). ^1^H-NMR (CDCl_3_) δ 8.25 (s, 1H), 4.40 (t, *J* = 7.4 Hz, 2H), 1.87–1.79 (m, 2H), 1.28–1.20 (m, 4H), 0.86–0.73 (m, 3H). ^13^C-NMR (CDCl_3_) δ 163.52, 152.71, 150.57, 143.63, 121.57, 47.69, 31.22, 28.38, 22.03, 13.78. IR (KBr, cm^−1^): 2955, 1598, 1554, 1350, 1236. HRMS for (C_10_H_12_Cl_2_N_4_ [M+H]^+^). Calcd: 259.0517. Found: 259.0490.

*2,6-Dichloro-9-isopentyl-9H-purine* (**2e**): White solid, yield (56%), mp 77–79 °C. ^1^H-NMR (CDCl_3_) δ 8.11 (s, 1H), 4.31–4.26 (m, 2H), 1.82 (dd, *J* = 15.0, 7.1 Hz, 2H), 1.60 (td, *J* = 13.4, 6.7 Hz, 1H), 1.00 (d, *J* = 6.6 Hz, 6H). ^13^C-NMR (CDCl_3_) δ 153.18, 152.85, 151.66, 145.64, 130.71, 43.00, 38.46, 25.63, 22.17 (2C). IR (KBr, cm^−1^): 2956, 1596, 1555, 1347, 874. HRMS for (C_10_H_12_Cl_2_N_4_ [M+H]^+^). Calcd: 259.0517. Found: 259.0508.

*2,6-Dichloro-7-isopentyl-7H-purine* (**2e'**): White oil, yield (15%). ^1^H-NMR (CDCl_3_) δ 8.23 (s, 1H), 4.44–4.39 (m, 2H), 1.74 (q, 7.1 Hz, 2H), 1.68–1.52 (m, 1H), 0.92 (d, *J* = 6.7 Hz, 6H). ^13^C-NMR (CDCl_3_) δ 163.55, 152.79, 150.37, 143.59, 121.55, 46.14, 40.43, 25.72, 22.24 (2C). IR (KBr, cm^−1^): 2962, 1596, 1558, 1351, 1237. HRMS for (C_10_H_12_Cl_2_N_4_ [M+H]^+^). Calcd: 259.0517. Found: 259.0490.

*2,6-Dichloro-9-hexyl-9H-purine* (**2f**): White solid, yield (58%), mp 35–37 °C. ^1^H-NMR (CDCl_3_) δ 8.10 (s, 1H), 4.29–4.23 (m, 2H), 1.92–1.76 (m, 2H), 1.41–1.24 (m, 6H), 0.89 (t, *J* = 7.0 Hz, 3H). ^13^C-NMR (CDCl_3_) δ 153.18, 152.84, 151.65, 145.79, 130.73, 44.68, 31.05, 29.71, 26.20, 22.38, 13.88. IR (KBr, cm^−1^): 2953, 2922, 1599, 1556, 1347, 1217, 878. HRMS for (C_11_H_14_Cl_2_N_4_ [M+H]^+^). Calcd: 273.0674. Found: 273.0659.

*2,6-Dichloro-7-hexyl-9H-purine* (**2f'**): Light yellow oil, yield (23%). ^1^H-NMR (CDCl_3_) δ 8.24 (s, 1H), 4.41 (t, *J* = 7.4 Hz, 2H), 1.93–1.71 (m, 2H), 1.40–1.10 (m, 6H), 0.78 (t, *J* = 7.0 Hz, 3H). ^13^C-NMR (CDCl_3_) δ 163.53, 152.83, 150.47, 143.65, 121.57, 47.73, 31.50, 31.05, 25.98, 22.36, 13.85. IR (KBr, cm^−1^): 2954, 1597, 1554, 1350, 1228. HRMS for (C_11_H_14_Cl_2_N_4_ [M+H]^+^). Calcd: 273.0674. Found: 273.0646.

### 3.3. General Synthetic Procedure to Obtain N-Benzyl-9-Alkyl-2-Chloro-9H-Purin-6-Amines ***3a***–***l***

DIPEA (1.8 mmol) and the respective benzylamine (0.9 mmol) were added to a solution of the corresponding 9-alkyl-2,6-dichloro-9*H*-purine (**2a**–**2f**, 1.00 mmol) in 1-butanol (5 mL) and the reaction mixture was heated at 110 °C overnight. Then, the solvent was removed under vacuum and the crude product was purified by column chromatographic on silica gel using as eluent a mixture 4:1 of chloroform/acetone for all derivatives.

*N-Benzyl-2-chloro-9-isopropyl-9H-purin-6-amine* (**3a**): White solid, yield (95%), mp 167–170 °C. ^1^H-NMR (CDCl_3_) δ 7.66 (s, 1H), 7.40–7.30 (m, 5H), 6.53 (s, 1H), 4.84–4.79 (m, 3H), 1.56 (d, *J* = 6.8 Hz, 6H). ^13^C-NMR (CDCl_3_) δ 154.93, 137.57 (2C), 128.66 (2C), 127.88, 127.86, 127,54, 127.56, 127.08, 118.48, 46.92, 44.59, 22.74 (2C). IR (KBr, cm^−1^): 3265, 1625, 1571, 1311. HRMS for (C_15_H_16_ClN_5_ [M+H]^+^). Calcd: 302.1172. Found: 302.1164.

*N-Benzyl-9-butyl-2-chloro-9H-purin-6-amine* (**3b**): White solid, yield (90%), mp 160–162 °C. ^1^H-NMR (CDCl_3_) δ 7.48 (s, 1H), 7.42–7.17 (m, 5H), 6.66 (s, 1H), 4.82 (br, 2H), 4.11 (t, *J* = 7.2 Hz, 2H), 1.87–1.77 (m, 2H), 1.38–1.30 (m, 2H), 0.95 (t, *J* = 7.4 Hz, 3H). ^13^C-NMR (CDCl_3_) δ 155.57, 140.52 (2C), 138.42, 131.32, 129.25, 129.11(2C), 128.35 (2C), 128.04, 44.06 (2C), 32.37, 20.20, 13.89. IR (KBr, cm^−1^): 3262, 1627, 1352. HRMS for (C_16_H_18_ClN_5_ [M+H]^+^). Calcd: 316.1329. Found: 316.1316.

*N-Benzyl-2-chloro-9-isobutyl-9H-purin-6-amine* (**3c**): White solid, yield (81%), mp 155–157 °C. ^1^H-NMR (CDCl_3_) δ 7.39–7.24 (m, 6H), 6.93 (s, 1H), 4.82 (s, 2H), 3.90 (d, *J* = 3.4 Hz, 2H), 2.30–2.06 (m, 1H), 0.92 (d, *J* = 6.5 Hz, 6H). ^13^C-NMR (CDCl_3_) δ 155.58, 140.97 (2C), 138.46, 129.09 (2C), 128.33 (2C), 128.02 (2C), 118.91, 51.51, 45.13, 29.33, 20.25 (2C). IR (KBr, cm^−1^): 3262, 1631, 1352. HRMS for (C_16_H_18_ClN_5_ [M+H]^+^). Calcd: 316.1329. Found: 316.1318.

*N-Benzyl-2-chloro-9-pentyl-9H-purin-6-amine* (**3d**): White solid, yield (92%), mp 110–113 °C. ^1^H-NMR (CDCl_3_) δ 7.42–7.20 (m, 6H), 7.00 (br, 1H), 4.82 (br, 2H), 4.08 (br, 2H), 1.88–1.71 (m, 2H), 1.40–1.18 (m, 4H), 0.89 (t, *J* = 7.1 Hz, 3H). ^13^C-NMR (CDCl_3_) δ 155.58, 140.54 (2C), 138.53, 129.06 (2C), 128.31 (2C), 127.98 (2C), 118.98, 45.05, 44.29, 30.03, 29.07, 22.52, 14.26. IR (KBr, cm^−1^): 3264, 1628, 1351. HRMS for (C_17_H_20_ClN_5_ [M+H]^+^). Calcd: 330.1485. Found: 330.1472.

*N-Benzyl-2-chloro-9-isopentyl-9H-purin-6-amine* (**3e**): White solid, yield (93%), mp 154–155 °C. ^1^H-NMR (CDCl_3_) δ 7.57–7.20 (m, 6H), 7.00 (br, 1H), 4.82 (s, 2H), 4.11 (d, *J* = 5.2 Hz, 2H), 1.72 (br, 2H), 1.63–1.50 (m, 1H), 0.96 (dd, *J* = 6.5, 1.8 Hz, 6H). ^13^C-NMR (CDCl_3_) δ 155.62, 140.38 (2C), 138.54, 129.10, 129.08, 128.34 (2C), 128.04, 128.00, 118.98, 45.08, 42.61, 39.05, 25.96, 22.65 (2C). IR (KBr, cm^−1^): 3264, 1629, 1306. HRMS for (C_17_H_20_ClN_5_ [M+H]^+^). Calcd: 330.1485. Found: 330.1473.

*N-Benzyl-2-chloro-9-hexyl-9H-purin-6-amine* (**3f**): White solid, yield (85%), mp 98–100 °C. ^1^H-NMR (CDCl_3_) δ 7.40–7.23 (m, 6H), 6.95 (s, 1H), 4.82 (s, 2H), 4.26–3.97 (m, 2H), 1.95–1.73 (m, 2H), 1.28 (br, 6H), 0.88 (t, *J* = 6.1 Hz, 3H). ^13^C-NMR (CDCl_3_) δ 155.57, 140.52 (2C), 138.51, 129.09, 129.07, 128.33, 128.32, 128.04, 128.00, 118.99, 45.06, 44.32, 31.58, 30.32, 26.64, 22.84, 14.33. IR (KBr, cm^−1^): 3265, 1630, 1306. HRMS for (C_18_H_22_ClN_5_ [M+H]^+^). Calcd: 344.1642. Found: 344.1631.

*2-Chloro-9-isopropyl-N-(4-methoxybenzyl)-9H-purin-6-amine* (**3g**): White solid, yield (99%), mp 140–141 °C. ^1^H-NMR (CDCl_3_) δ 7.37 (s, 1H), 7.25 (d, *J* = 7.3 Hz, 2H), 6.83 (d, *J* = 7.5 Hz, 2H), 4.85–4.61 (m, 3H), 3.78 (s, 3H), 1.50 (d, *J* = 5.5 Hz, 6H). ^13^C-NMR (CDCl_3_) δ 159.47, 155.56, 138.02 (2C), 130.66 (2c), 129.60 (2C), 119.17, 114.41 (2C), 55.66, 47.26, 44.44, 23.10 (2C). IR (KBr, cm^−1^): 3268, 2972, 1643, 1513, 1291, 1250. HRMS for (C_16_H_18_ClN_5_O [M+H]^+^). Calcd: 332.1278. Found: 332.1263.

*9-Butyl-2-chloro-N-(4-methoxybenzyl)-9H-purin-6-amine* (**3h**): White solid, yield (82%), mp 149–150 °C. ^1^H-NMR (CDCl_3_) δ 7.55 (s, 1H), 7.28 (dd, *J* = 7.1, 5.2 Hz, 2H), 6.89–6.82 (m, 2H), 6.63 (s, 1H), 4.73 (s, 2H), 4.12 (t, *J* = 7.2 Hz, 2H), 3.80 (s, 3H), 1.88–1.76 (m, 2H), 1.42–1.19 (m, 2H), 0.95 (t, *J* = 7.4 Hz, 3H). ^13^C-NMR (CDCl_3_) δ 159.57, 155.44, 140.39 (2C), 130.39, 129.81, 129.77 (2C), 129,73, 114.48 (2C), 55.69, 44.06 (2C), 32.37, 20.20, 13.88. IR (KBr, cm^−1^): 3262, 2953, 1625, 1513, 1305, 1257. HRMS for (C_17_H_20_ClN_5_O [M+H]^+^). Calcd: 346.1435. Found: 346.1424.

*2-Chloro-9-isobutyl-N-(4-methoxybenzyl)-9H-purin-6-amine* (**3i**): White solid, yield (81%), mp 115–120 °C. ^1^H-NMR (CDCl_3_) δ 7.47 (s, 1H), 7.34–7.22 (m, 2H), 6.91–6.79 (m, 2H), 6.66 (s, 1H), 4.73 (s, 2H), 3.92 (s, 2H), 3.80 (s, 3H), 1.42–1.11 (m, 1H), 0.94–0.91 (m, 6H). ^13^C-NMR (CDCl_3_) δ 159.57, 155.48, 140.87 (2C), 130.42, 130.39, 129.76 (2C), 114,49, 114.48 (2C), 55.69, 51.50 (2C), 29.34, 20.25 (2C). IR (KBr, cm^−1^): 3265, 2956, 1625, 1513, 1305, 1253. HRMS for (C_17_H_20_ClN_5_O [M+H]^+^). Calcd: 346.1435. Found: 346.1424.

*2-Chloro-N-(4-methoxybenzyl)-9-pentyl-9H-purin-6-amine* (**3j**): White solid, yield (83%), mp 124–127 °C. ^1^H-NMR (CDCl_3_) δ 7.47 (s, 1H), 7.30–7.23 (m, 2H), 6.85 (d, *J* = 8.6 Hz, 2H), 6.72 (s, 1H), 4.73 (s, 2H), 4.10 (t, *J* = 7.2 Hz, 2H), 3.80 (s, 3H), 1.90–1.76 (m, 2H), 1.43–1.16 (m, 4H), 0.89 (t, *J* = 7.1 Hz, 3H). ^13^C-NMR (CDCl_3_) δ 159.56, 155.45, 140.45 (2C), 130.40, 129.81, 129.74 (2C), 129.66, 114.45 (2C), 55.69, 44.29 (2C), 30.05, 29.08, 22.52, 14.25. IR (KBr, cm^−1^): 3262, 2919, 1626, 1513, 1304, 1243. HRMS for (C_18_H_22_ClN_5_O [M+H]^+^). Calcd: 360.1591. Found: 360.1559.

*2-Chloro-9-isopentyl-N-(4-methoxybenzyl)-9H-purin-6-amine* (**3k**): White solid, yield (73%), mp 106–110 °C. ^1^H-NMR (CDCl_3_) δ 7.53 (s, 1H), 7.34–7.23 (m, 2H), 6.88–6.82 (m, 2H), 6.66 (s, 1H), 4.73 (s, 2H), 4.18–4.08 (m, 2H), 3.80 (s, 3H), 1.79–1.67 (m, 2H), 1.64–1.51 (m, 1H), 0.97 (d, *J* = 6.6 Hz, 6H). ^13^C-NMR (CDCl_3_) δ 159.56, 155.46, 140.26 (2C), 130.40, 129.83, 129.74 (2C), 118.93, 114.47 (2C), 55.69, 42.62 (2C), 39.07, 25.97, 22.64 (2C). IR (KBr, cm^−1^): 3264, 2953, 1625, 1513, 1306, 1242. HRMS for (C_18_H_22_ClN_5_O [M+H]^+^). Calcd: 360.1591. Found: 360.1559.

*2-Chloro-9-hexyl-N-(4-methoxybenzyl)-9H-purin-6-amine* (**3l**): White solid, yield (74%), mp 83–86 °C. ^1^H-NMR (CDCl_3_) δ 7.53 (s, 1H), 7.28 (dd, *J* = 8.9, 1.9 Hz, 2H), 6.90–6.79 (m, 2H), 6.59 (s, 1H), 4.73 (s, 2H), 4.11 (t, *J* = 7.2 Hz, 2H), 3.83–3.75 (m, 3H), 1.83 (d, *J* = 5.8 Hz, 2H), 1.38–1.23 (m, 6H), 0.88 (t, *J* = 6.8 Hz, 3H). ^13^C-NMR (CDCl_3_) δ 159.56, 155.45, 140.42 (2C), 130.39, 129.84, 129.76, 129.70, 118.95, 114.47 (2C), 55.69, 44.32 (2C), 31.58, 30.34, 26.64, 22.84, 14.32. IR (KBr, cm^−1^): 3266, 2955, 1625, 1513, 1306, 1256. HRMS for (C_19_H_24_ClN_5_O [M+H]^+^). Calcd: 374.1478. Found: 374.1714.

### 3.4. General Synthetic Procedure to Obtain N^2^,N^6^-Dibenzyl-9-Alkyl-9H-Purine-2,6-Diamine Derivatives ***4a***–***l***

The *N*-benzyl-9-alkyl-2-chloro-9*H*-purin-6-amine **3a**–**l** **(**1.0 mmol), benzylamine (0.9 mmol), DIPEA (2.0 mmol) and 1-butanol (3 mL) were added to a microwave reaction flask and the reaction mixture was irradiated for 1 hour at 150 °C. Then the solvent was evaporated under vacuum and the crude product was purified by column chromatographic on silica gel using chloroform as eluent.

*N^2^,N^6^-Dibenzyl-9-isopropyl-9H-purine-2,6-diamine* (**4a**): White solid, yield (95%), mp 74–76 °C. ^1^H-NMR (CDCl_3_) δ 7.39–7.22 (m, 11H), 6.35 (br, 1H), 6.08 (br, 1H), 4.62–4.57 (m, 3H), 4.40 (d, *J* = 5.5 Hz, 2H), 1.48 (d, *J* = 6.8 Hz, 6H). ^13^C-NMR (CDCl_3_) δ 170.34, 159.82, 155.32, 140.88, 139.82, 138.76, 134.94, 129.09, 128.89, 128.75, 128.25, 128.10, 128.06, 127.90, 127.51, 127.22, 115.13, 46.62, 46.44, 44.12, 22.93 (2C). IR (KBr, cm^−1^): 3289, 2922, 1629, 1548, 1352. HRMS for (C_22_H_24_N_6_ [M+H]^+^). Calcd: 373.2141. Found: 373.2127.

*N^2^,N^6^-Dibenzyl-9-butyl-9H-purine-2,6-diamine* (**4b**): White solid, yield (65%), mp 113–115 °C. ^1^H-NMR (CDCl_3_) δ 7.42–7.24 (m, 11H), 6.11 (br, 1H), 5.88 (br, 1H), 4.88–4.53 (m, 4H), 3.98 (t, *J* = 7.1 Hz, 2H), 1.86–1.66 (m, 2H), 1.41–1.16 (m, 2H), 0.92 (t, *J* = 7.3 Hz, 3H). ^13^C-NMR (CDCl_3_) δ 160.02, 155.30, 140.84, 139.71, 137.38, 129.49, 129.12, 128.90, 128.75, 128.27, 128.15, 128.06, 127.54, 127.23, 114.81, 114.31, 46.43, 44.17, 43.34, 32.31, 20.23, 13.94. IR (KBr, cm^−1^): 3373, 2929, 1632, 1521, 1351. HRMS for (C_23_H_26_N_6_ [M+H]^+^). Calcd: 387.2297. Found: 387.2261.

*N^2^,N^6^-Dibenzyl-9-isobutyl-9H-purine-2,6-diamine* (**4c**): White solid, yield (74%), mp 111–113 °C. ^1^H-NMR (CDCl_3_) δ 7.42–7.24 (m, 3H), 6.02 (br, 1H), 5.86 (br, 1H), 4.62 (d, *J* = 6.0 Hz, 1H), 4.42 (d, *J* = 5.7 Hz, 1H), 3.79 (d, *J* = 7.2 Hz, 1H), 2.18 (m, 1H), 0.90 (d, *J* = 6.7 Hz, 1H). ^13^C-NMR (CDCl_3_) δ 160.01, 155.26, 140.83, 139.64, 138.67, 137.85, 129.12, 128.91, 128.81, 128.74, 128.58, 128.27, 128.17, 128.08, 127.95, 127.58, 127.23, 114.72, 51.08, 46.45, 44.17, 29.27, 20.41 (2C). IR (KBr, cm^−1^): 3362, 2922, 1630, 1522, 1353. HRMS for (C_23_H_26_N_6_ [M+H]^+^). Calcd: 387.2297. Found: 387.2260.

*N^2^,N^6^-Dibenzyl-9-pentyl-9H-purine-2,6-diamine* (**4d**): White solid, yield (85%), mp 83–86 °C. ^1^H-NMR (CDCl_3_) δ 7.39–7.22 (m, 11H), 6.14 (br, 1H), 5.89 (br, 1H), 4.62 (d, *J* = 5.9 Hz, 2H), 4.49–4.31 (m, 2H), 3.97 (t, *J* = 7.0 Hz, 2H), 1.88–1.69 (m, 2H), 1.40–1.17 (m, 2H), 0.88 (t, *J* = 7.1 Hz, 3H). ^13^C- NMR (CDCl_3_) δ 159.98, 155.26, 140.82, 139.68, 138.70, 137.40, 129.12, 128.90, 128.75 (2C), 128.27, 128.15, 128.06 (2C), 127.95, 127.56, 127.24, 46.43, 44.17, 43.64, 29.94, 29.16, 22.56, 14.30. IR (KBr, cm^−1^): 3269, 2956, 1632, 1528, 1351. HRMS for (C_24_H_28_N_6_ [M+H]^+^). Calcd: 401.2454. Found: 401.2418.

*N^2^,N^6^-Dibenzyl-9-isopentyl-9H-purine-2,6-diamine* (**4e**): White solid, yield (43%), mp 69–70 °C. ^1^H-NMR (CDCl_3_) δ 7.37–7.19 (m, 11H), 6.41 (br, 1H), 5.93 (br, 1H), 4.84–4.66 (m, 2H), 4.61 (d, *J* = 6.0 Hz, 2H), 3.97–3.95 (m, 2H), 1.73–1.59 (m, 2H), 1.59–1.44 (m, 1H), 0.92 (d, *J* = 6.6 Hz, 6H). ^13^C-NMR (CDCl_3_) δ 160.02, 155.31, 151.62, 140.84, 140.31, 139.77, 137.20, 129.12, 129.06, 128.88, 128.75, 128.27, 128.11, 128.05, 127.94, 127.50, 127.23, 114.71, 46.40, 41.88, 39.15, 25.86, 22.71 (2C). IR (KBr, cm^−1^): 3267, 2955, 1630, 1526, 1352. HRMS for (C_24_H_28_N_6_ [M+H]^+^). Calcd: 401.2454. Found: 401.2417. 

*N^2^,N^6^-Dibenzyl-9-hexyl-9H-purine-2,6-diamine* (**4f**): White solid, yield (93%), mp 79–81 °C. ^1^H-NMR (CDCl_3_) δ 7.46–7.26 (m, 11H), 6.06 (br, 1H), 5.81 (br, 1H), 4.79 (br, 2H), 4.63 (s, 2H), 3.98 (s, 2H), 1.74 (br, 2H), 1.28 (br, 6H), 0.80 (br, 3H). ^13^C-NMR (CDCl_3_) δ 160.01, 155.28, 140.83, 139.68, 137.40, 129.14, 128.91 (2C), 128.76 (2C), 128.17 (2C), 128.07 (2C), 127.57, 127.25, 114.84, 46.44, 44.19, 43.68, 31.66, 30.21, 26.72, 22.88, 14.39. IR (KBr, cm^−1^): 3371, 2954, 1630, 1523, 1351. HRMS for (C_25_H_30_N_6_ [M+H]^+^). Calcd: 415.2610. Found: 415.2571.

*N^2^-Benzyl-9-isopropyl-N^6^-(4-methoxybenzyl)-9H-purine-2,6-diamine* (**4g**): White solid, yield (83%), mp 69–70 °C. ^1^H-NMR (CDCl_3_) δ 7.36 (d, *J* = 7.6 Hz, 2H), 7.34–7.21 (m, 6H), 6.80 (d, *J* = 8.2 Hz, 2H), 6.09 (br, 2H), 4.67–4.59 (m, 4H), 4.40 (br, 1H), 3.76 (s, 3H), 1.49 (d, *J* = 6.4 Hz, 6H). ^13^C-NMR (CDCl_3_) δ 159.40, 158.78, 154.83, 140.48, 138.32, 134.47, 131.42, 129.06, 128.71 (2C), 128.36, 127.86, 127.65 (2C), 127.54, 127.51, 126.83, 113.89, 55.27, 46.03, 43.73, 22.55 (2C). IR (KBr, cm^−1^): 3291, 2927, 1628, 1541, 1354, 1249. HRMS for (C_23_H_26_N_6_O [M+H]^+^). Calcd: 403.2246. Found: 403.2231.

*N^2^-Benzyl-9-butyl-N^6^-(4-methoxybenzyl)-9H-purine-2,6-diamine* (**4h**): White solid, yield (91%), mp 92–94 °C. ^1^H-NMR (CDCl_3_) δ 7.36 (d, *J* = 7.4 Hz, 2H), 7.34–7.20 (m, 6H), 6.79 (d, *J* = 7.6 Hz, 2H), 6.06 (br, 2H), 4.64 (t, *J* = 8.4 Hz, 4H), 4.40 (d, *J* = 2.8 Hz, 2H), 3.76 (s, 3H), 1.81–1.72 (m, 2H), 1.34–1.26 (m, 2H), 0.92 (t, *J* = 7.4 Hz, 3H). ^13^C-NMR (CDCl_3_) δ 160.02, 159.22, 155.25, 140.88, 137.33, 131.80, 129.47, 129.09, 128.74 (2C), 128.24, 128.05 (2C), 127.90, 127.89, 127.22, 114.30, 55.66, 46.42, 44.12, 43.32, 32.30, 20.22, 13.94. IR (KBr, cm^−1^): 3290, 2928, 1632, 1519, 1351, 1251. HRMS for (C_24_H_28_N_6_O [M+H]^+^). Calcd: 417.2403. Found: 417.2367.

*N^2^-Benzyl-9-isobutyl-N^6^-(4-methoxybenzyl)-9H-purine-2,6-diamine* (**4i**): White solid, yield (85%), mp 110–112 °C. ^1^H-NMR (CDCl_3_) δ 7.35 (d, *J* = 7.3 Hz, 2H), 7.32–7.17 (m, 6H), 6.79 (d, *J* = 7.3 Hz, 2H), 6.22 (br, 2H), 4.71–4.52 (m, 4H), 4.41 (br, 2H), 3.76 (s, 3H), 2.14 (br, 1H), 0.89 (d, *J* = 6.6 Hz, 6H). ^13^C-NMR (CDCl_3_) δ 160.05, 159.19, 155.27, 140.91, 138.80, 137.81, 131.84, 129.46, 129.07, 128.73 (2C), 128.22, 128.07 (2C), 127.89, 127.86, 127.21, 114.29, 55.65, 46.43, 44.08, 29.25, 20.40 (2C). IR (KBr, cm^−1^): 3291, 2926, 1619, 1512, 1354, 1253. HRMS for (C_24_H_28_N_6_O [M+H]^+^). Calcd: 417.2403. Found: 417.2364.

*N^2^-Benzyl-N^6^-(4-methoxybenzyl)-9-pentyl-9H-purine-2,6-diamine* (**4j**): White solid, yield (79%), mp 86–88 °C. ^1^H-NMR (CDCl_3_) δ 7.36 (d, *J* = 7.2 Hz, 2H), 7.28–7.21 (m, 6H), 6.80 (d, *J* = 8.3 Hz, 2H), 6.08 (br, 2H), 4.63 (d, *J* = 5.8 Hz, 2H), 4.40 (d, *J* = 4.0 Hz, 2H), 3.95 (d, *J* = 6.7 Hz, 2H), 3.76 (s, 3H), 1.84–1.75 (m, 2H), 1.35–1.24 (m, 4H), 0.87 (t, *J* = 6.9 Hz, 3H). ^13^C-NMR (CDCl_3_) δ 160.01, 159.25, 155.23, 140.87, 138.78, 137.34, 131.78, 129.47, 129.09, 128.74 (2C), 128.23, 128.04 (2C), 127.89, 127.22, 114.33, 55.66, 46.42, 44.12, 43.63, 29.92, 29.15, 22.54, 14.28. IR (KBr, cm^−1^): 3293, 2925, 1629, 1527, 1355, 1252. HRMS for (C_25_H_30_N_6_O [M+H]^+^). Calcd: 431.2559. Found: 431.2518.

*N^2^-Benzyl-9-isopentyl-N^6^-(4-methoxybenzyl)-9H-purine-2,6-diamine* (**4k**): White solid, yield (74%), mp 86–90 °C. ^1^H-NMR (CDCl_3_) δ 7.39–7.22 (m, 8H), 6.80 (d, *J* = 8.4 Hz, 2H), 6.01 (br, 2H), 4.63 (d, *J* = 5.9 Hz, 2H), 4.40 (d, *J* = 5.6 Hz, 2H), 3.99 (t, *J* = 7.3 Hz, 2H), 3.76 (s, 3H), 1.68 (q, *J* = 7.0 Hz, 2H), 1.60–1.50 (m, 1H), 0.93 (d, *J* = 6.5 Hz, 6H). ^13^C-NMR (CDCl_3_) δ 159.59, 158.80, 154.80, 140.44, 138.29, 136.77, 131.32, 129.08, 128.70 (2C), 128.36, 127.85 (2C), 127.64, 127.52, 126.84, 113.90, 55.27, 45.99, 43.73, 41.48, 38.75, 25.46, 22.31 (2C). IR (KBr, cm^−1^): 3290, 2926, 1620, 1526, 1351, 1252. HRMS for (C_25_H_30_N_6_O [M+H]^+^). Calcd: 431.2559. Found: 431.2521.

*N^2^-Benzyl-9-hexyl-N^6^-(4-methoxybenzyl)-9H-purine-2,6-diamine* (**4l**): White solid, yield (98%), mp 108–111 °C. ^1^H-NMR (CDCl_3_) δ 7.35–7.21 (m, 8H), 6.79 (d, *J* = 8.6 Hz, 2H), 6.08 (br, 2H), 4.66–4.62 (m, 4H), 3.95 (t, *J* = 7.2 Hz, 2H), 3.76 (s, 3H), 1.83–1.73 (m, 2H), 1.27 (br, 6H), 0.86 (t, *J* = 6.7 Hz, 3H). ^13^C-NMR (CDCl_3_) δ 160.02, 159.22, 155.24, 140.88, 138.74, 137.33, 131.78, 129.47, 129.10, 128.74 (2C), 128.25, 128.04 (2C), 127.91, 127.22, 114.30, 55.66, 46.42, 44.14, 43.65, 31.65, 30.20, 26.71, 22.88, 14.38. IR (KBr, cm^−1^): 3363, 2927, 1632, 1524, 1351, 1242. HRMS for (C_26_H_32_N_6_O [M+H]^+^). Calcd: 445.2716. Found: 445.2675.

### 3.5. Anticancer Activity

#### 3.5.1. Cell Lines Culture

Cancer cell lines were grown at 37 °C in humidified atmosphere and 5% CO_2_. H1975, HL60, HTC116 and Vero cells were grown in RPMI 1640 culture medium supplemented with 10% fetal bovine serum and (100 UI/mL) penicillin-streptomicyn. HeLa cells were grown in EMEM culture medium, supplemented with 10% fetal bovine serum and (100 UI/mL) penicillin-streptomicyn.

#### 3.5.2. Cytotoxicity Study

Cytotoxicity assays were performed by using the MTT reduction method as described previously [23]. Briefly, cancer cell lines were plated in a flat-bottom 96-wells plate at 10,000 cells per well density. Then, the cells were incubated with synthetized compounds at different concentrations (from 0.1 to 50 µM) in 200 µL of 10% fetal bovine serum-RPMI or EMEM culture medium at 37 °C for 72 h. Ten µL of MTT was added at a final concentration of 0.5 mg/mL, incubated at 37 °C for 4 h, and then solubilized with 10% sodium dodecyl sulfate (SDS) in 0.1 mM HCl and incubated overnight at 37 °C. Formazan formation was measured at 570 nm in a multiwell reader (StatFax 4200, Awareness Technology, Inc. Palm City, FL, USA).

#### 3.5.3. Viability Assays with Propidium Iodide

Cell viability was analyzed by FACS as previously described [24,25,26]. In this assay, after setting the baseline to exclude cell debris, cells impermeable to propidium iodide (PI negative) are considered as viable. Two populations of PI-permeable dead cells are distinguished based on fluorescence intensity, corresponding to either hypodiploid apoptotic cells or necrotic cells with intact DNA. Here, H1975, HTC116, and HeLa cells were incubated with concentrations of 50 µM of each compound for 16 h at 37 °C. Cells were harvested and stained with 10 µg/mL of propidium iodide to determine cell viability. Samples containing roughly 1 × 10^4^ cells were evaluated by flow cytometry (FACScanto II; Becton Dickinson, Mountain View, CA, USA) and analyzed using the software program FCS Express v5.

#### 3.5.4. Statical Analysis

Values are expressed as means ± standard deviations from three independent experiments. IC_50_ values, (Compound concentration necessary to decrease at 50% the MTT reduction) and the two-way analysis of variance (ANOVA) test were performed when necessary with Prism GraphPad 6.0f software (GraphPad Software Inc., San Diego, CA, USA).

### 3.6. Pharmacophore Elucidation

The 3D-structure of the pharmacophore hypothesis was carried out using Molecular Operating Environment (MOE) software version 2013–2014, Chemical Computing Group Inc. (Montreal, QC, Canada).

## 4. Conclusions

A study of new 2,6,9-trisubstituted purines on four cancer cell lines and Vero cells, showed that certain chemical modifications on the purine moiety increased the cytotoxicity and selective effects. Some of these compounds were more potent than the reference drug etoposide and exhibited selectivity against three cancer cell lines in comparison with VERO cells, most notably **4f**, **4j** and **4k**. A preliminary analysis confirms the earlier conclusion that the purine core is a privilege scaffold, but attempts to reveal general structure-activity relationships using lipophilicity properties were unsuccessful, although pharmacophoric features have been recognized. Nevertheless, the main mechanism of action observed for the most active compounds in each cancer cell lines is the induction of apoptosis. These results showed that **4f**, **4j** and **4k** are promising leads for the development of new antitumor drugs.

## Supplementary Materials

Supplementary materials can be accessed at: http://www.mdpi.com/1420-3049/20/04/6808/s1.
