# Supplementary material for: Synthesis and Pharmacophore Modelling of 2,6,9-Trisubstituted Purine Derivatives and Their Potential Role as Apoptosis-Inducing Agents in Cancer Cell Lines
Source: Molecules. 2015 Apr 15;20(4):6808–26. doi: 10.3390/molecules20046808 (PMC6272238; doi:10.3390/molecules20046808)
Supplement: Supplementary file 1 [file molecules-20-06808-s001.pdf]

Supplementary Data

Table of Contents

HRMS for target compounds **4a-l**	2–13
^{1}H- and ^{13}C-NMR for selected compounds	14–16
HMBC spectra	17
Flow cytometry analysis on VERO cells	18–21
Flow cytometry analysis on HeLa cells	22–25
Flow cytometry analysis on H1975 cells	26–29
Flow cytometry analysis on HTC116 cells	30–33

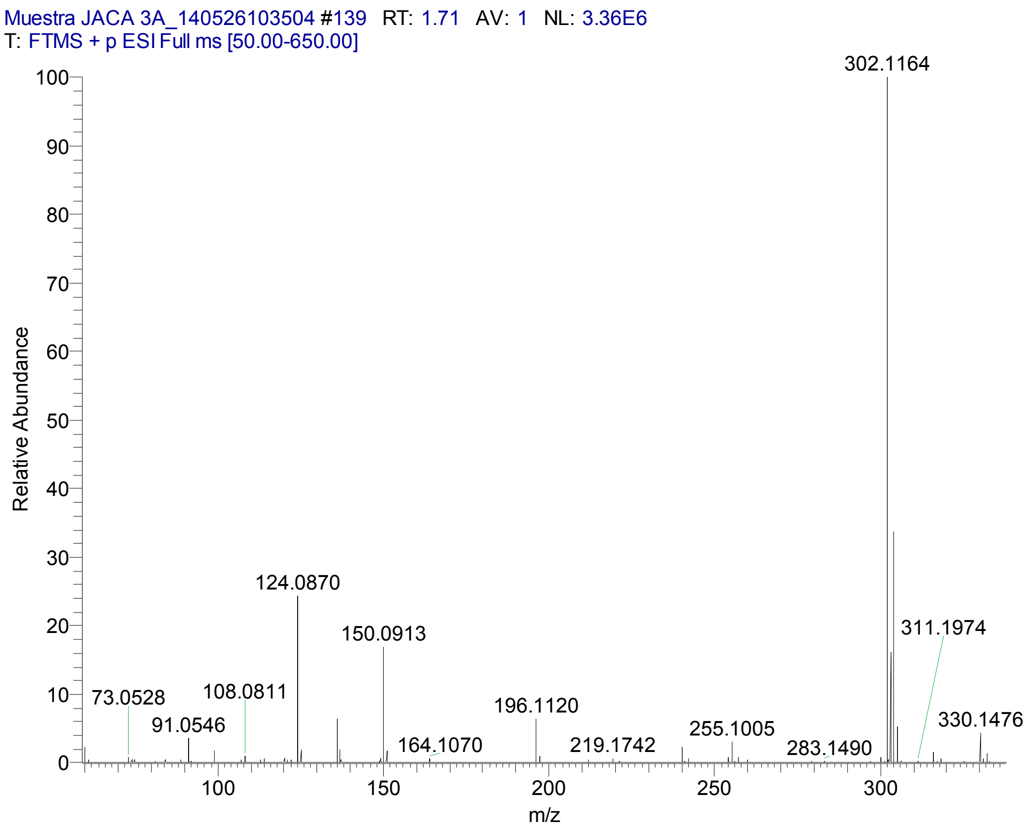

Figure S1. HRMS for compound **3a**.

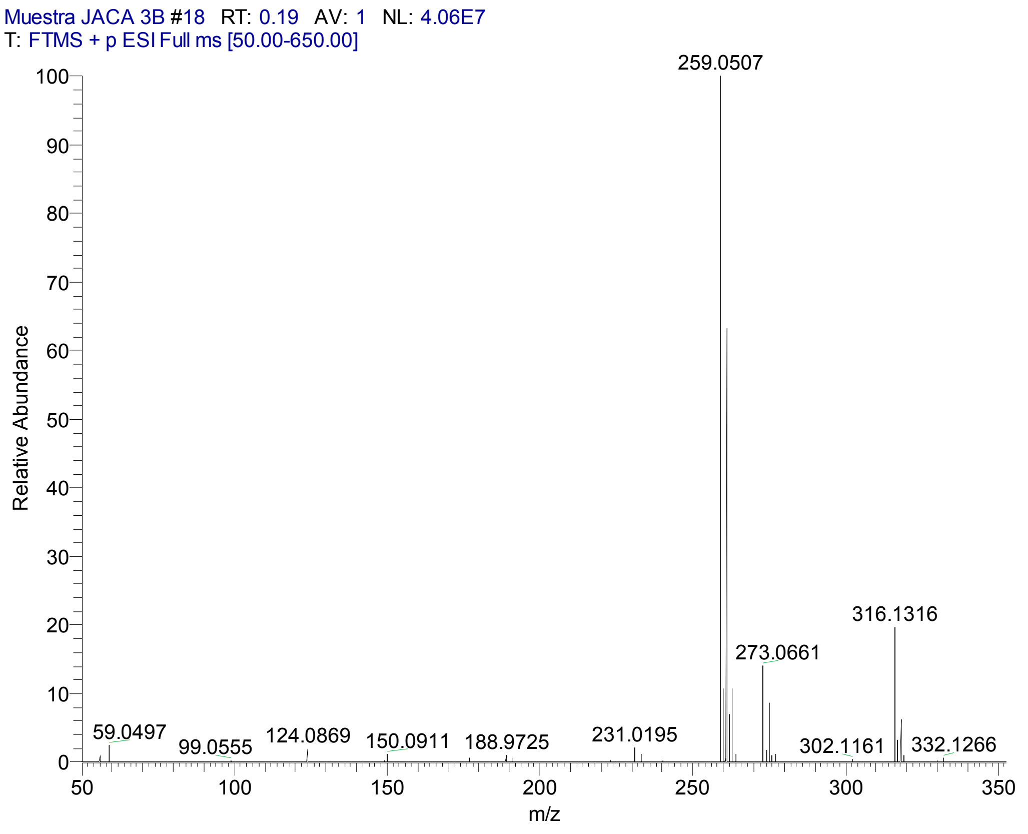

Figure S2. HRMS for compound **3b**.

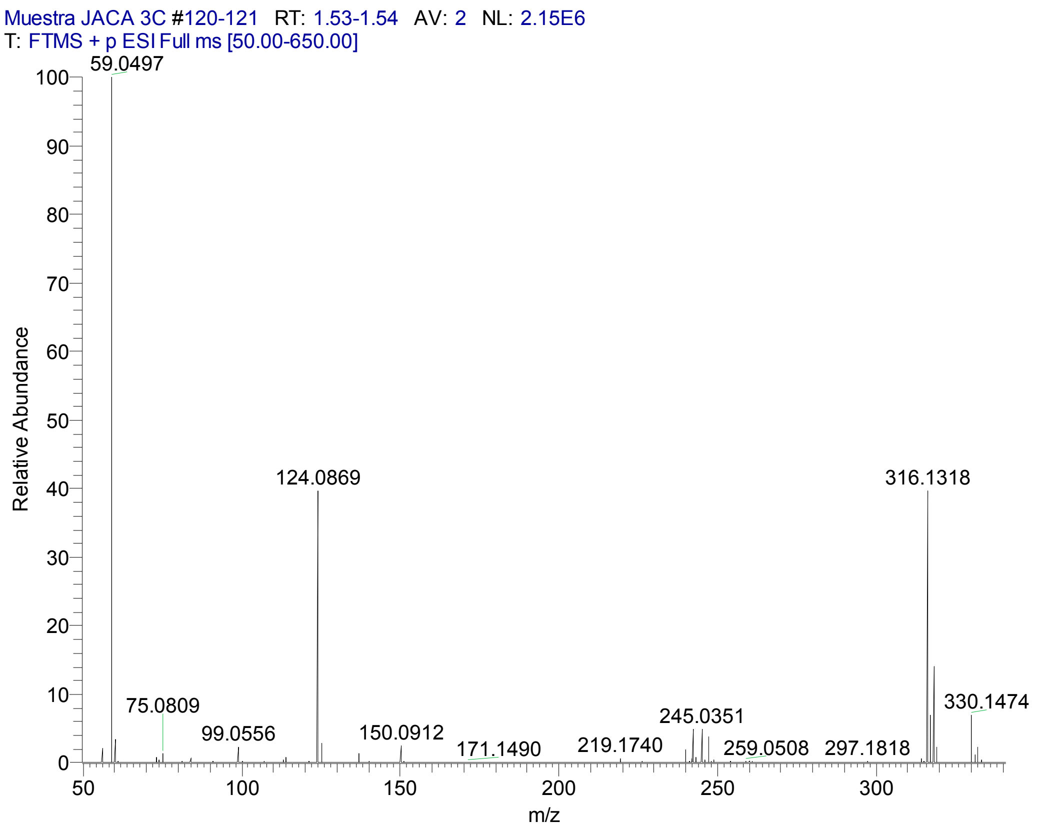

Figure S3. HRMS for compound **3c**.

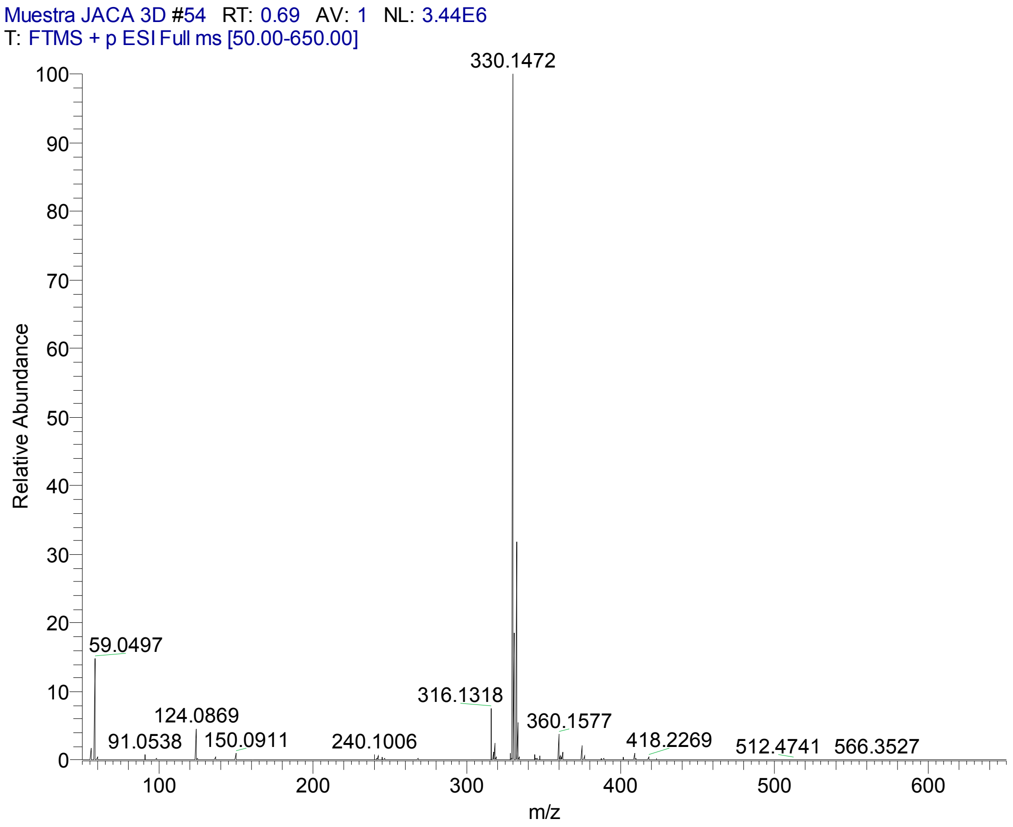

Figure S4. HRMS for compound **3d**.

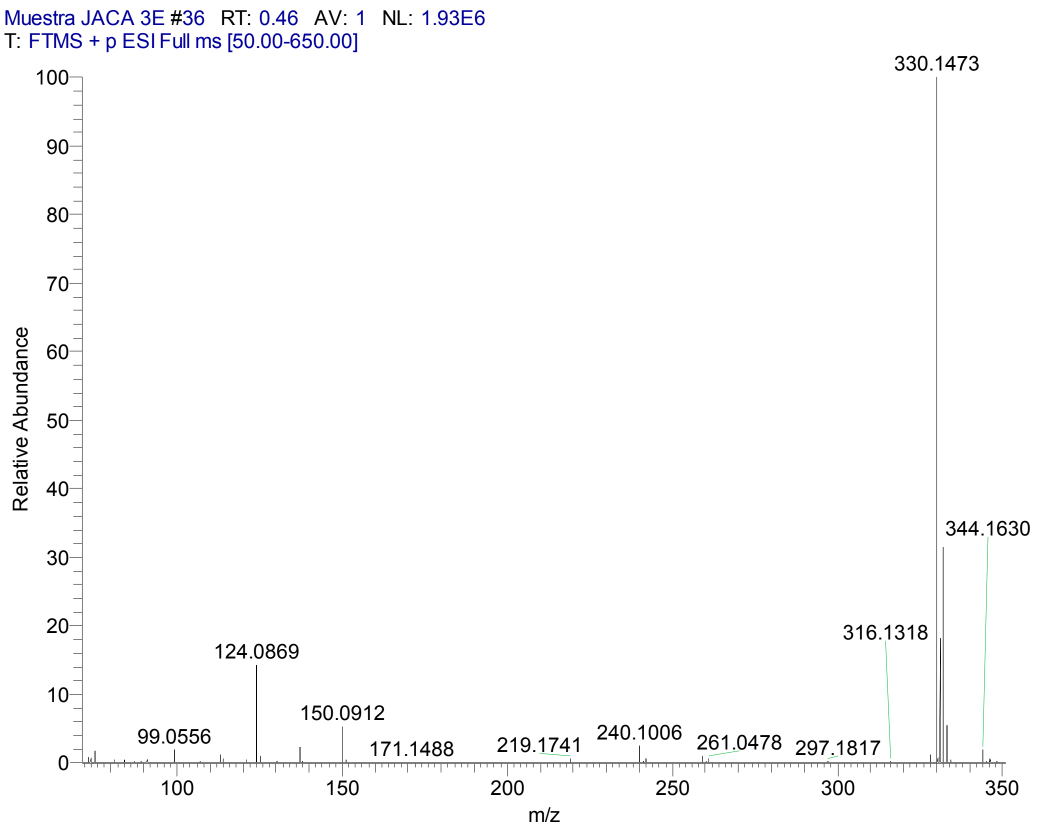

Figure S5. HRMS for compound **3e**.

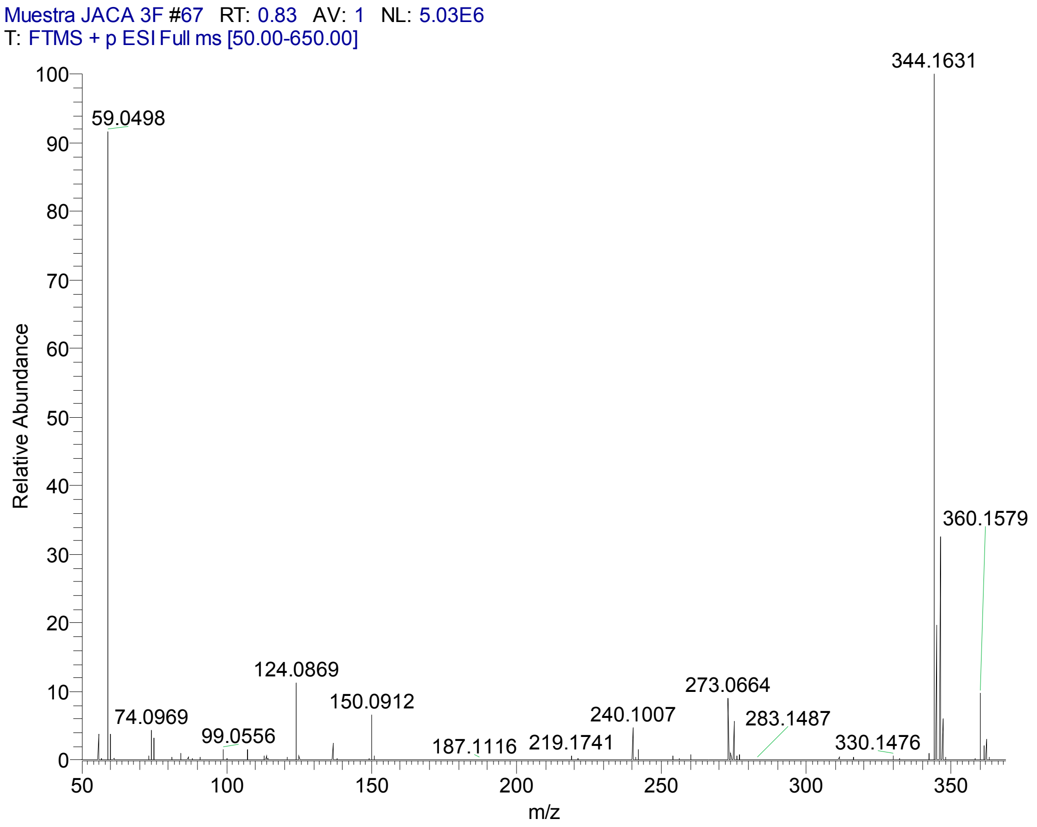

Figure S6. HRMS for compound **3f**.

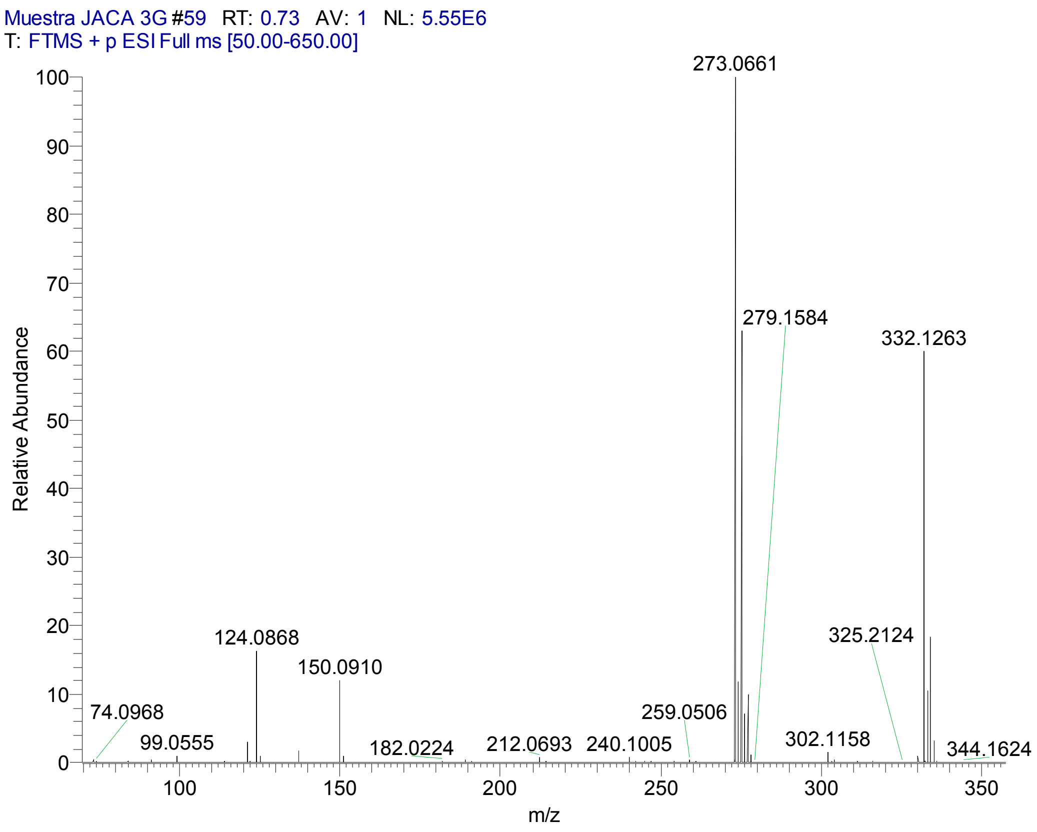

Figure S7. HRMS for compound **3g**.

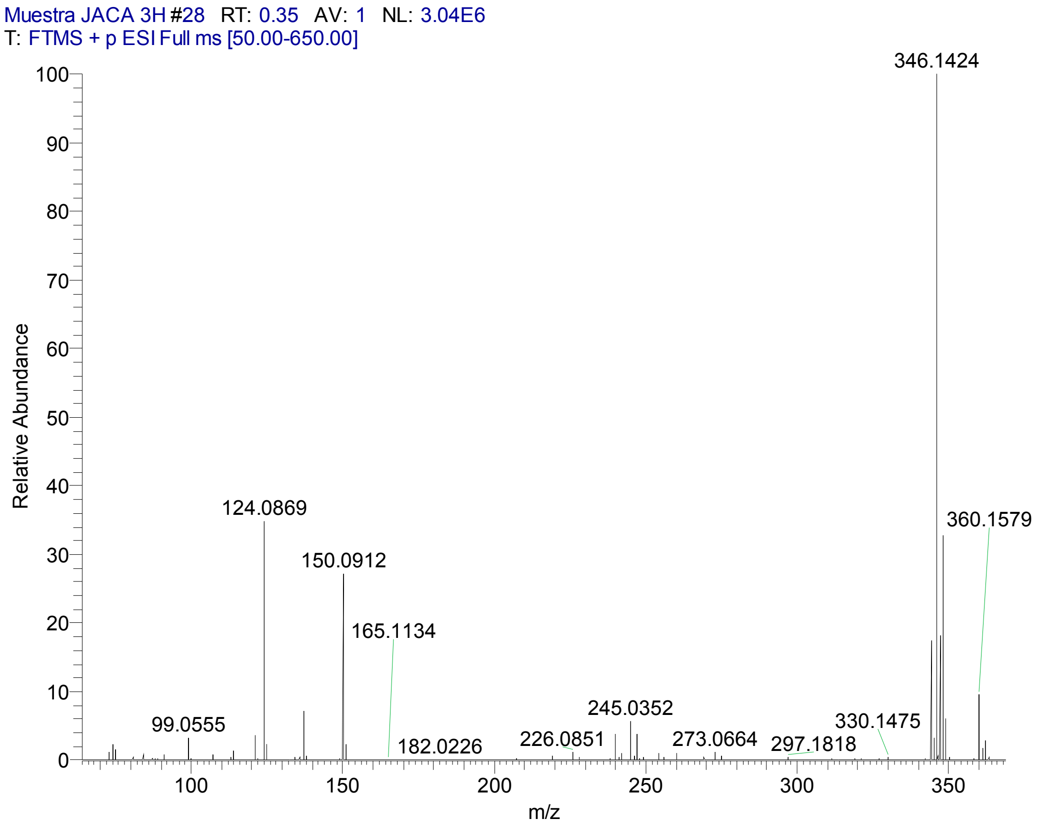

Figure S8. HRMS for compound **3h**.

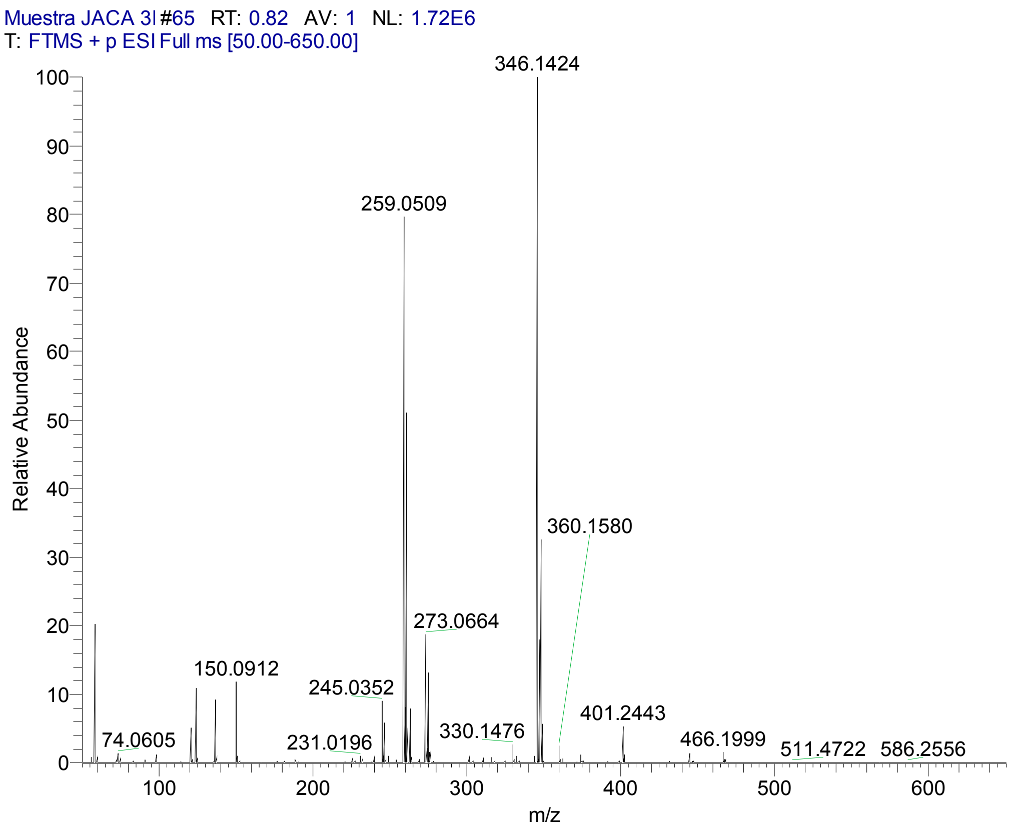

Figure S9. HRMS for compound **3i**.

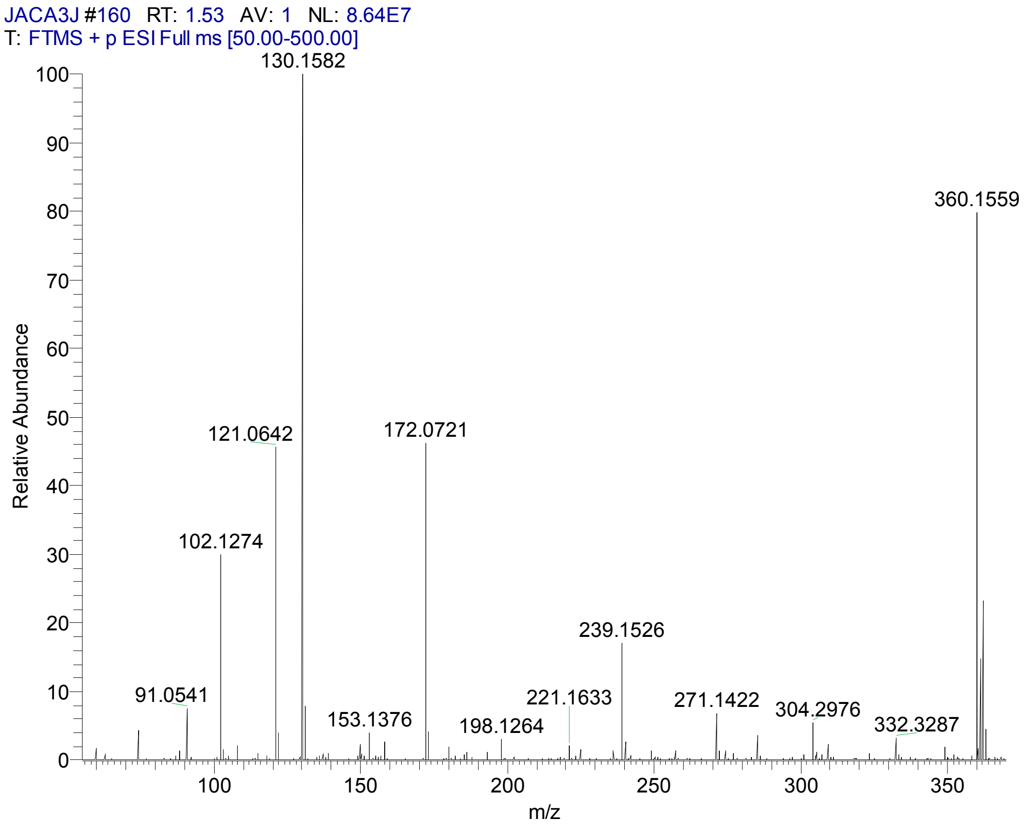

Figure S10. HRMS for compound **3j**.

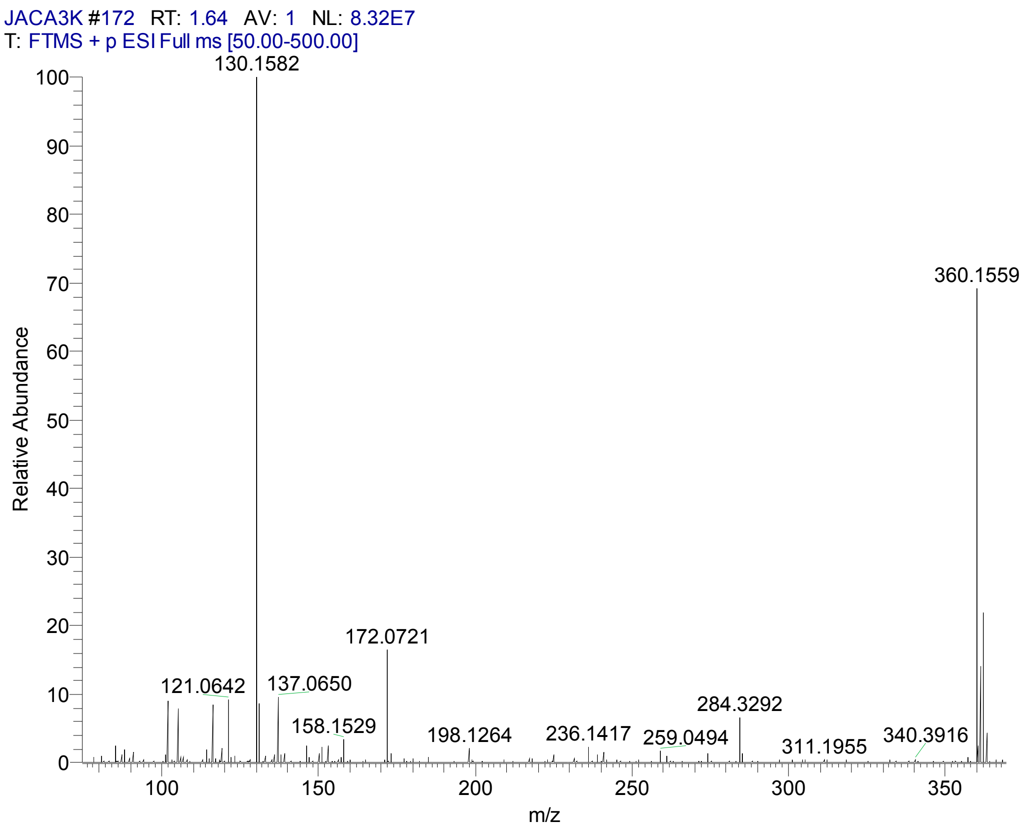

Figure S11. HRMS for compound **3k**.

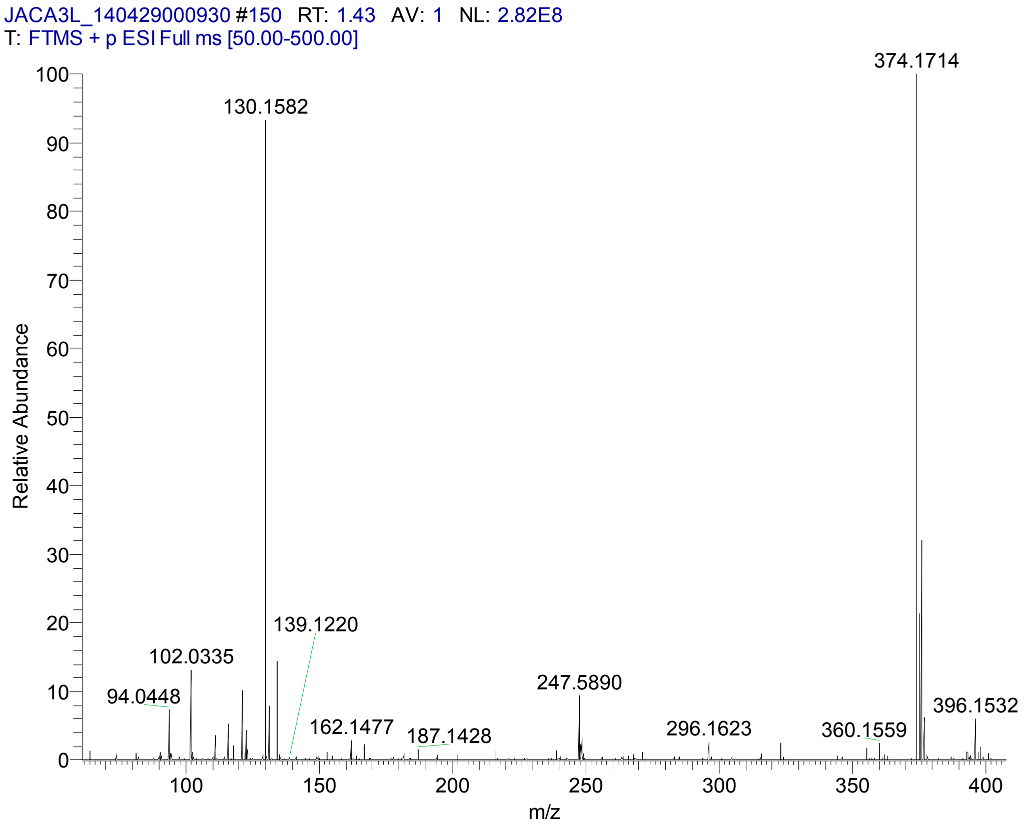

Figure S12. HRMS for compound **3l**.

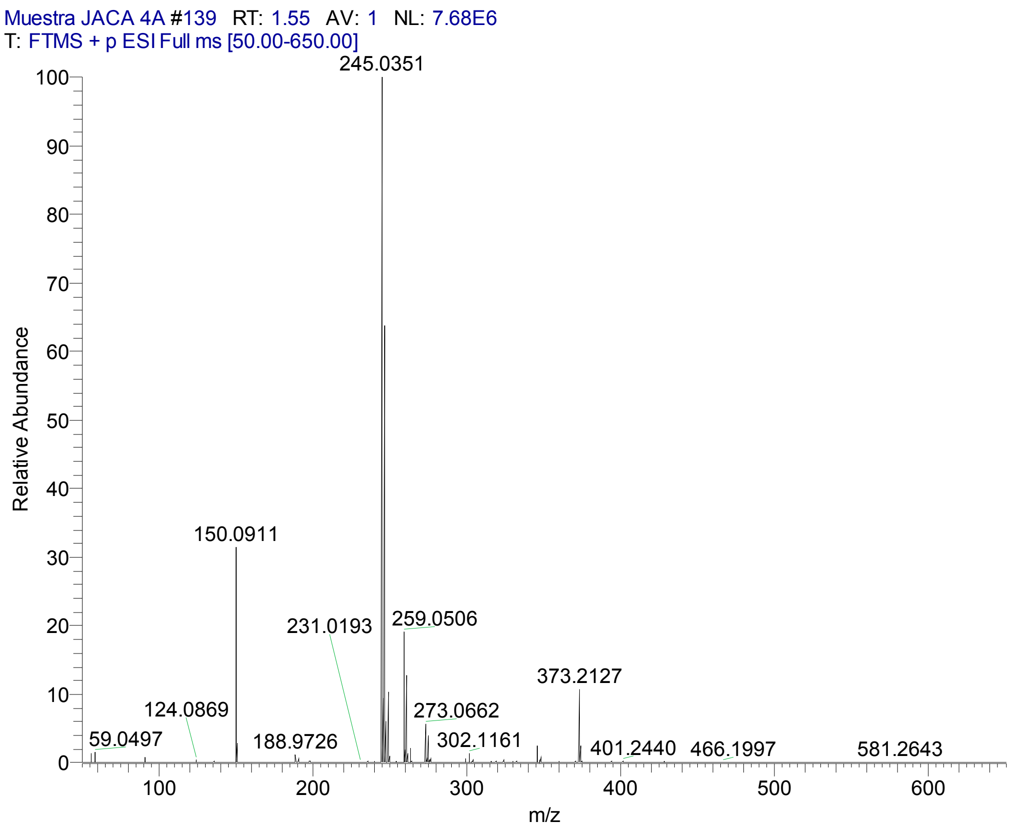

Figure S13. HRMS for compound **4a**.

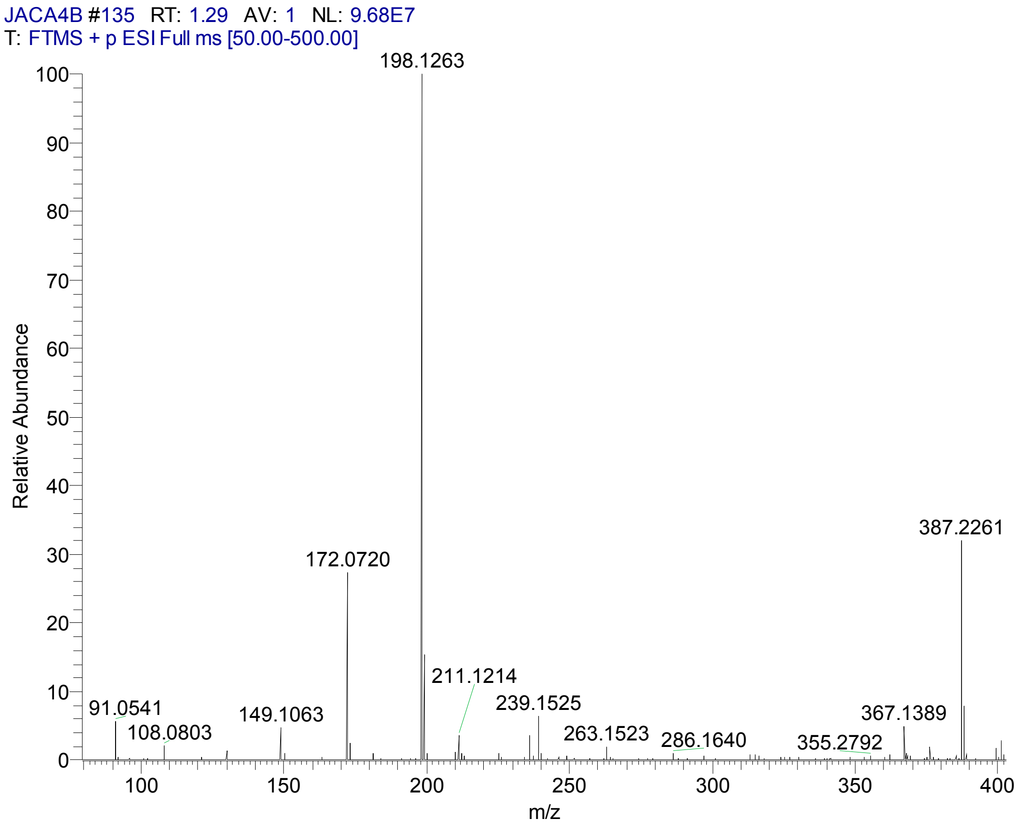

Figure S13. HRMS for compound **4b**.

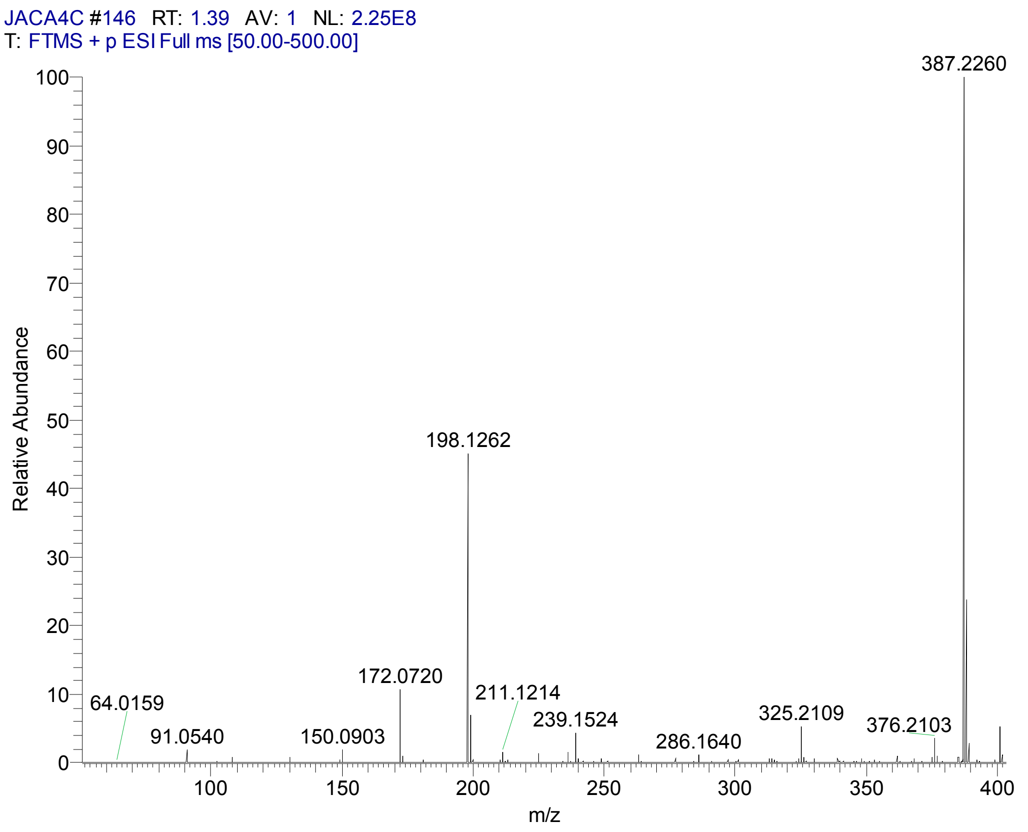

Figure S15. HRMS for compound **4c**.

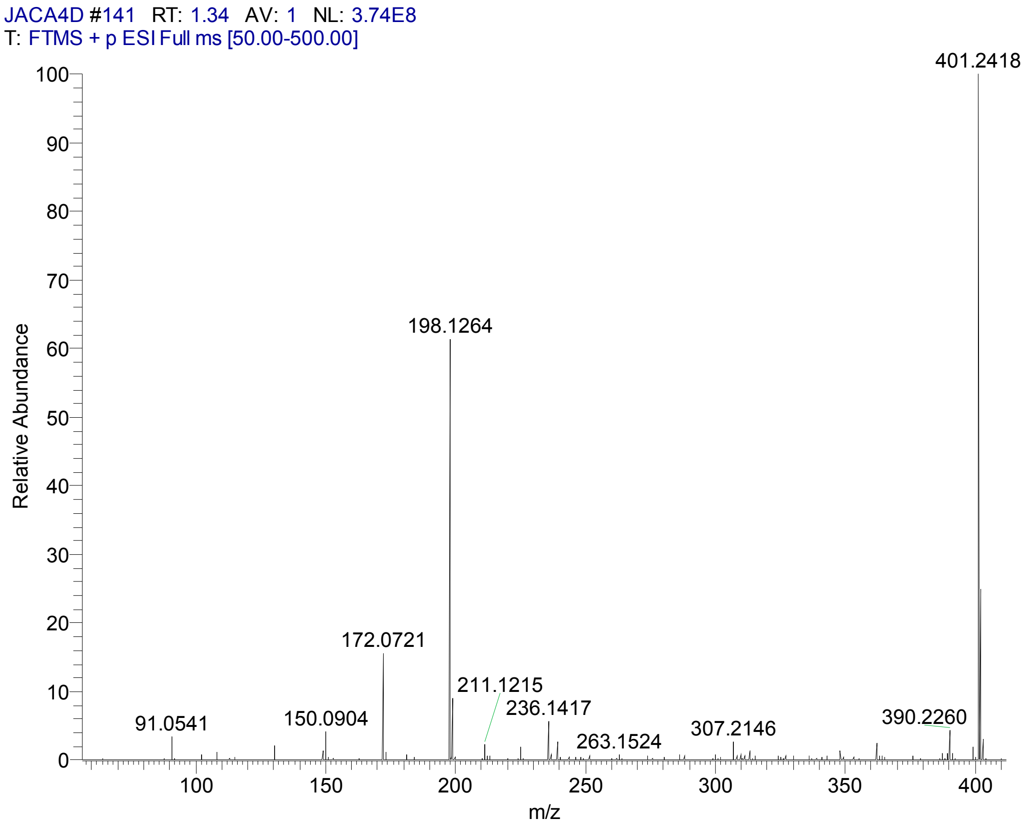

Figure S16. HRMS for compound **4d**.

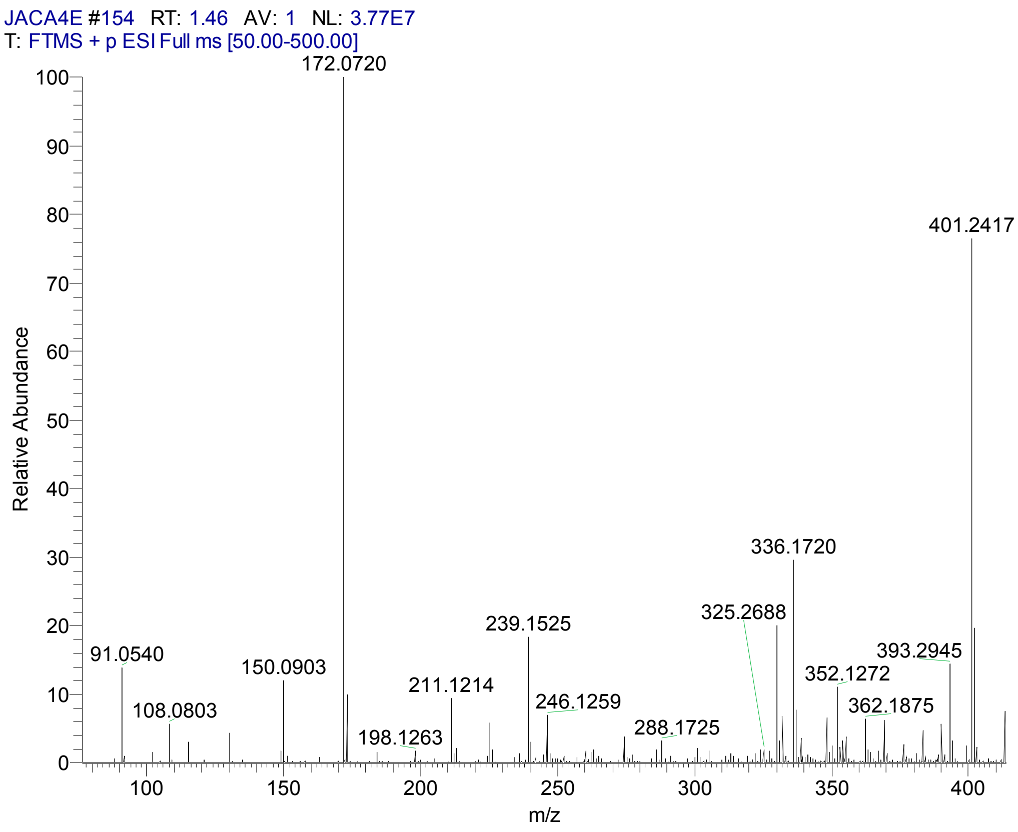

Figure S17. HRMS for compound **4e**.

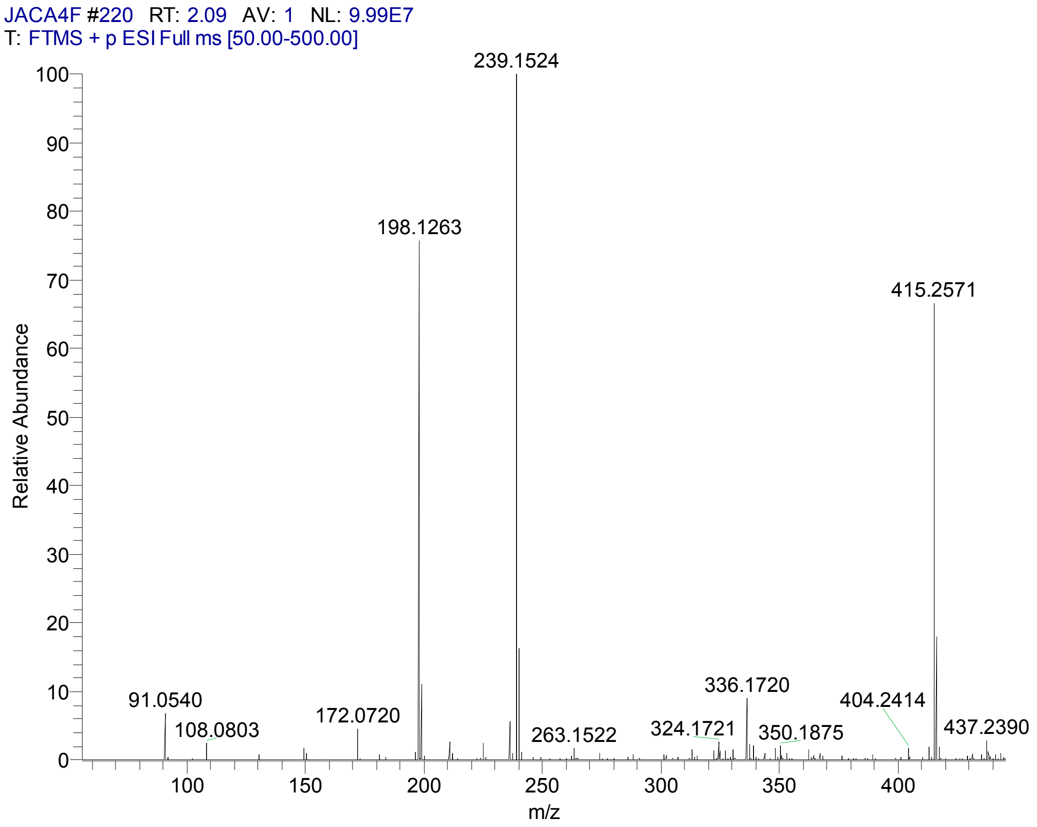

Figure S18. HRMS for compound **4f**.

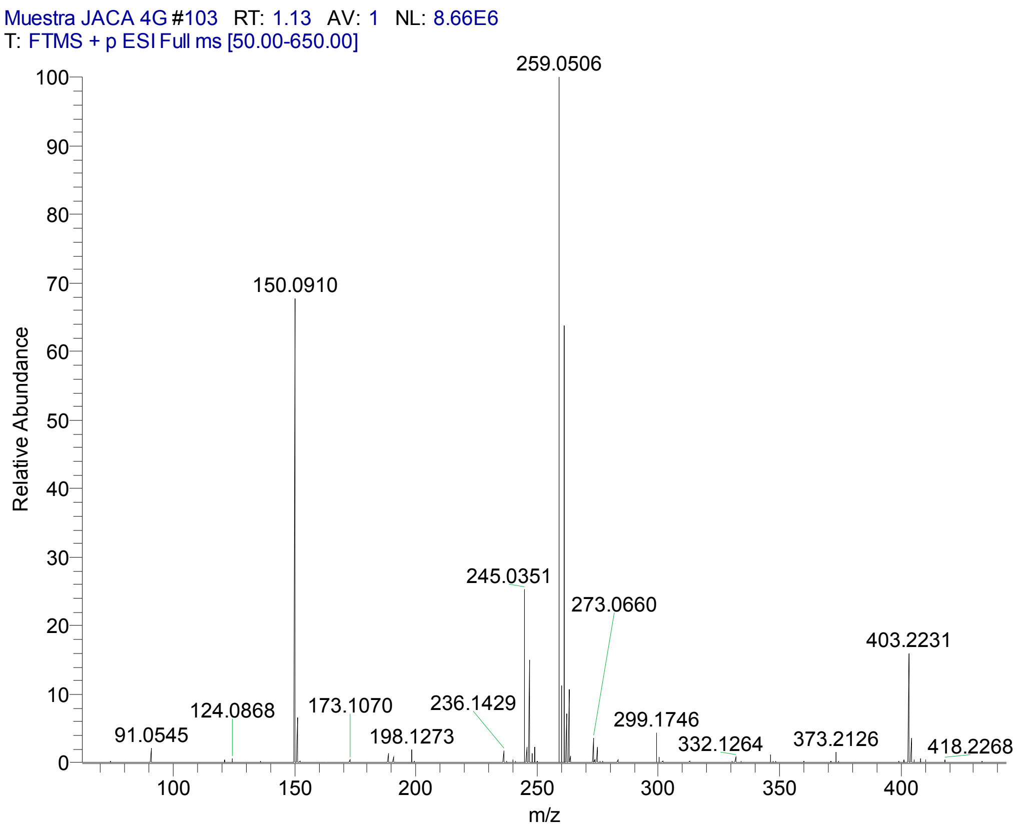

Figure S19. HRMS for compound **4g**.

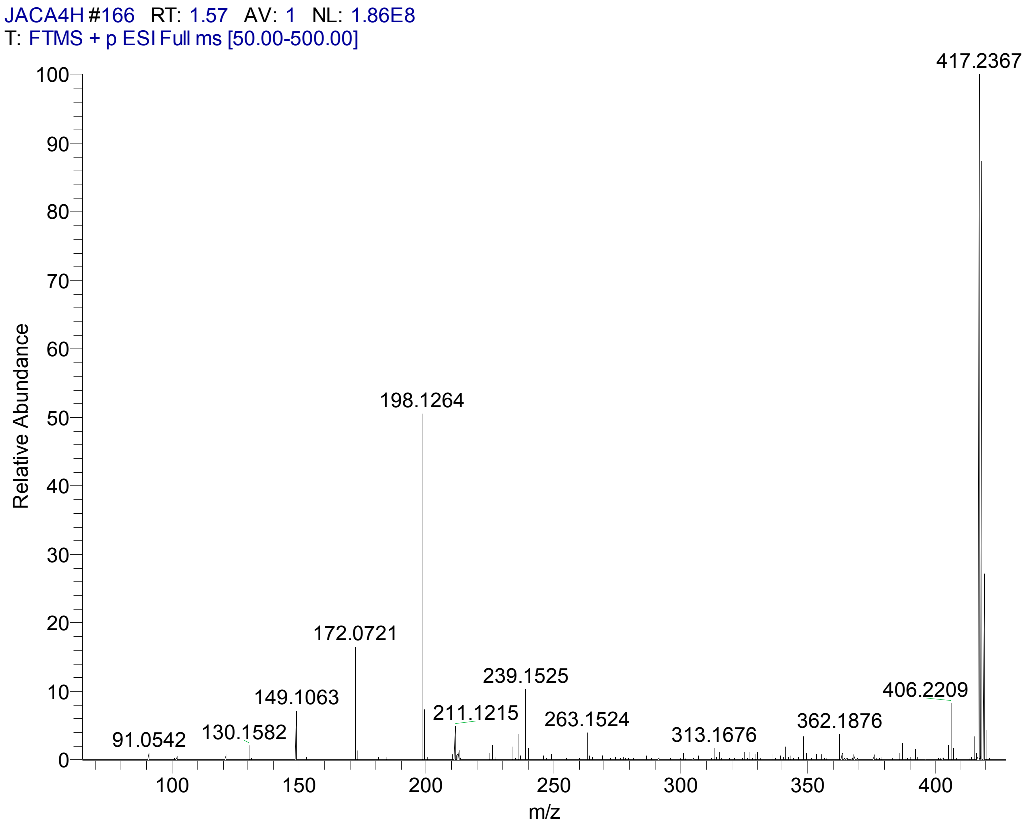

Figure S10. HRMS for compound **4h**.

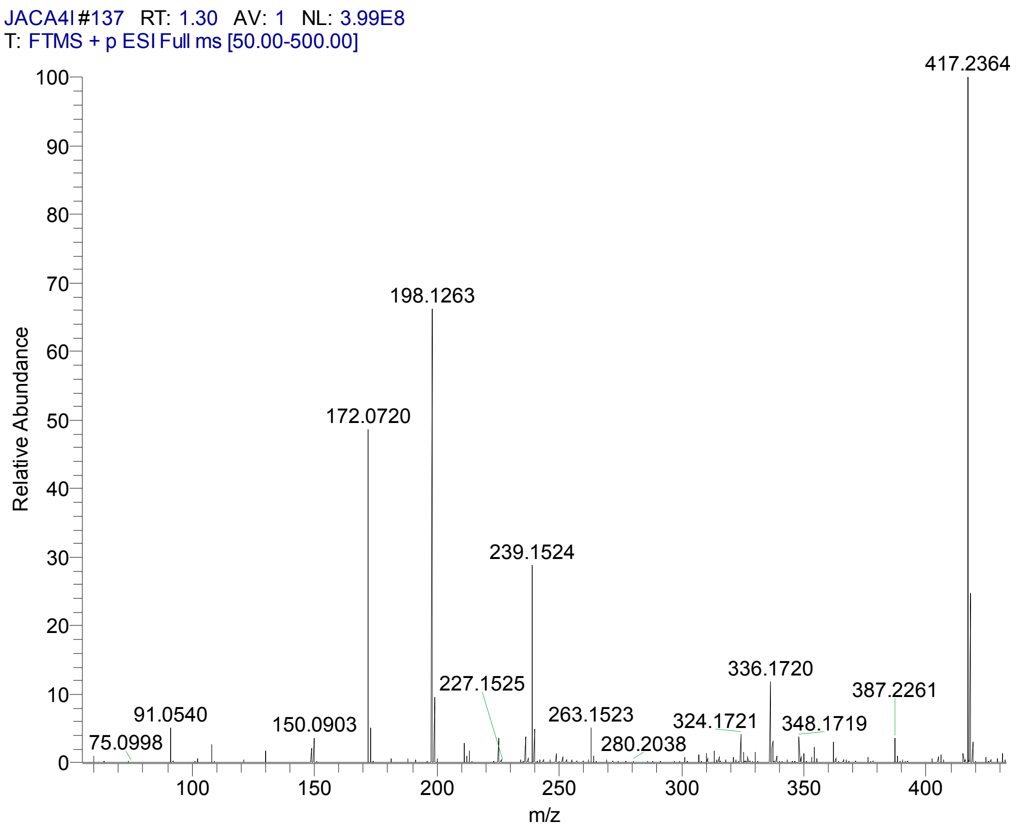

Figure S21. HRMS for compound **4i**.

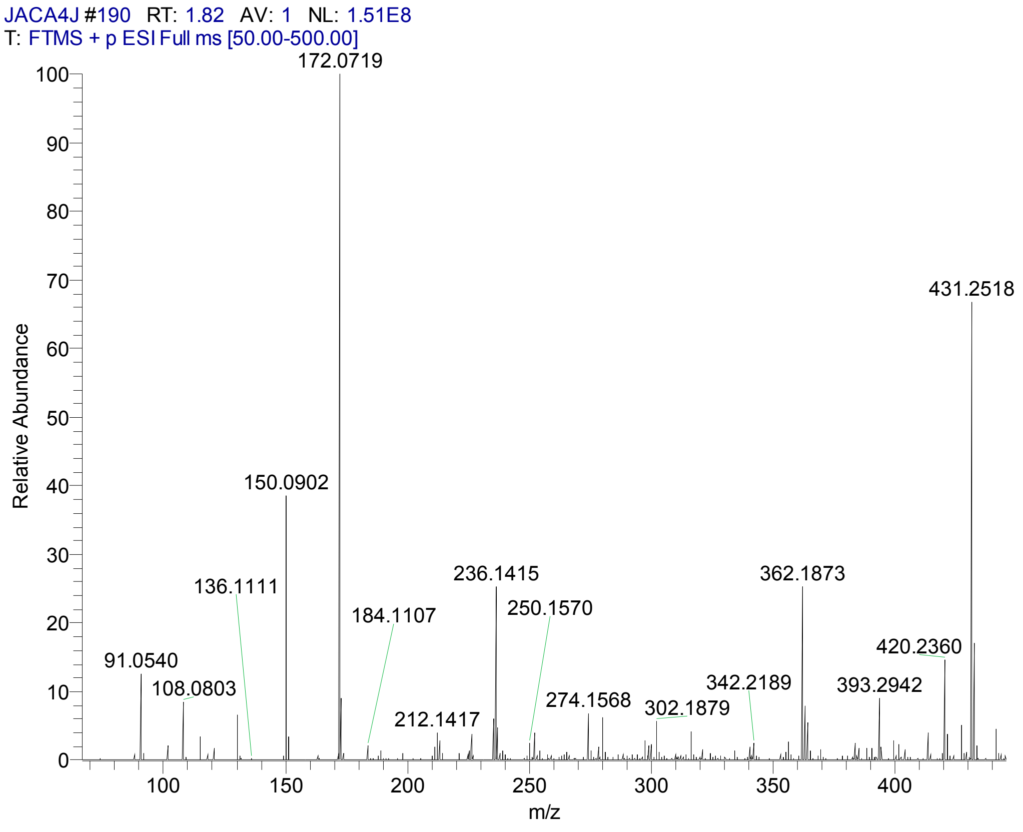

Figure S22. HRMS for compound **4j**.

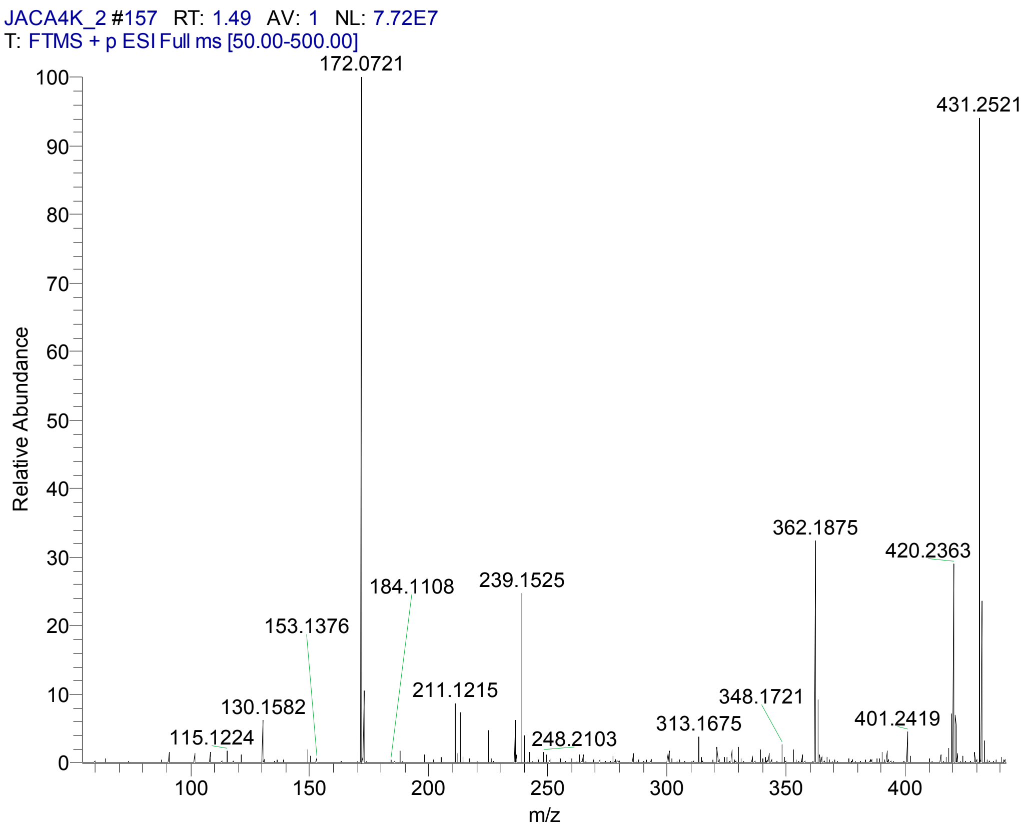

Figure S23. HRMS for compound **4k**.

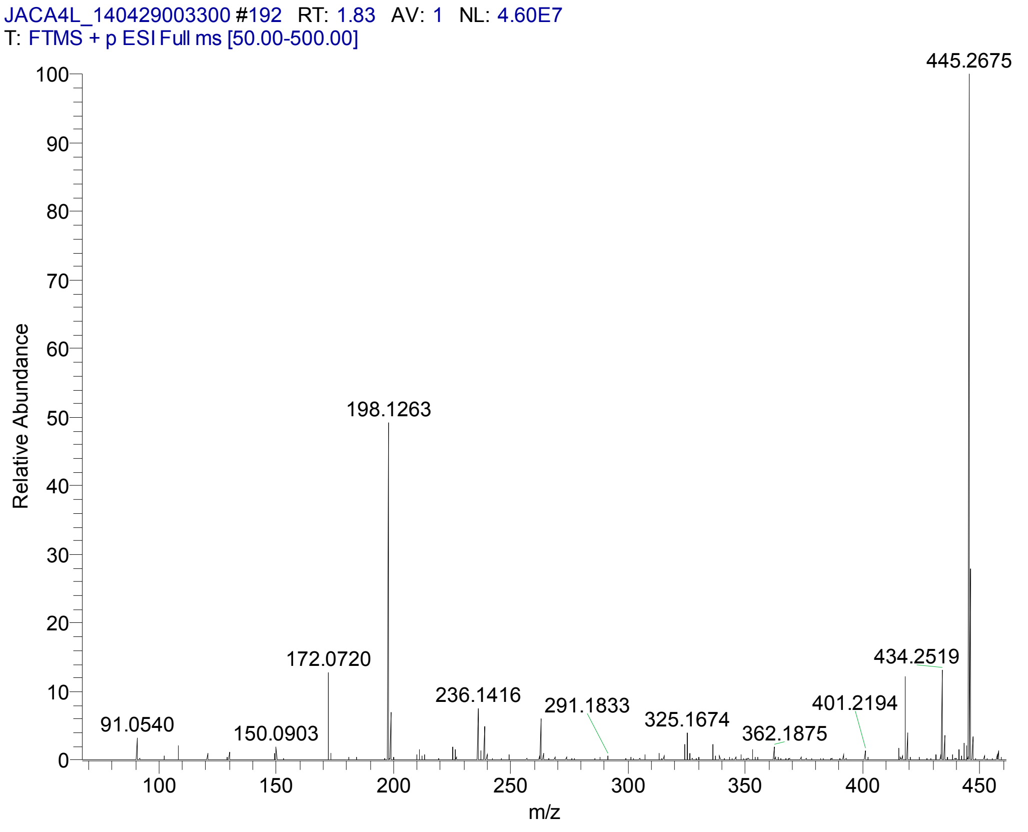

Figure S24. HRMS for compound **4l**.

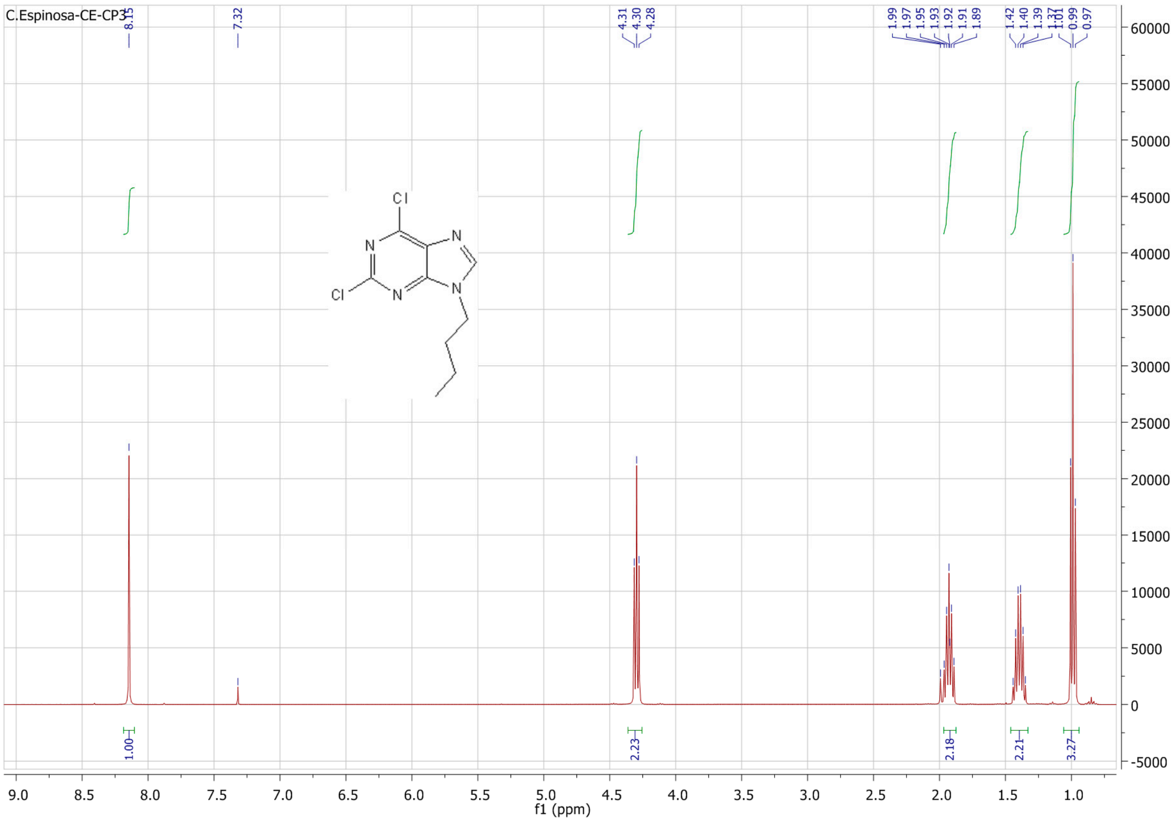

Figure S25. ^{1}H-NMR for compound **2b**.

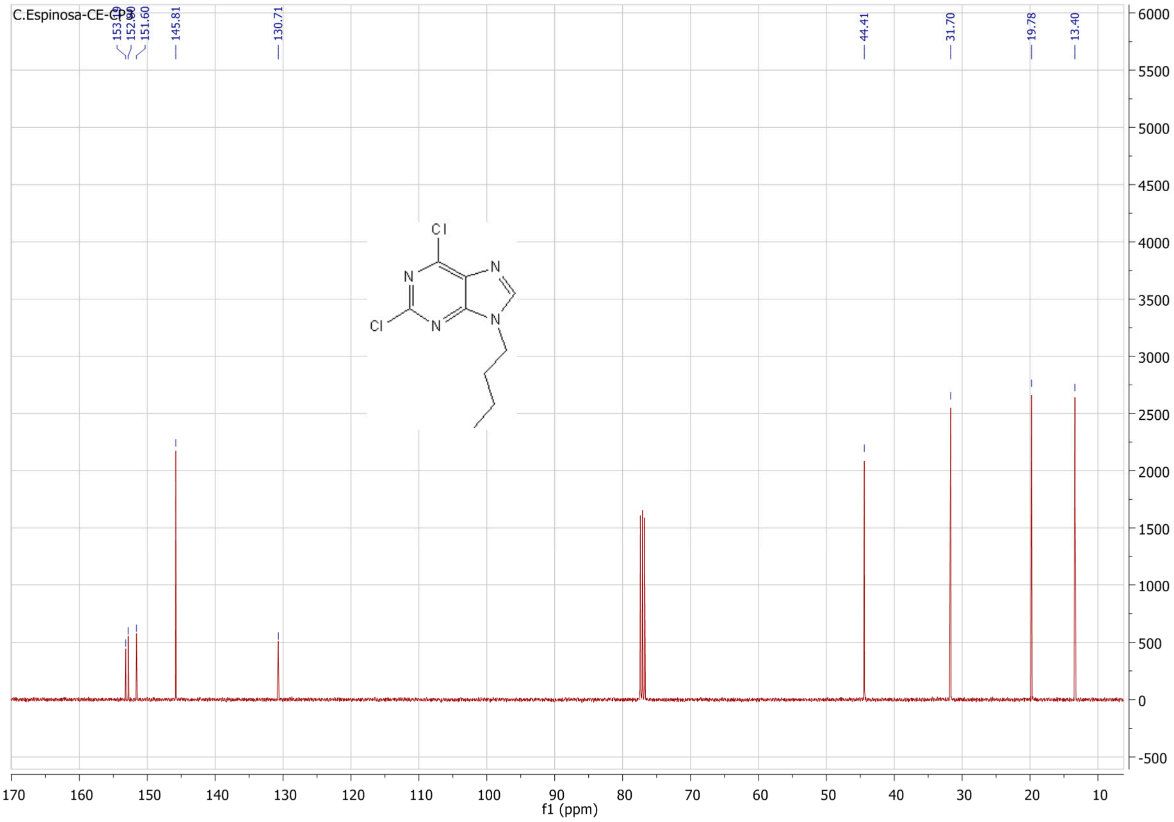

Figure S26. ^{13}C-NMR for compound **2b**.

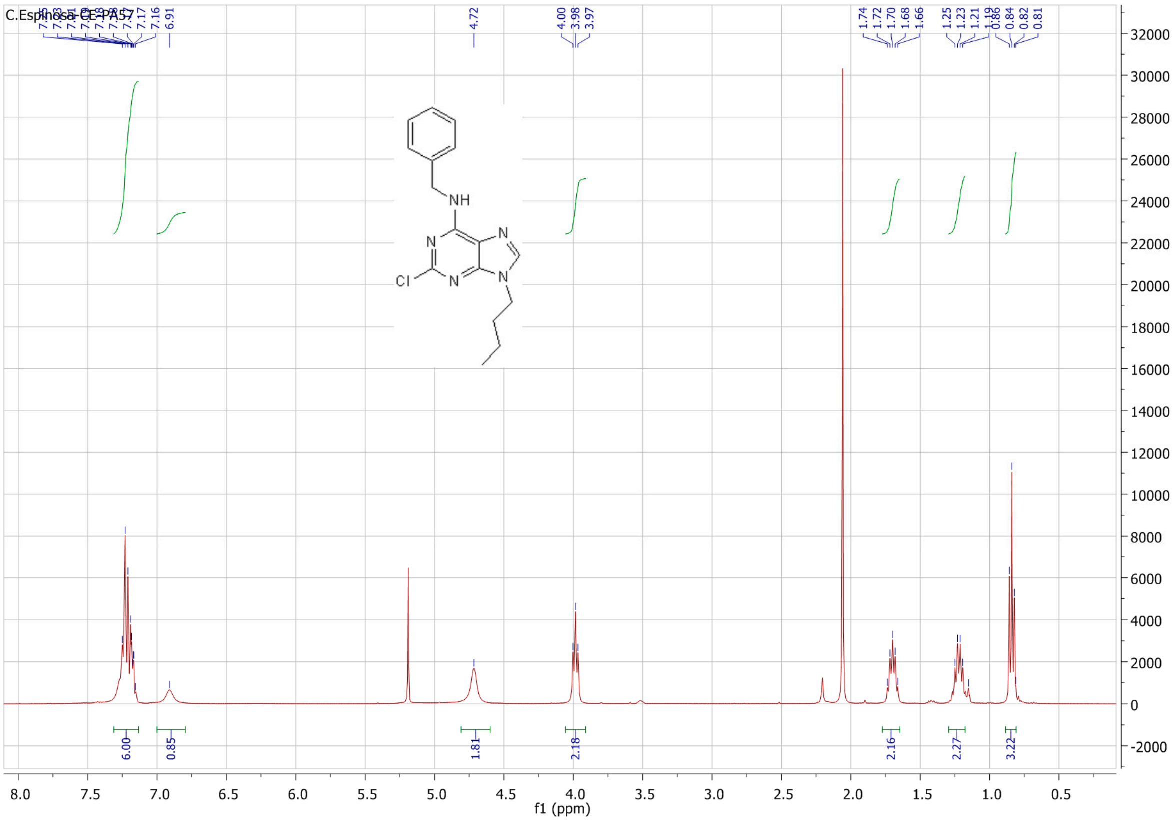

Figure S27. ¹H-NMR for compound **3b**.

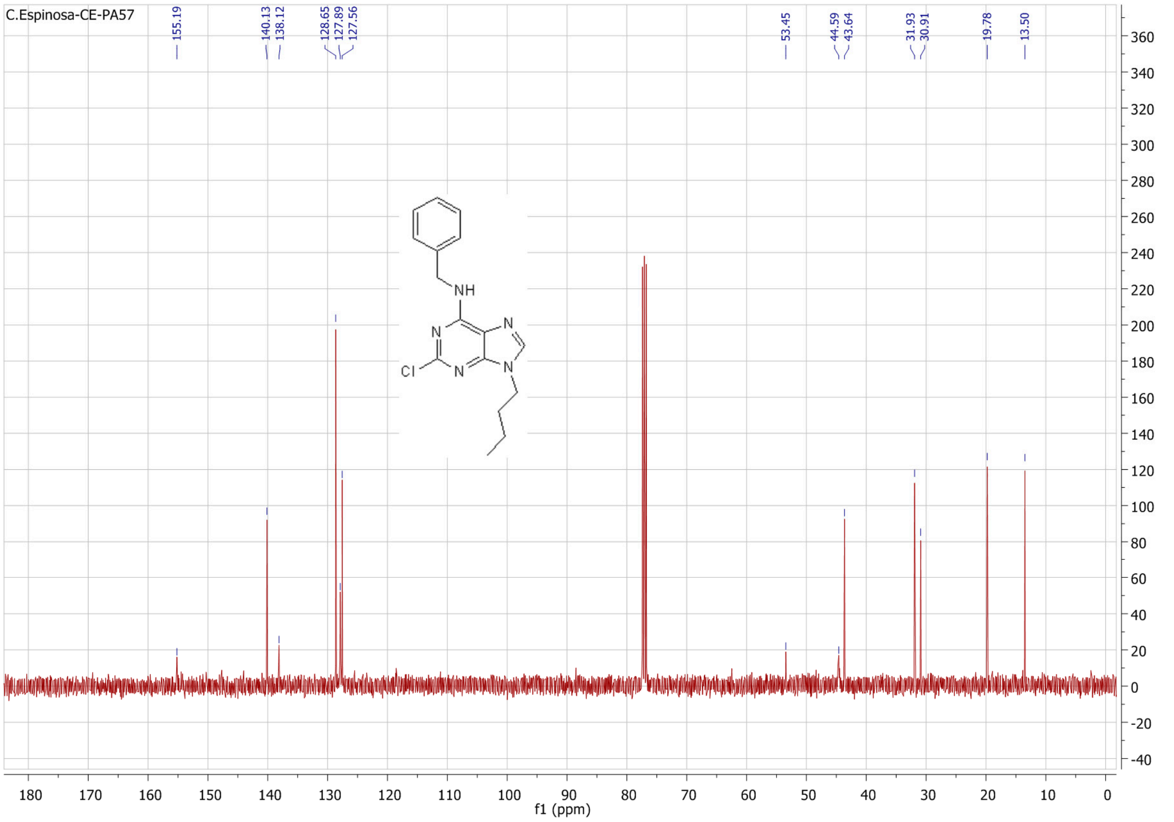

Figure S28. ¹³C-NMR for compound **3b**.

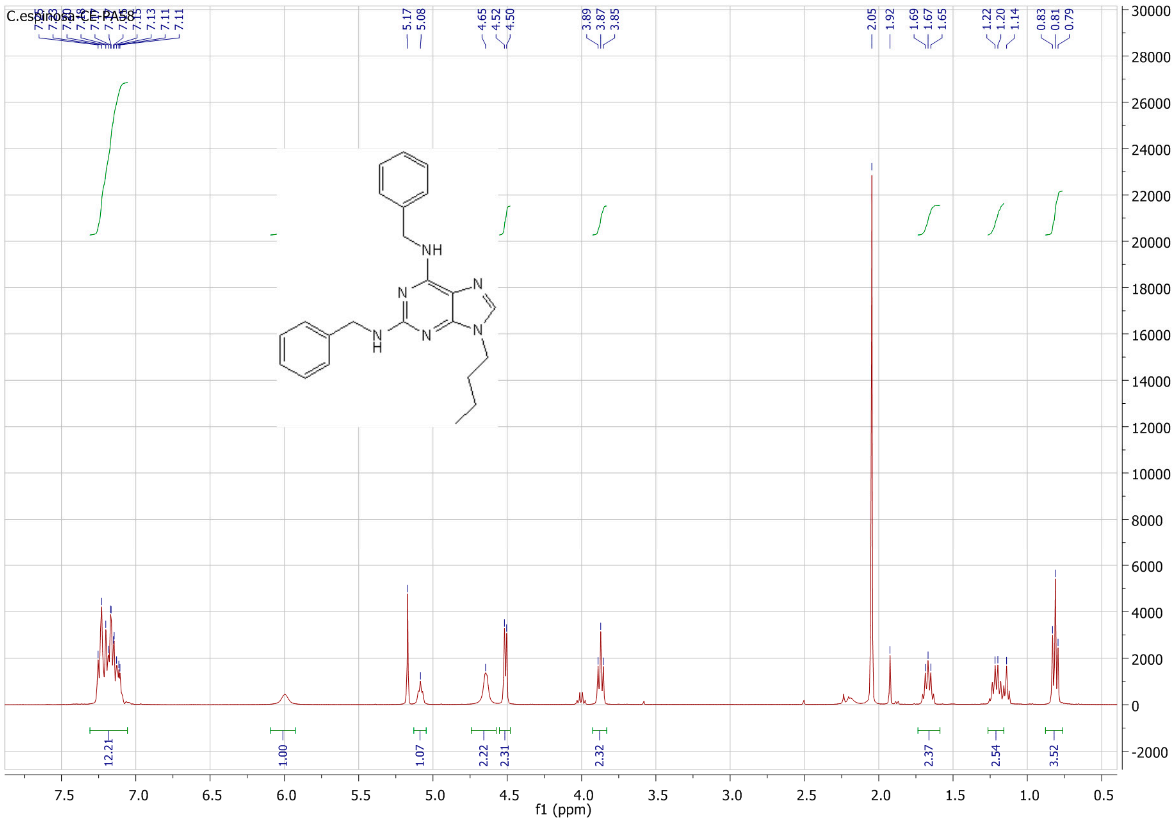

Figure S29. ^{1}H-NMR for compound **4b**.

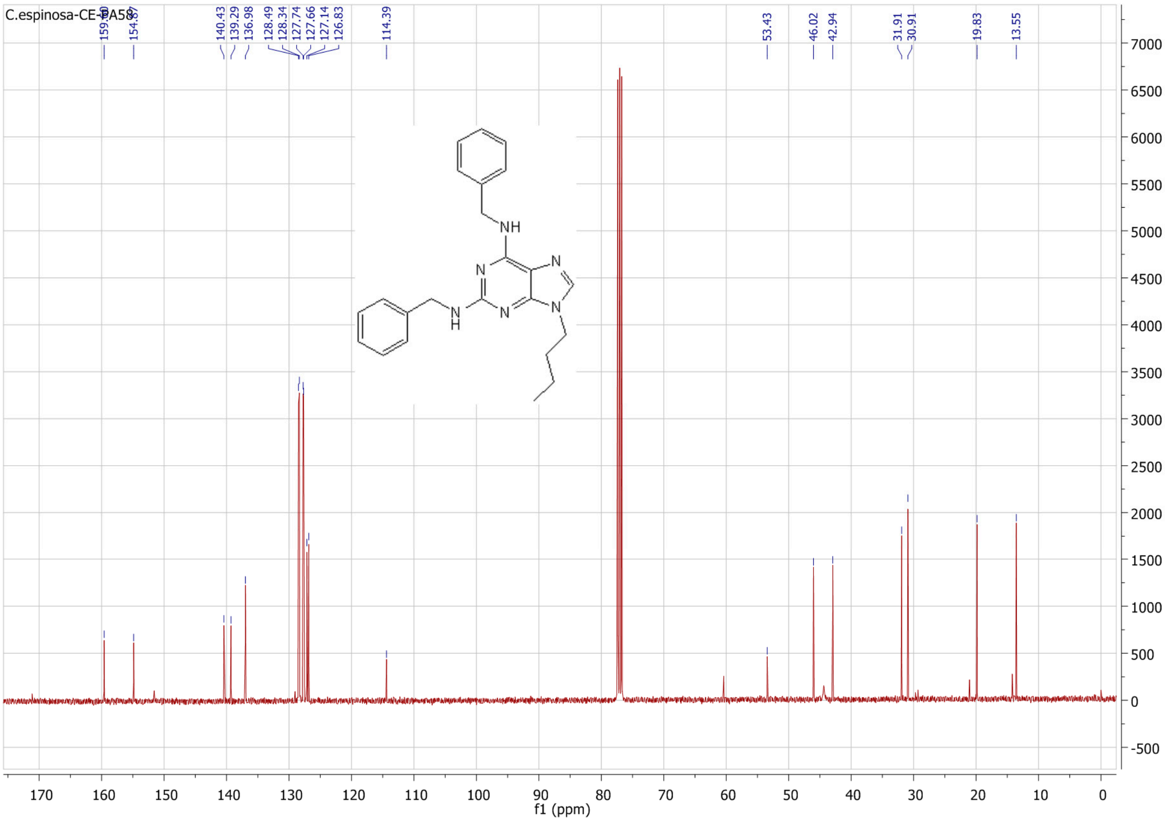

Figure S30. ^{13}C-NMR for compound **4b**.

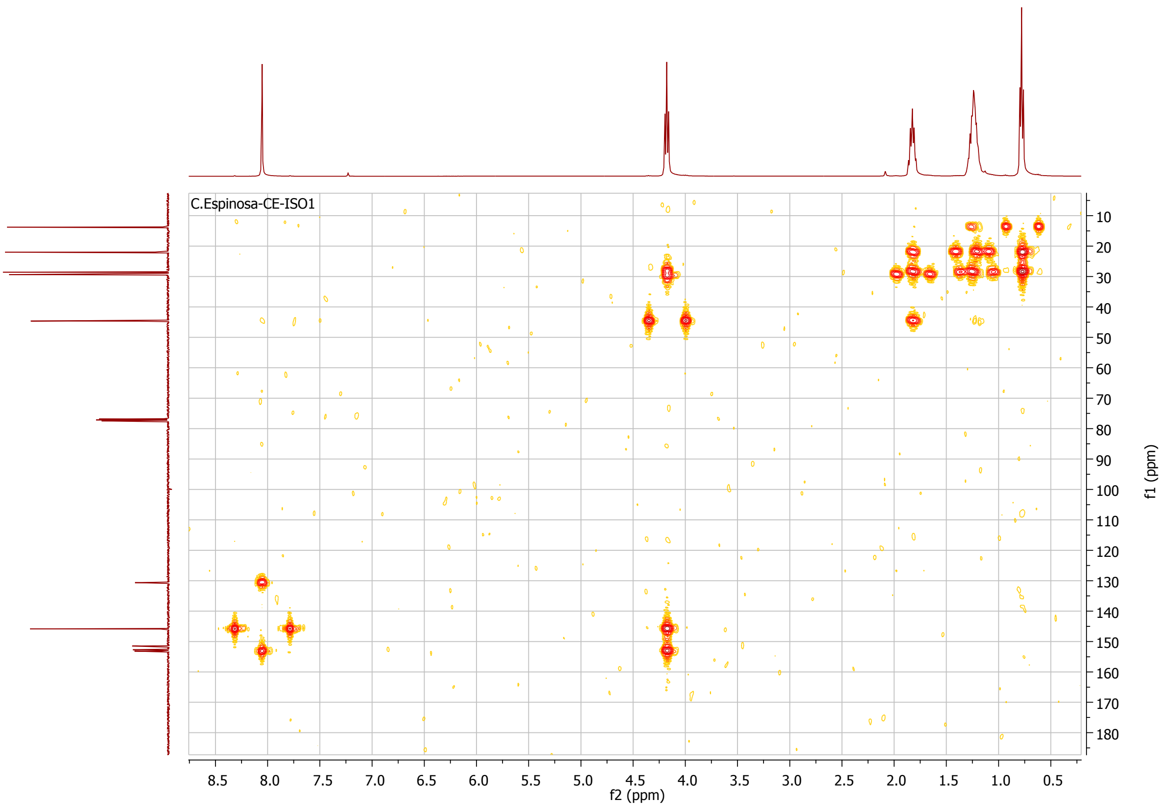

Figure S31. HMBC for compound **2b**.

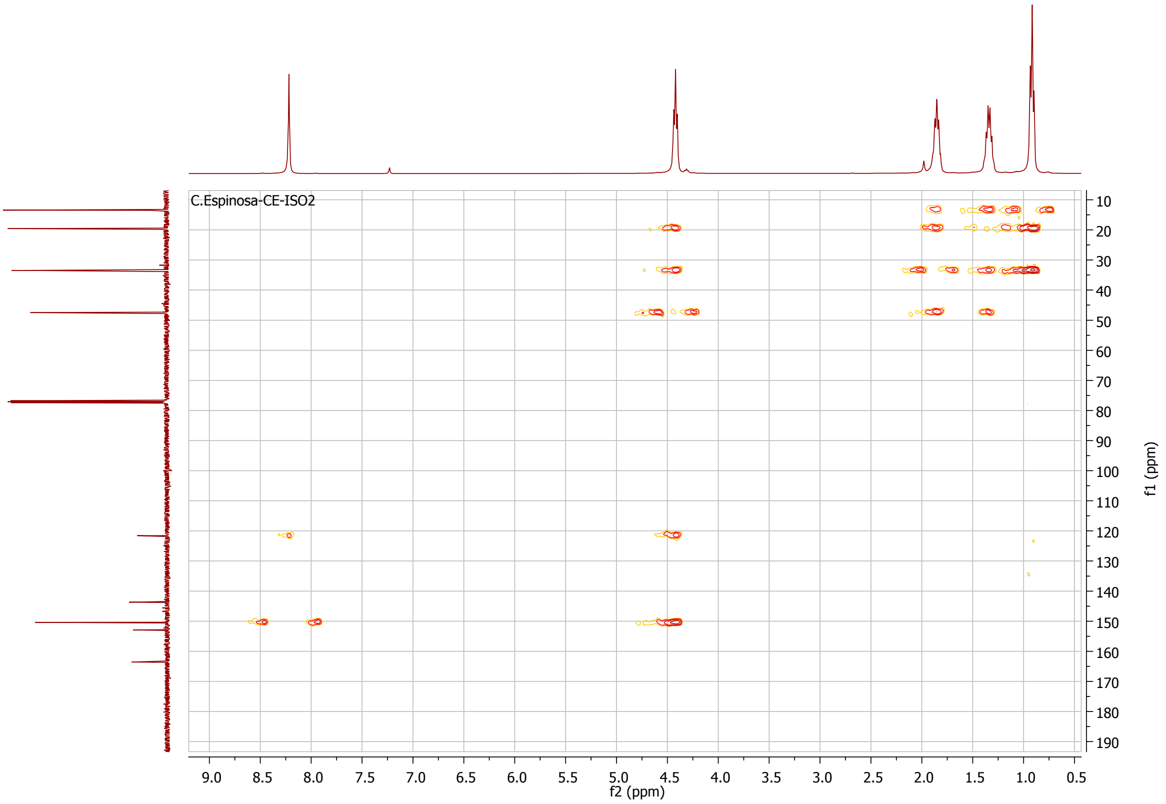

Figure S32. HMBC for compound **2b'**.

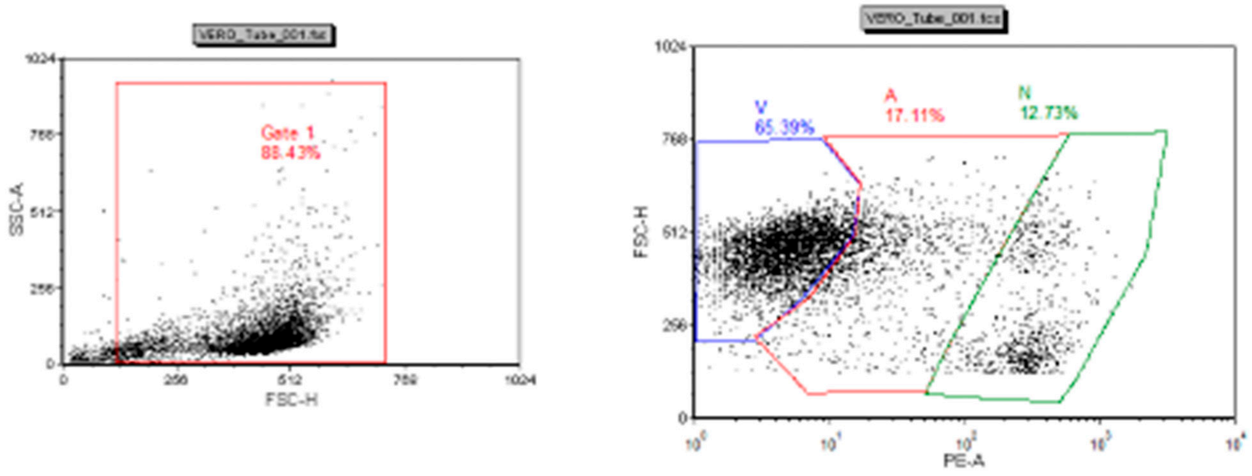

Overlay #	Filename	Gate	# of Events	X Geometric Mean	Y Geometric Mean	% of gated cells	% of all cells
1	VERO_Tube_001.fcs	None	5593	9.75	406.33	100.00	88.43
1	VERO_Tube_001.fcs	Gate 1	5593	9.75	406.33	100.00	88.43
1	VERO_Tube_001.fcs	Gate 2	3657	4.51	456.17	65.39	57.82
1	VERO_Tube_001.fcs	Gate 3	957	25.40	403.18	17.11	15.13
1	VERO_Tube_001.fcs	Gate 4	712	297.58	237.09	12.73	11.26

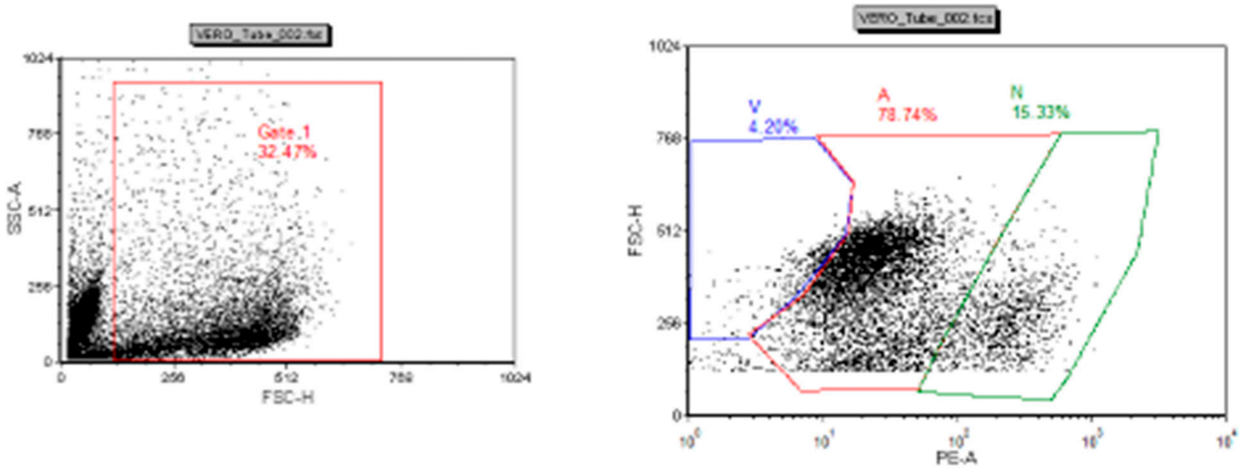

Overlay #	Filename	Gate	# of Events	X Geometric Mean	Y Geometric Mean	% of gated cells	% of all cells
1	VERO_Tube_002.fcs	None	6763	30.34	322.97	100.00	32.47
1	VERO_Tube_002.fcs	Gate 1	6763	30.34	322.97	100.00	32.47
1	VERO_Tube_002.fcs	Gate 2	284	6.70	396.19	4.20	1.36
1	VERO_Tube_002.fcs	Gate 3	5325	23.87	340.60	78.74	25.56
1	VERO_Tube_002.fcs	Gate 4	1037	223.07	245.86	15.33	4.98

Figure S33. *Cont.*

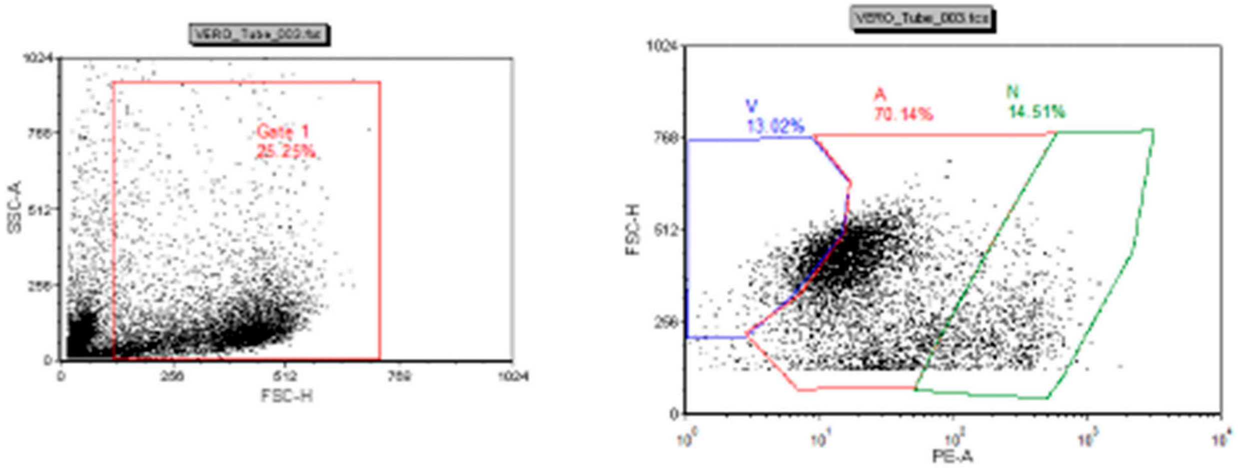

Overlay #	Filename	Gate	# of Events	X Geometric Mean	Y Geometric Mean	% of gated cells	% of all cells
1	VERO_Tube_003.fcs	None	6051	25.13	331.30	100.00	25.25
1	VERO_Tube_003.fcs	Gate 1	6051	25.13	331.30	100.00	25.25
1	VERO_Tube_003.fcs	Gate 2	788	7.98	426.40	13.02	3.29
1	VERO_Tube_003.fcs	Gate 3	4244	21.13	346.57	70.14	17.71
1	VERO_Tube_003.fcs	Gate 4	878	219.99	220.07	14.51	3.66

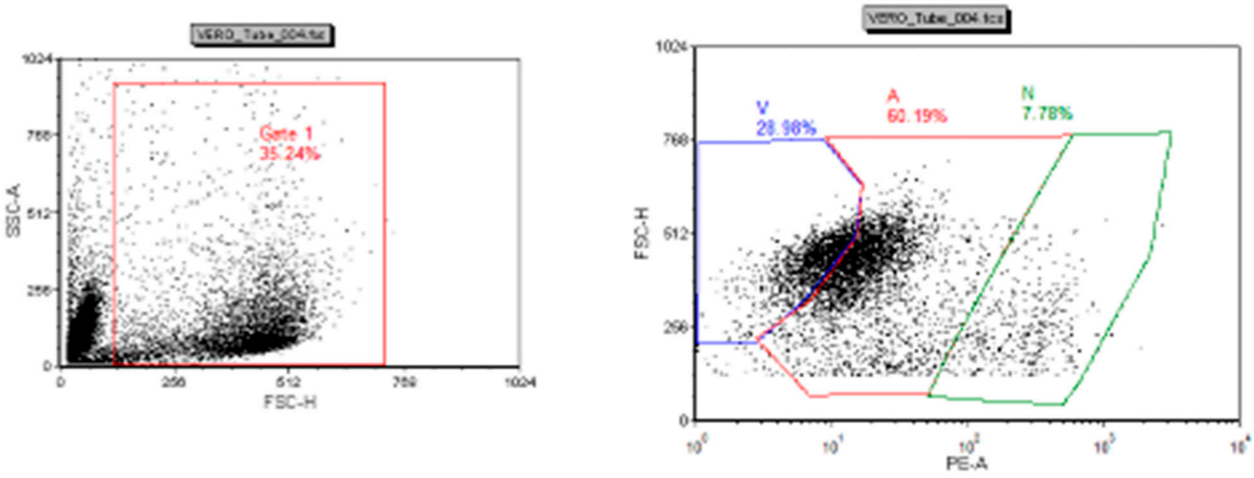

Overlay #	Filename	Gate	# of Events	X Geometric Mean	Y Geometric Mean	% of gated cells	% of all cells
1	VERO_Tube_004.fcs	None	5577	17.10	373.00	100.00	35.24
1	VERO_Tube_004.fcs	Gate 1	5577	17.10	373.00	100.00	35.24
1	VERO_Tube_004.fcs	Gate 2	1616	7.51	433.27	28.98	10.21
1	VERO_Tube_004.fcs	Gate 3	3357	19.36	374.22	60.19	21.21
1	VERO_Tube_004.fcs	Gate 4	434	238.31	230.65	7.78	2.74

Figure S33. *Cont.*

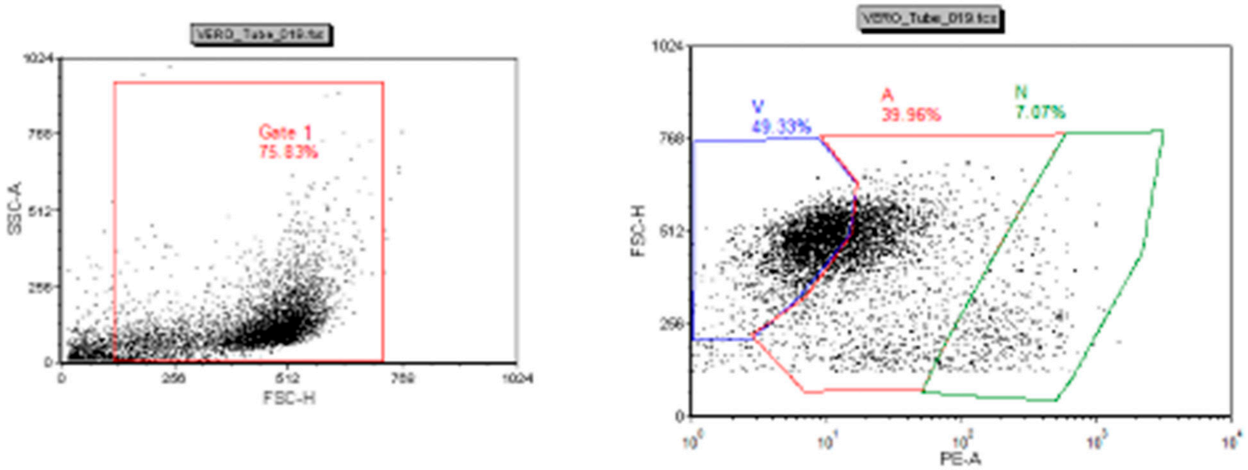

Overlay #	Filename	Gate	# of Events	X Geometric Mean	Y Geometric Mean	% of gated cells	% of all cells
1	VERO_Tube_019.fcs	None	5463	14.34	413.95	100.00	75.83
1	VERO_Tube_019.fcs	Gate 1	5463	14.34	413.95	100.00	75.83
1	VERO_Tube_019.fcs	Gate 2	2695	7.48	473.74	49.33	37.41
1	VERO_Tube_019.fcs	Gate 3	2183	23.43	407.23	39.96	30.30
1	VERO_Tube_019.fcs	Gate 4	386	218.22	233.72	7.07	5.36

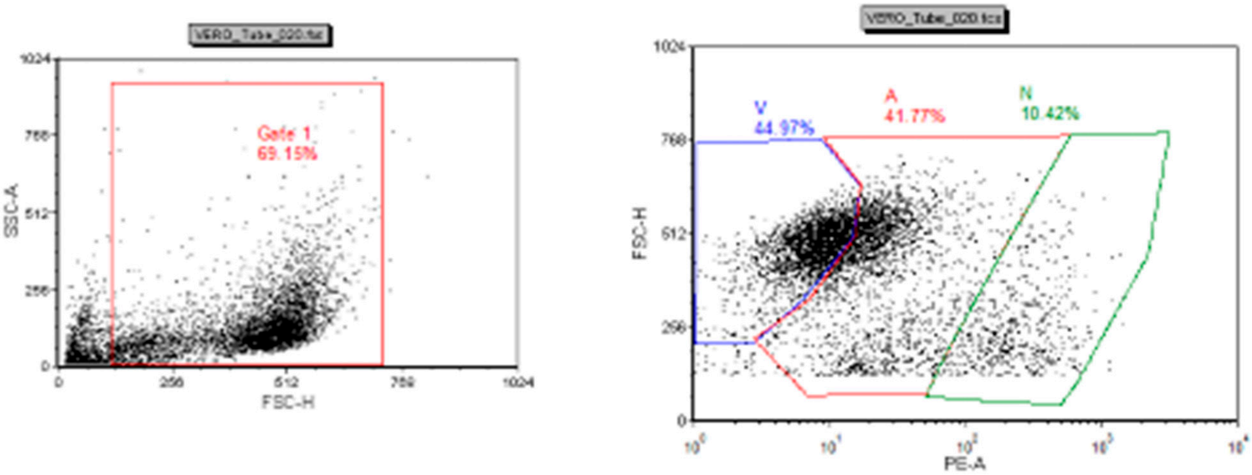

Overlay #	Filename	Gate	# of Events	X Geometric Mean	Y Geometric Mean	% of gated cells	% of all cells
1	VERO_Tube_020.fcs	None	5604	16.96	399.27	100.00	69.15
1	VERO_Tube_020.fcs	Gate 1	5604	16.96	399.27	100.00	69.15
1	VERO_Tube_020.fcs	Gate 2	2520	7.52	472.32	44.97	31.10
1	VERO_Tube_020.fcs	Gate 3	2341	23.61	392.87	41.77	28.89
1	VERO_Tube_020.fcs	Gate 4	584	236.93	232.93	10.42	7.21

Figure S33. *Cont.*

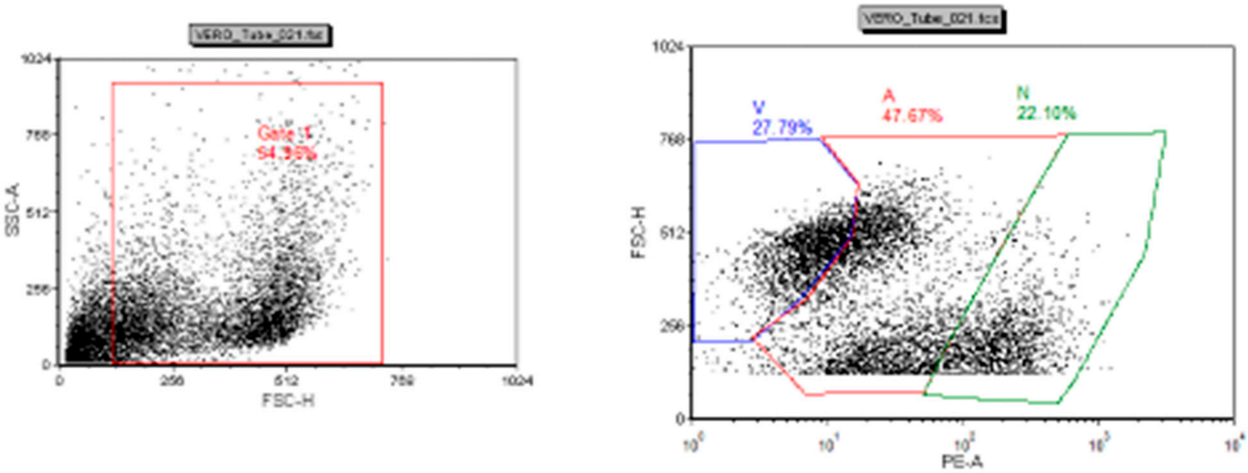

Overlay #	Filename	Gate	# of Events	X Geometric Mean	Y Geometric Mean	% of gated cells	% of all cells
1	VERO_Tube_021.fcs	None	6910	25.92	309.18	100.00	54.96
1	VERO_Tube_021.fcs	Gate 1	6910	25.92	309.18	100.00	54.96
1	VERO_Tube_021.fcs	Gate 2	1920	7.08	452.32	27.79	15.27
1	VERO_Tube_021.fcs	Gate 3	3294	24.72	301.12	47.67	26.20
1	VERO_Tube_021.fcs	Gate 4	1527	196.78	207.55	22.10	12.15

Figure S33. Flow cytometry analysis on VERO cells.

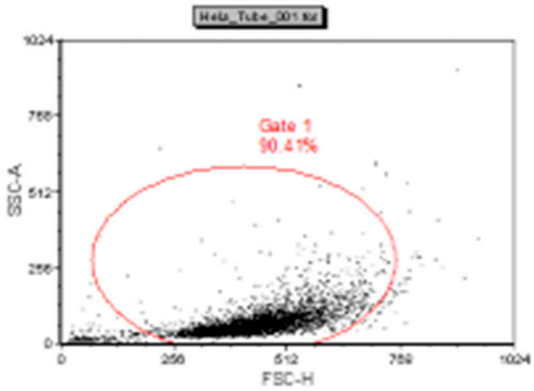

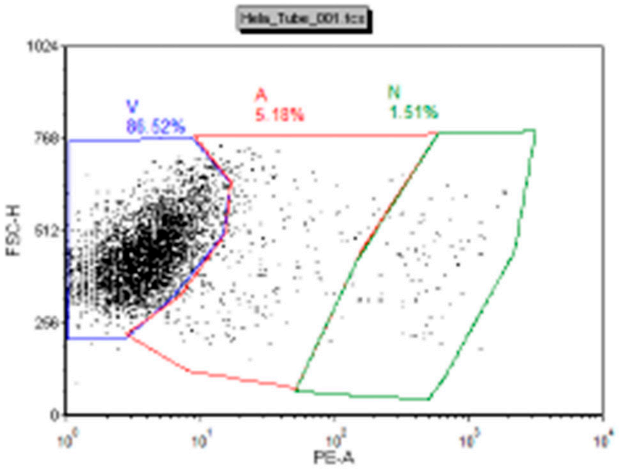

Overlay #	Filename	Gate	# of Events	X Geometric Mean	Y Geometric Mean	% of gated cells	% of all cells
1	Hela_Tube_001.fcs	None	4977	4.21	432.52	100.00	90.41
1	Hela_Tube_001.fcs	Gate 1	4977	4.21	432.52	100.00	90.41
1	Hela_Tube_001.fcs	Gate 2	4306	3.90	438.01	86.52	78.22
1	Hela_Tube_001.fcs	Gate 3	258	22.32	429.95	5.18	4.69
1	Hela_Tube_001.fcs	Gate 4	75	469.42	392.42	1.51	1.36

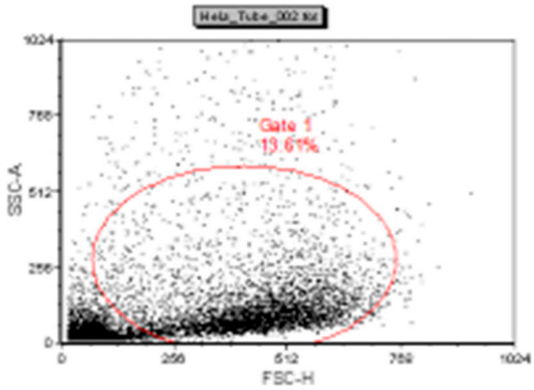

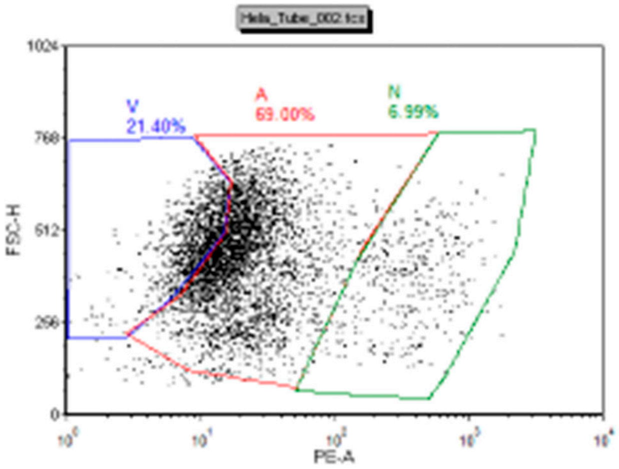

Overlay #	Filename	Gate	# of Events	X Geometric Mean	Y Geometric Mean	% of gated cells	% of all cells
1	Hela_Tube_002.fcs	None	5262	21.44	410.44	100.00	13.61
1	Hela_Tube_002.fcs	Gate 1	5262	21.44	410.44	100.00	13.61
1	Hela_Tube_002.fcs	Gate 2	1126	9.38	485.80	21.40	2.91
1	Hela_Tube_002.fcs	Gate 3	3631	21.77	405.13	69.00	9.39
1	Hela_Tube_002.fcs	Gate 4	368	355.52	343.76	6.99	0.95

Figure S34. *Cont.*

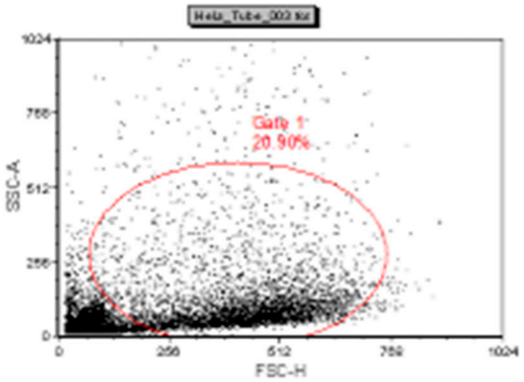

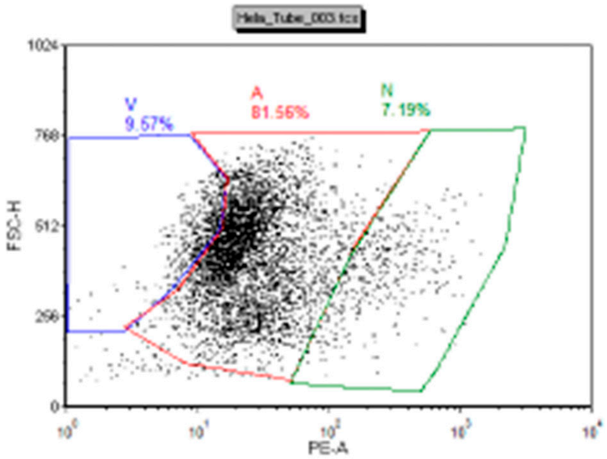

Overlay #	Filename	Gate	# of Events	X Geometric Mean	Y Geometric Mean	% of gated cells	% of all cells
1	Hela_Tube_003.fcs	None	5358	27.13	401.61	100.00	20.90
1	Hela_Tube_003.fcs	Gate 1	5358	27.13	401.61	100.00	20.90
1	Hela_Tube_003.fcs	Gate 2	513	10.84	505.38	9.57	2.00
1	Hela_Tube_003.fcs	Gate 3	4370	25.78	403.39	81.56	17.04
1	Hela_Tube_003.fcs	Gate 4	385	247.83	345.55	7.19	1.50

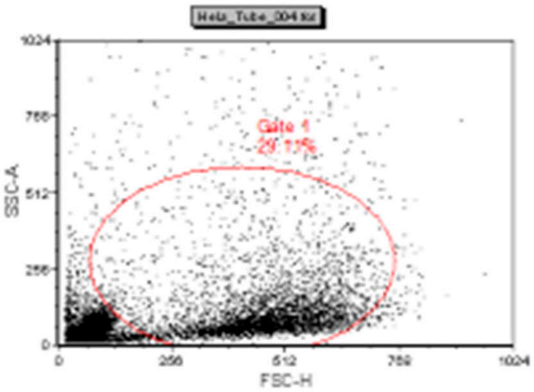

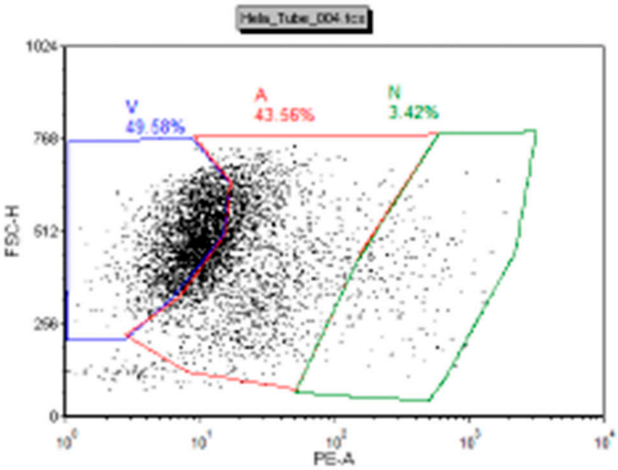

Overlay #	Filename	Gate	# of Events	X Geometric Mean	Y Geometric Mean	% of gated cells	% of all cells
1	Hela_Tube_004.fcs	None	5030	14.10	439.04	100.00	29.11
1	Hela_Tube_004.fcs	Gate 1	5030	14.10	439.04	100.00	29.11
1	Hela_Tube_004.fcs	Gate 2	2494	8.32	490.26	49.58	14.43
1	Hela_Tube_004.fcs	Gate 3	2191	21.79	414.31	43.56	12.68
1	Hela_Tube_004.fcs	Gate 4	172	313.64	364.76	3.42	1.00

Figure S34. *Cont.*

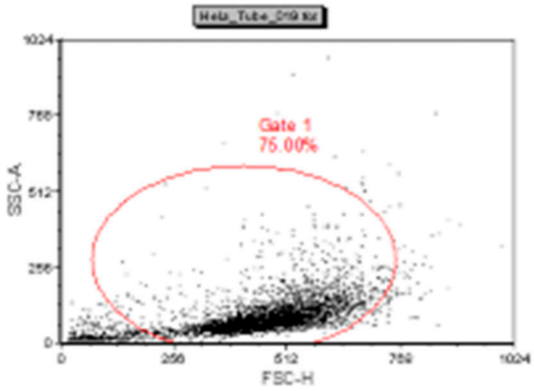

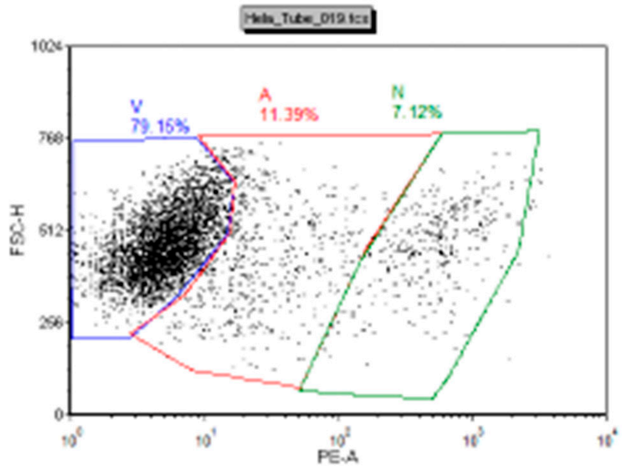

Overlay #	Filename	Gate	# of Events	X Geometric Mean	Y Geometric Mean	% of gated cells	% of all cells
1	Hela_Tube_019.fcs	None	4970	8.27	457.59	100.00	75.00
1	Hela_Tube_019.fcs	Gate 1	4970	8.27	457.59	100.00	75.00
1	Hela_Tube_019.fcs	Gate 2	3934	5.03	465.50	79.15	59.36
1	Hela_Tube_019.fcs	Gate 3	566	25.83	424.04	11.39	8.54
1	Hela_Tube_019.fcs	Gate 4	354	515.29	452.36	7.12	5.34

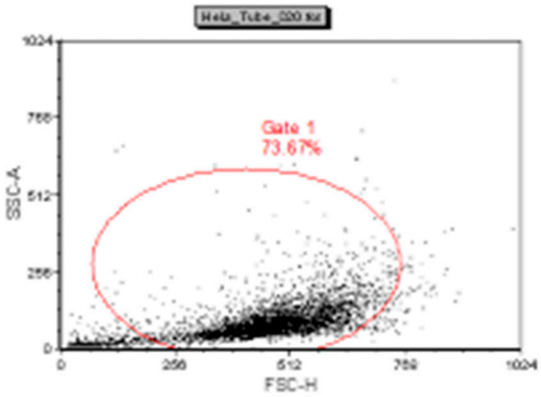

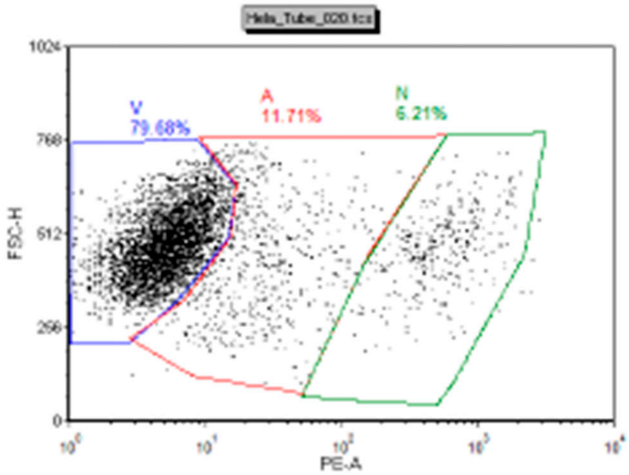

Overlay #	Filename	Gate	# of Events	X Geometric Mean	Y Geometric Mean	% of gated cells	% of all cells
1	Hela_Tube_020.fcs	None	4946	8.02	464.25	100.00	73.67
1	Hela_Tube_020.fcs	Gate 1	4946	8.02	464.25	100.00	73.67
1	Hela_Tube_020.fcs	Gate 2	3941	5.06	473.92	79.68	58.70
1	Hela_Tube_020.fcs	Gate 3	579	24.94	422.91	11.71	8.62
1	Hela_Tube_020.fcs	Gate 4	307	545.82	458.15	6.21	4.57

Figure S34. *Cont.*

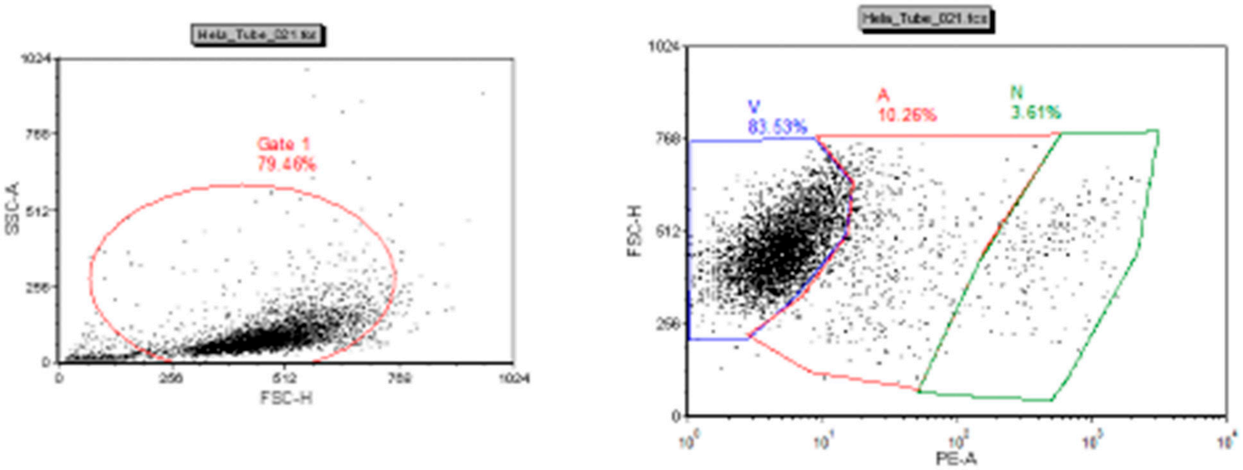

Overlay #	Filename	Gate	# of Events	X Geometric Mean	Y Geometric Mean	% of gated cells	% of all cells
1	Hela_Tube_021.fcs	None	4962	6.69	454.80	100.00	79.46
1	Hela_Tube_021.fcs	Gate 1	4962	6.69	454.80	100.00	79.46
1	Hela_Tube_021.fcs	Gate 2	4145	4.93	459.37	83.53	66.37
1	Hela_Tube_021.fcs	Gate 3	509	25.69	446.34	10.26	8.15
1	Hela_Tube_021.fcs	Gate 4	179	505.47	438.11	3.61	2.87

Figure S34. Flow cytometry analysis on HeLa cells.

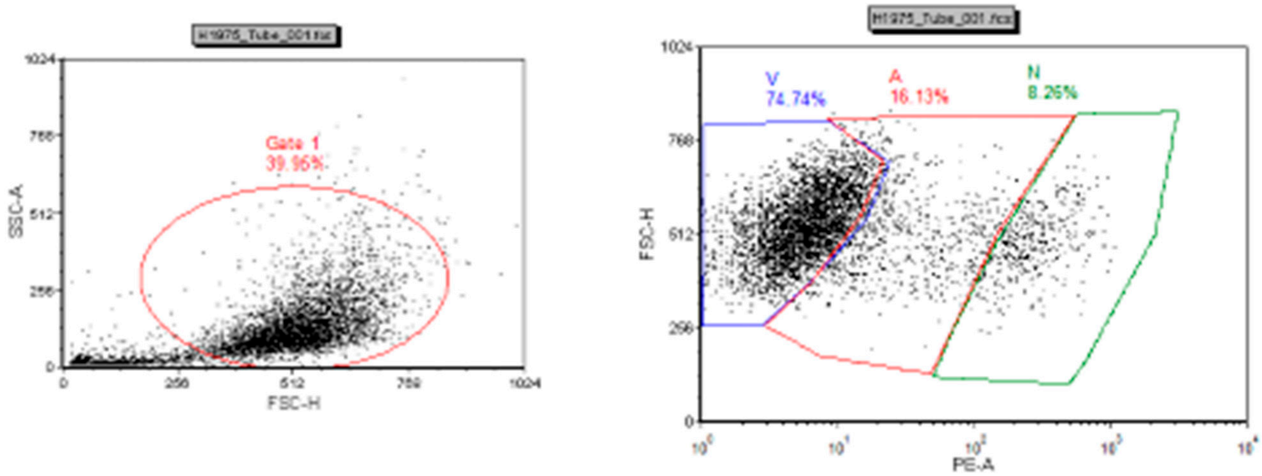

Overlay #	Filename	Gate	# of Events	X Geometric Mean	Y Geometric Mean	% of gated cells	% of all cells
1	H1975_Tube_001.fcs	None	4917	9.55	518.15	100.00	39.95
1	H1975_Tube_001.fcs	Gate 1	4917	9.55	518.15	100.00	39.95
1	H1975_Tube_001.fcs	Gate 2	3675	5.68	530.92	74.74	29.86
1	H1975_Tube_001.fcs	Gate 3	793	28.23	500.79	16.13	6.44
1	H1975_Tube_001.fcs	Gate 4	406	284.81	478.10	8.26	3.30

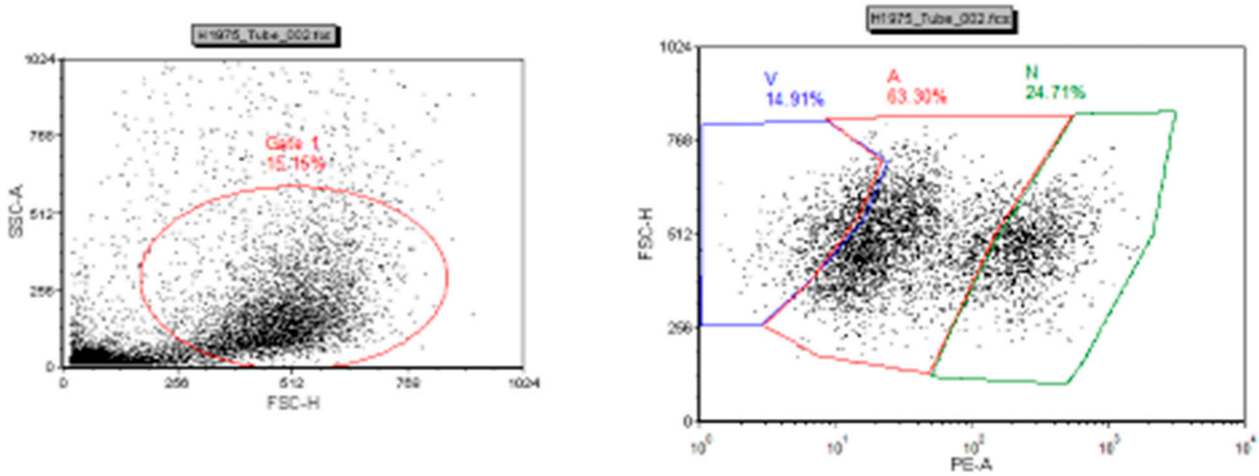

Overlay #	Filename	Gate	# of Events	X Geometric Mean	Y Geometric Mean	% of gated cells	% of all cells
1	H1975_Tube_002.fcs	None	4480	42.19	477.56	100.00	15.15
1	H1975_Tube_002.fcs	Gate 1	4480	42.19	477.56	100.00	15.15
1	H1975_Tube_002.fcs	Gate 2	668	9.37	535.61	14.91	2.26
1	H1975_Tube_002.fcs	Gate 3	2836	27.28	475.43	63.30	9.59
1	H1975_Tube_002.fcs	Gate 4	1107	279.22	461.41	24.71	3.74

Figure S35. *Cont.*

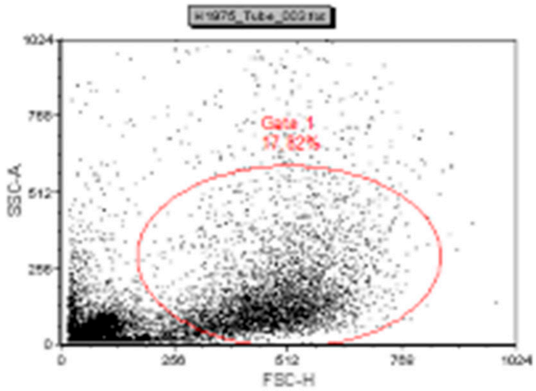

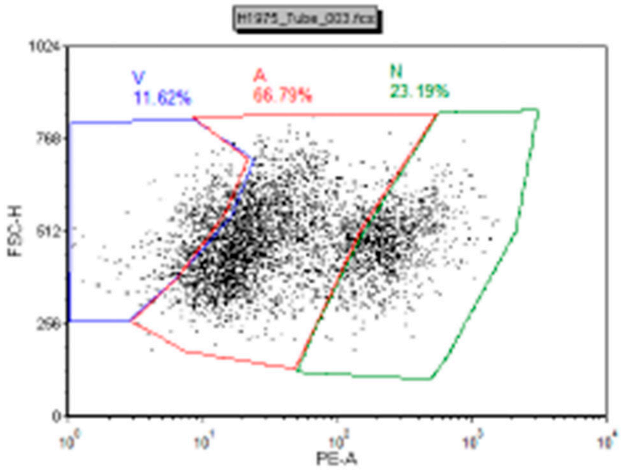

Overlay #	Filename	Gate	# of Events	X Geometric Mean	Y Geometric Mean	% of gated cells	% of all cells
1	H1975_Tube_003.fcs	None	4408	41.78	471.53	100.00	17.82
1	H1975_Tube_003.fcs	Gate 1	4408	41.78	471.53	100.00	17.82
1	H1975_Tube_003.fcs	Gate 2	512	9.02	520.48	11.62	2.07
1	H1975_Tube_003.fcs	Gate 3	2944	27.66	469.56	66.79	11.90
1	H1975_Tube_003.fcs	Gate 4	1022	266.16	461.89	23.19	4.13

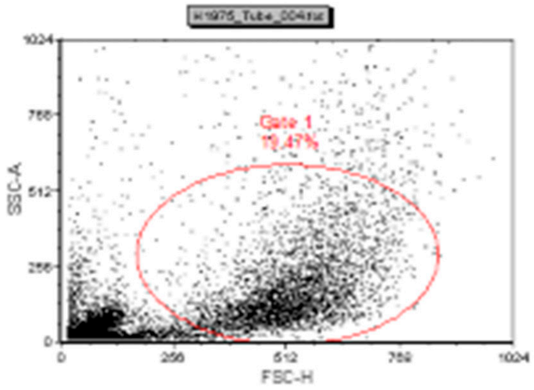

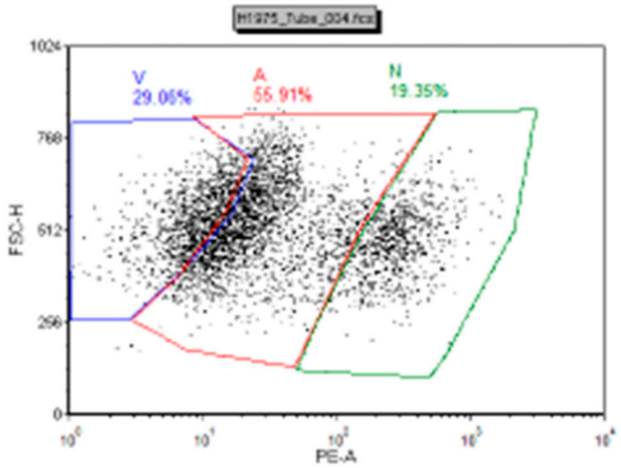

Overlay #	Filename	Gate	# of Events	X Geometric Mean	Y Geometric Mean	% of gated cells	% of all cells
1	H1975_Tube_004.fcs	None	4418	31.46	509.22	100.00	19.47
1	H1975_Tube_004.fcs	Gate 1	4418	31.46	509.22	100.00	19.47
1	H1975_Tube_004.fcs	Gate 2	1284	9.16	544.88	29.06	5.66
1	H1975_Tube_004.fcs	Gate 3	2470	26.06	511.22	55.91	10.88
1	H1975_Tube_004.fcs	Gate 4	855	290.52	466.55	19.35	3.77

Figure S35. *Cont.*

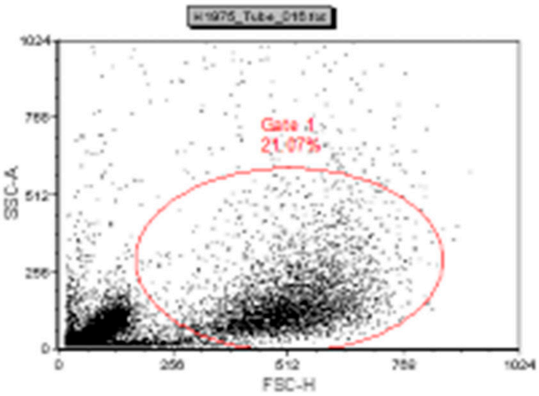

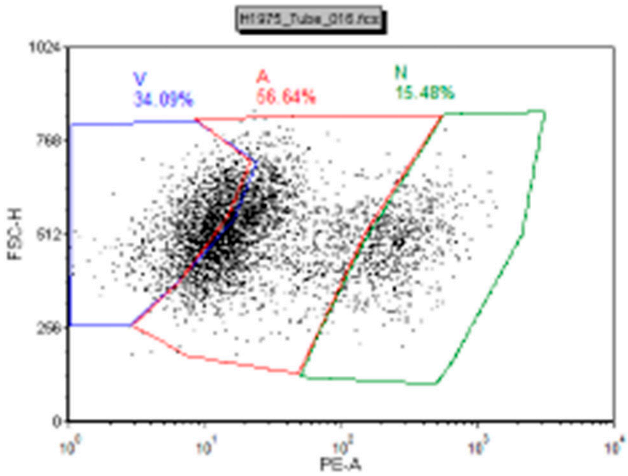

Overlay #	Filename	Gate	# of Events	X Geometric Mean	Y Geometric Mean	% of gated cells	% of all cells
1	H1975_Tube_016.fcs	None	4555	25.32	501.53	100.00	21.07
1	H1975_Tube_016.fcs	Gate 1	4555	25.32	501.53	100.00	21.07
1	H1975_Tube_016.fcs	Gate 2	1553	9.55	542.78	34.09	7.18
1	H1975_Tube_016.fcs	Gate 3	2580	22.48	494.22	56.64	11.93
1	H1975_Tube_016.fcs	Gate 4	705	272.57	465.69	15.48	3.26

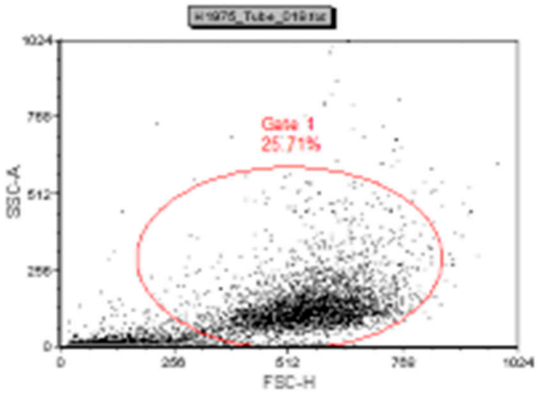

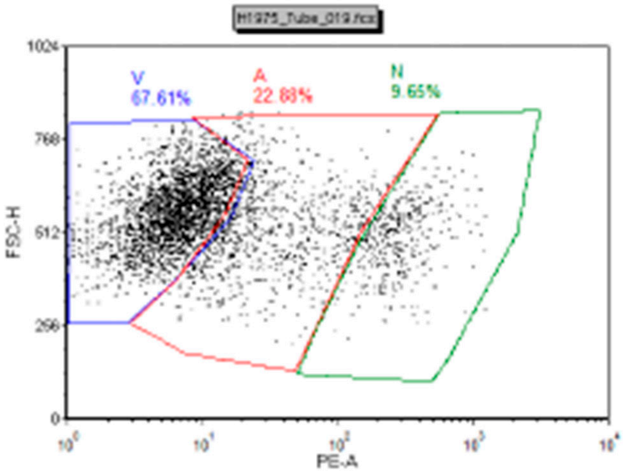

Overlay #	Filename	Gate	# of Events	X Geometric Mean	Y Geometric Mean	% of gated cells	% of all cells
1	H1975_Tube_019.fcs	None	4112	12.51	542.70	100.00	25.71
1	H1975_Tube_019.fcs	Gate 1	4112	12.51	542.70	100.00	25.71
1	H1975_Tube_019.fcs	Gate 2	2780	6.57	566.21	67.61	17.38
1	H1975_Tube_019.fcs	Gate 3	941	29.96	512.93	22.88	5.88
1	H1975_Tube_019.fcs	Gate 4	397	295.13	486.44	9.65	2.48

Figure S35. *Cont.*

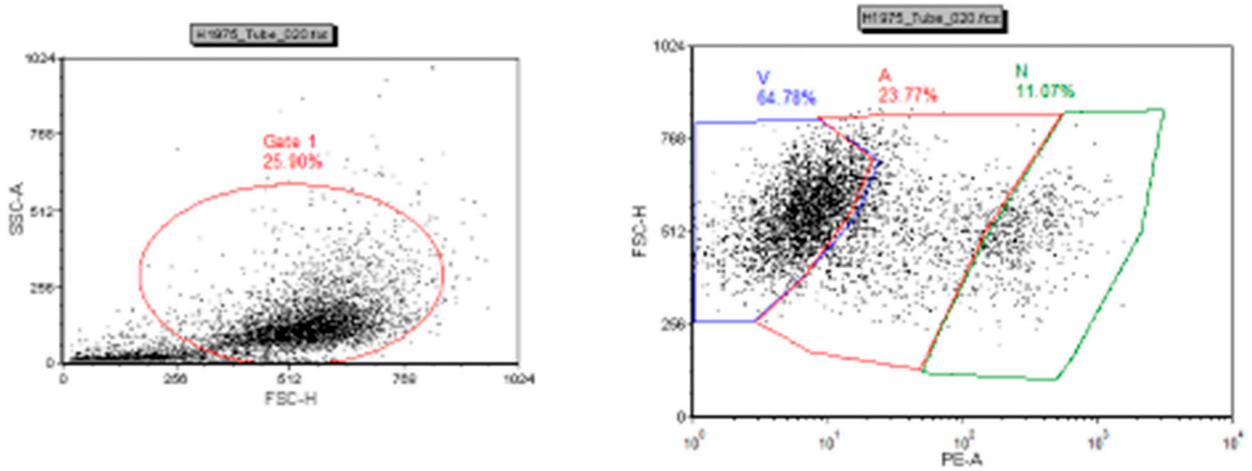

Overlay #	Filename	Gate	# of Events	X Geometric Mean	Y Geometric Mean	% of gated cells	% of all cells
1	H1975_Tube_020.fcs	None	4245	13.72	537.31	100.00	25.90
1	H1975_Tube_020.fcs	Gate 1	4245	13.72	537.31	100.00	25.90
1	H1975_Tube_020.fcs	Gate 2	2750	6.71	560.33	64.78	16.78
1	H1975_Tube_020.fcs	Gate 3	1009	30.09	514.49	23.77	6.16
1	H1975_Tube_020.fcs	Gate 4	470	300.37	483.19	11.07	2.87

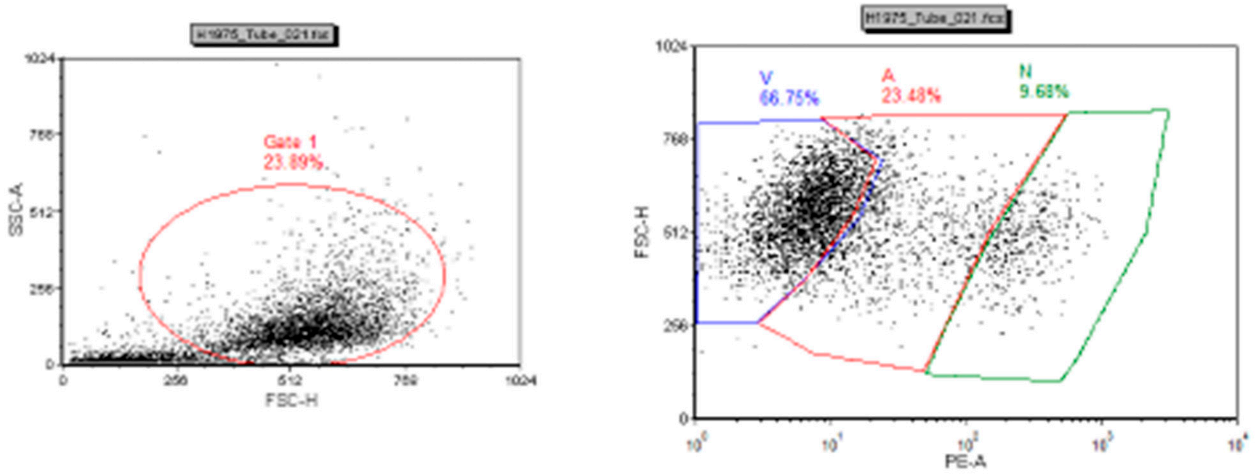

Overlay #	Filename	Gate	# of Events	X Geometric Mean	Y Geometric Mean	% of gated cells	% of all cells
1	H1975_Tube_021.fcs	None	4204	12.67	540.89	100.00	23.89
1	H1975_Tube_021.fcs	Gate 1	4204	12.67	540.89	100.00	23.89
1	H1975_Tube_021.fcs	Gate 2	2806	6.75	564.72	66.75	15.95
1	H1975_Tube_021.fcs	Gate 3	987	28.88	519.49	23.48	5.61
1	H1975_Tube_021.fcs	Gate 4	407	273.36	488.14	9.68	2.31

Figure S35. Flow cytometry analysis on HT1975 cells.

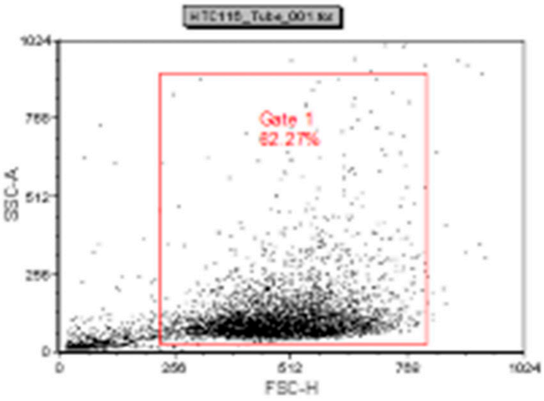

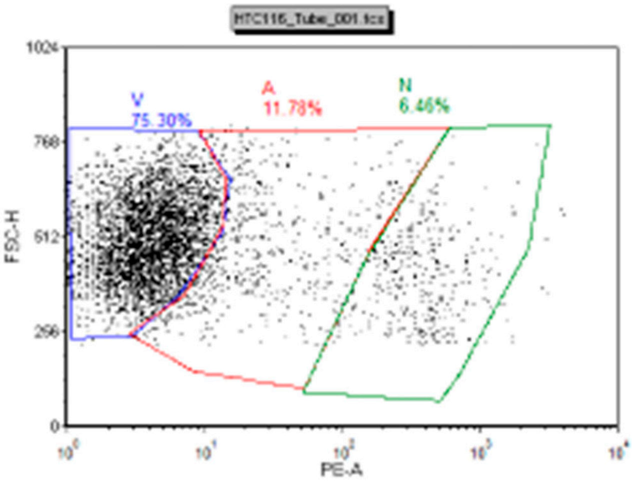

Overlay #	Filename	Gate	# of Events	X Geometric Mean	Y Geometric Mean	% of gated cells	% of all cells
1	HTC116_Tube_001.fcs	None	5061	6.05	479.63	100.00	82.27
1	HTC116_Tube_001.fcs	Gate 1	5061	6.05	479.63	100.00	82.27
1	HTC116_Tube_001.fcs	Gate 2	3811	3.97	495.03	75.30	61.95
1	HTC116_Tube_001.fcs	Gate 3	596	23.07	458.05	11.78	9.69
1	HTC116_Tube_001.fcs	Gate 4	327	366.83	386.65	6.46	5.32

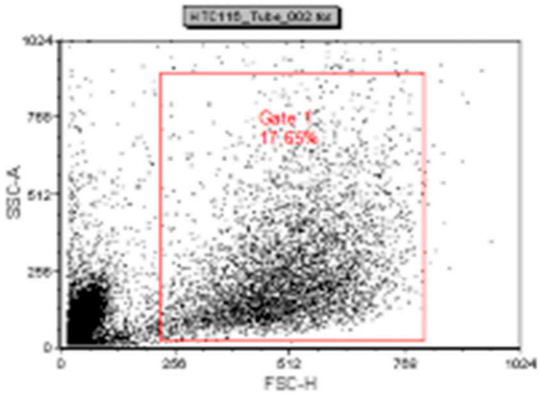

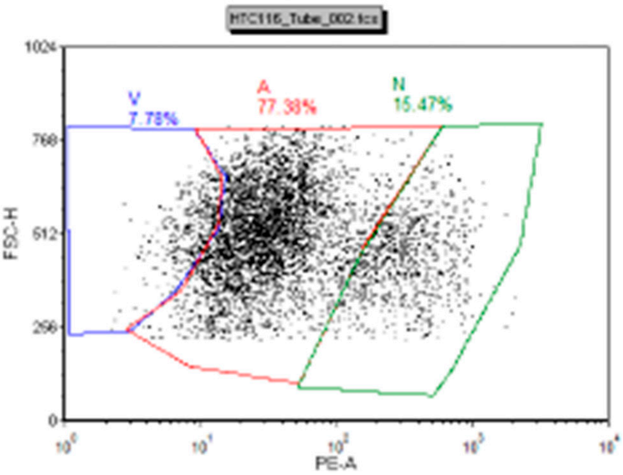

Overlay #	Filename	Gate	# of Events	X Geometric Mean	Y Geometric Mean	% of gated cells	% of all cells
1	HTC116_Tube_002.fcs	None	5631	41.86	487.65	100.00	17.65
1	HTC116_Tube_002.fcs	Gate 1	5631	41.86	487.65	100.00	17.65
1	HTC116_Tube_002.fcs	Gate 2	438	9.26	531.46	7.78	1.37
1	HTC116_Tube_002.fcs	Gate 3	4357	31.93	497.50	77.38	13.66
1	HTC116_Tube_002.fcs	Gate 4	871	328.14	426.11	15.47	2.73

Figure S36. *Cont.*

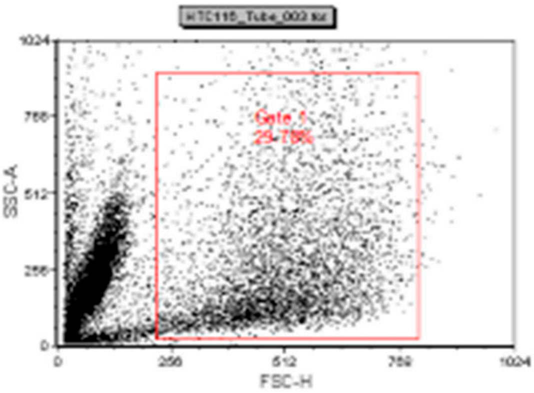

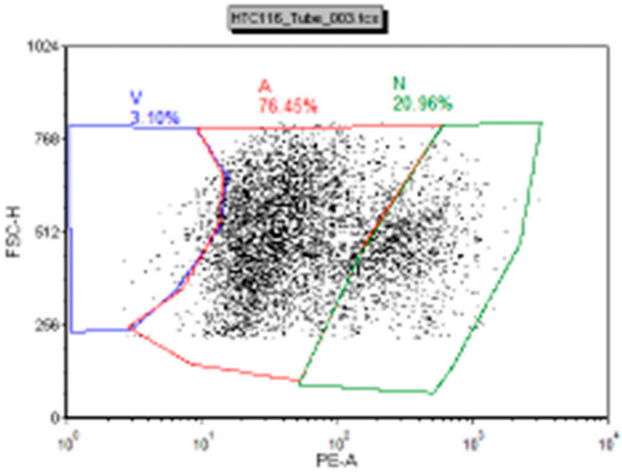

Overlay #	Filename	Gate	# of Events	X Geometric Mean	Y Geometric Mean	% of gated cells	% of all cells
1	HTC116_Tube_003.fcs	None	5133	58.58	479.89	100.00	29.78
1	HTC116_Tube_003.fcs	Gate 1	5133	58.58	479.89	100.00	29.78
1	HTC116_Tube_003.fcs	Gate 2	159	10.41	538.04	3.10	0.92
1	HTC116_Tube_003.fcs	Gate 3	3924	38.69	488.92	76.45	22.77
1	HTC116_Tube_003.fcs	Gate 4	1076	327.34	443.79	20.96	6.24

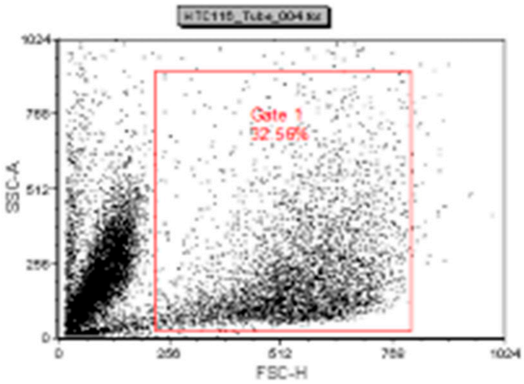

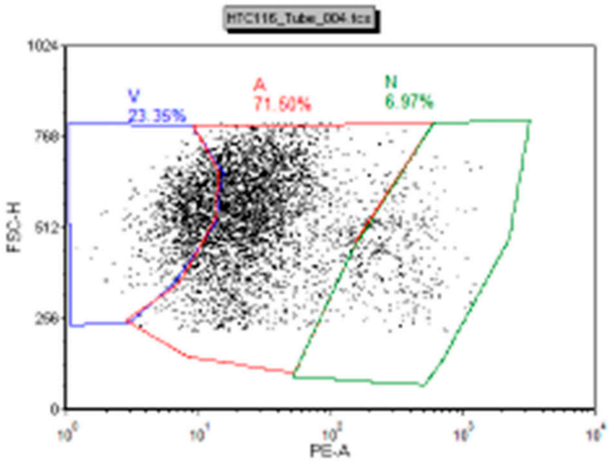

Overlay #	Filename	Gate	# of Events	X Geometric Mean	Y Geometric Mean	% of gated cells	% of all cells
1	HTC116_Tube_004.fcs	None	5049	25.42	523.78	100.00	32.56
1	HTC116_Tube_004.fcs	Gate 1	5049	25.42	523.78	100.00	32.56
1	HTC116_Tube_004.fcs	Gate 2	1179	8.74	558.76	23.35	7.60
1	HTC116_Tube_004.fcs	Gate 3	3610	27.77	525.88	71.50	23.28
1	HTC116_Tube_004.fcs	Gate 4	352	318.37	420.68	6.97	2.27

Figure S36. *Cont.*

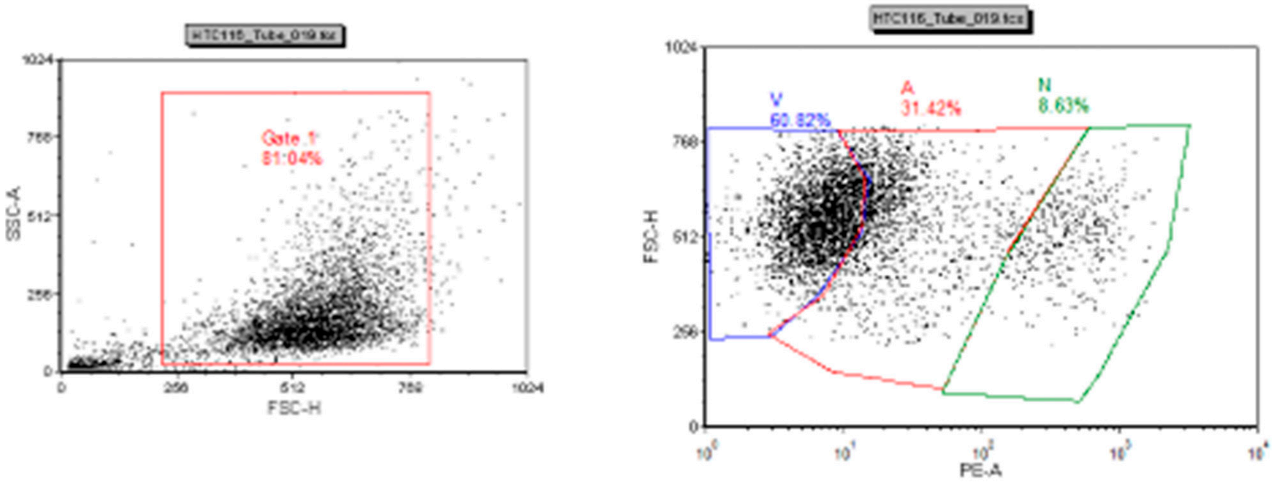

Overlay #	Filename	Gate	# of Events	X Geometric Mean	Y Geometric Mean	% of gated cells	% of all cells
1	HTC116_Tube_019.fcs	None	4959	14.30	547.89	100.00	81.04
1	HTC116_Tube_019.fcs	Gate 1	4959	14.30	547.89	100.00	81.04
1	HTC116_Tube_019.fcs	Gate 2	3016	6.83	557.49	60.82	49.29
1	HTC116_Tube_019.fcs	Gate 3	1558	24.79	558.37	31.42	25.46
1	HTC116_Tube_019.fcs	Gate 4	428	454.28	500.91	8.63	6.99

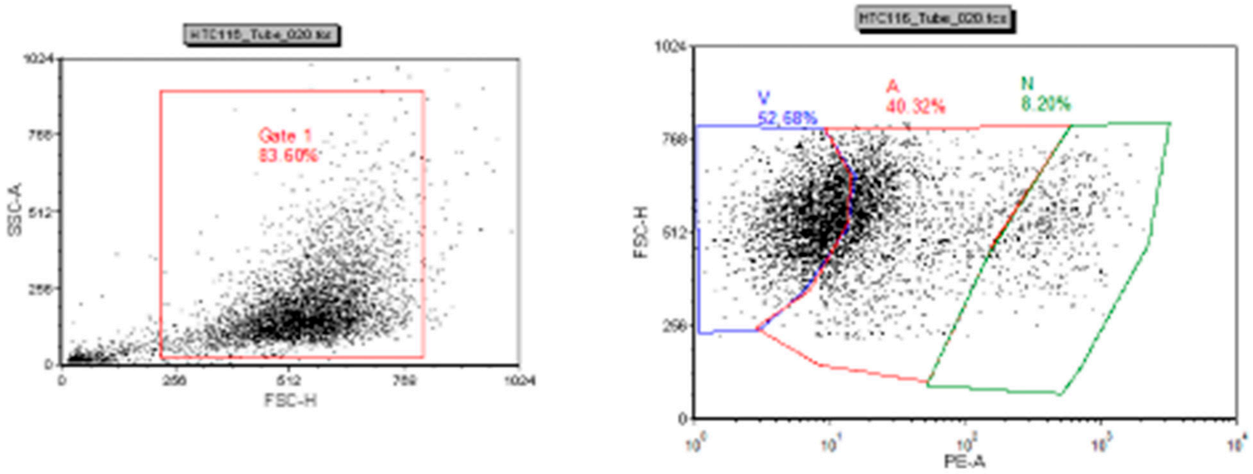

Overlay #	Filename	Gate	# of Events	X Geometric Mean	Y Geometric Mean	% of gated cells	% of all cells
1	HTC116_Tube_020.fcs	None	4985	16.27	537.19	100.00	83.60
1	HTC116_Tube_020.fcs	Gate 1	4985	16.27	537.19	100.00	83.60
1	HTC116_Tube_020.fcs	Gate 2	2626	7.20	546.28	52.68	44.04
1	HTC116_Tube_020.fcs	Gate 3	2010	25.17	535.08	40.32	33.71
1	HTC116_Tube_020.fcs	Gate 4	409	451.98	510.46	8.20	6.86

Figure S36. *Cont.*

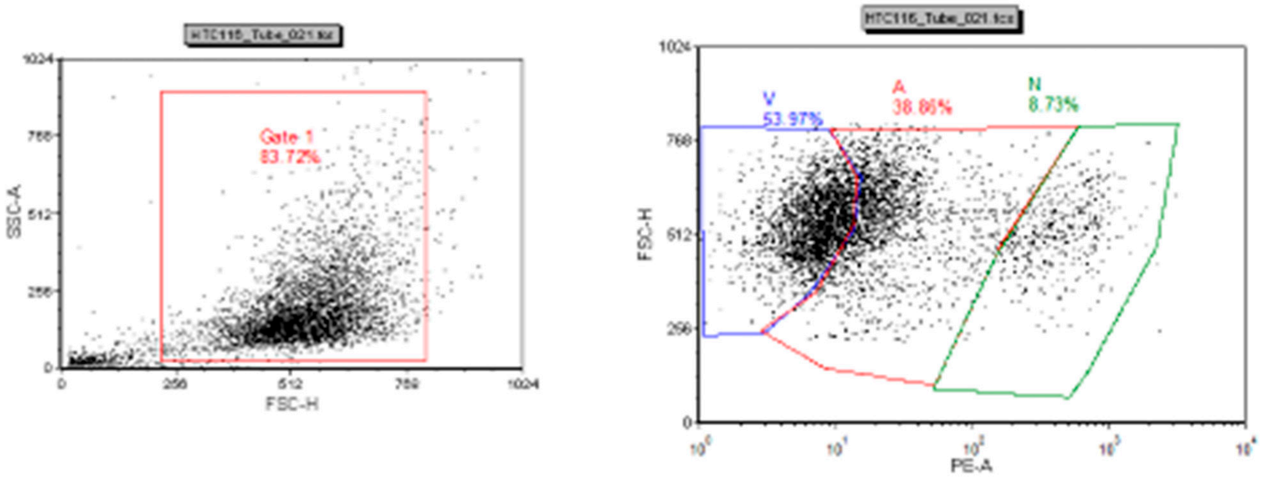

Overlay #	Filename	Gate	# of Events	X Geometric Mean	Y Geometric Mean	% of gated cells	% of all cells
1	HTC116_Tube_021.fcs	None	4982	16.41	535.84	100.00	83.72
1	HTC116_Tube_021.fcs	Gate 1	4982	16.41	535.84	100.00	83.72
1	HTC116_Tube_021.fcs	Gate 2	2689	7.29	539.67	53.97	45.19
1	HTC116_Tube_021.fcs	Gate 3	1936	24.72	542.18	38.86	32.53
1	HTC116_Tube_021.fcs	Gate 4	435	440.70	504.31	8.73	7.31

Figure S36. Flow cytometry analysis on HTC116 cells.
